# Weak SLP-76/PLC-γ1 interaction fine-tunes T cell receptor signal strength to optimize T cell responsiveness

**DOI:** 10.1038/s41467-026-75599-0

**Published:** 2026-07-29

**Authors:** Hidehiro Yamane, Junya Wada, Elizabeth N. Stassenko, Natalie D. Convertino, Mariah E. Lee, Melanie S. Vacchio, Wenmei Li, Ferenc Livak, Udumbara M. Rathnayake, Lakshmi Balagopalan, Raj Chari, Herbert Hagenau, Parirokh Awasthi, Dorian B. McGavern, Remy Bosselut, Lawrence E. Samelson

**Affiliations:** 1 Laboratory of Cellular and Molecular Biology, Center for Cancer Research (CCR), National Cancer Institute (NCI), National Institutes of Health (NIH), Bethesda, MD USA; 2 Laboratory of Integrative Cancer Immunology, CCR, NCI, NIH, Bethesda, MD USA; 3 Flow Cytometry Core, Laboratory of Genome Integrity, CCR, NCI, NIH, Bethesda, MD USA; 4 Genome Modification Core, Laboratory of Animal Sciences Program (LASP), Frederick National Laboratory for Cancer Research (FNLCR), NCI, NIH, Frederick, MD USA; 5 Mouse Modeling and Cryopreservation, LASP, FNLCR, NCI, NIH, Frederick, MD USA; 6 Viral Immunology & Intravital Imaging Section, Neuroimmunology & Neurovirology Division, National Institute of Neurological Disorders and Stroke, NIH, Bethesda, MD USA

**Keywords:** Lymphocyte activation, T-cell receptor

## Abstract

Upon T cell receptor (TCR) engagement, phosphorylation of the LAT adapter protein enables binding of the enzyme PLC-γ1 to a Gads/SLP-76 dimer, forming a tetrameric structure. The interaction between SLP-76 and PLC-γ1 is weak within this heterotetramer, and the relevant binding sites of SLP-76 and PLC-γ1 are highly conserved in vertebrates. We generated a mouse with a T cell-specific mutation in the SLP-76 that enhanced its affinity for PLC-γ1, thereby increasing PLC-γ1 activity and TCR signal strength. This mutation not only altered the development of αβTCR thymocytes, invariant NKT cells, and intraepithelial lymphocyte precursors but also impaired the generation of central memory CD8^+^ T cells upon acute viral infection and the humoral immune response mediated by germinal center T follicular helper T cells. These findings suggest that the conserved weak SLP-76/PLC-γ1 interaction is important for the controlled activation of PLC-γ1, thus fine-tuning TCR signal strength to optimize T cell-mediated immunity.

## Abstract (web summary)

Upon T cell receptor engagement with a peptide/MHC complex, multiple proteins assemble at the membrane adaptor, LAT. Here, the authors show that the conserved, weak SLP-76/PLC-γ1 interaction within this assembly acts as a critical checkpoint for T cell development and function.

## Introduction

The engagement of the T cell antigen receptor (TCR) with antigenic peptide bound to an MHC-encoded protein is the initiating event in the activation of T cells. Recruitment of critical protein tyrosine kinases (PTKs) including ZAP-70 to the intracellular domains of the TCR and activation of the PTKs rapidly follow. These PTKs have multiple enzyme and adapter protein substrates. We have focused on the consequences of the ZAP-70-mediated tyrosine phosphorylation of the integral membrane adapter protein LAT^1^. The phosphorylation of this molecule on four of its intracellular tyrosine residues creates binding sites for additional adapter molecules and enzymes, leading to activation of multiple intracellular events. Phospholipase C-γ1 (PLC-γ1) is one such critical enzyme. It translocates from the cytoplasm to the plasma membrane to bind phosphorylated LAT upon T cell activation. Additionally, a dimer of two cytosolic adapters Gads and SLP-76 also binds to phospho-LAT and PLC-γ1, thus creating a tetrameric protein complex, LAT/Gads/SLP-76/PLC-γ1^2,3^. Upon TCR-mediated activation multiple additional proteins including the adapters Nck and Vav and the enzyme ITK bind to phosphorylated SLP-76 at the LAT-nucleated tetrameric complex in order to phosphorylate and thus activate PLC-γ1 to target its substrate phosphatidylinositol 4,5-bisphosphate (PIP2) at the plasma membrane. Cleavage of this lipid results in the release of inositol trisphosphate (IP3), which mediates the elevation of intracellular calcium, and diacylglycerol which leads to the activation of PI-3 kinase and critical protein serine kinases^4^.

We have studied the formation of the tetrameric complex in vitro using recombinant and synthetic proteins, isothermal titration calorimetry (ITC) and analytical ultracentrifugation (AUC) to determine the thermodynamics of protein-protein interactions and tetramer formation^3,5^. The individual affinities of binding between phospho-LAT and Gads, Gads and SLP-76, and phospho-LAT and PLC-γ1 are in the sub-micromolar range, though the interaction of SLP-76 with PLC-γ1 is undetectable at room temperature and barely detected at 4 °C with a *K*_d_ of 3.3 µM^5^. Using all four proteins we subsequently demonstrated the formation of the tetrameric complex by AUC and showed that the concentration range of tetramer formation is estimated to be 0.1−1 µM^3^. We observed that the favorable decrease in enthalpy generated during tetramer formation mostly compensates for the entropic penalty resulting from the spatially limited circular arrangement of the tetramer^3^. We suggested that the tetramer is in thermodynamic equilibrium between open and closed circular structures regulated by the SLP-76/PLC-γ1 interaction. In a second study, we used liposome-based reconstitution to define the membrane recruitment and enzymatic activities of these proteins. Formation of the tetramer at the liposomal membrane enhances phosphoinositide hydrolysis by PLC-γ1 over that induced by PLC-γ1 alone^6^. These results led us to propose the model that the formation of a closed circular tetrameric structure mediated by the SLP-76/PLC-γ1 interaction induces a conformational change in PLC-γ1 that activates its enzymatic function.

Based on our previous findings, we hypothesized that the low affinity interaction between SLP-76 and PLC-γ1 in the setting of the tetramer might serve as a critical check point for T cell immune responses. In support of this hypothesis, the animo acid sequences of the interaction sites of SLP-76 and PLC-γ1 were highly conserved among jawed vertebrates. Therefore, we sought to determine the consequences of increasing the affinity of interaction between these two components by substituting the relevant region of SLP-76 with a short sequence of amino acids (aa) previously demonstrated to have a higher affinity for the PLC-γ1 SH3 domain^7^. We found that this higher affinity interaction increased the activity of PLC-γ1 in vitro. We then generated a mouse in which the higher affinity proline-rich region (PRR) of SLP-76 replaced the wild-type (WT) sequence specifically in T cells and observed impaired thymocyte development. Moreover, using TCR transgenic systems, we found that this mutation led to altered CD4^+^ and CD8^+^ T cell immune responses. We conclude that the low affinity interaction between SLP-76 and PLC-γ1 is an evolutionally conserved mechanism that underlies the fine-tuning of the strength of TCR-mediated signaling to optimize T cell development and T cell-mediated immunity.

## Results

### In vitro reconstitution and characterization of the SLP-76/PLC-γ1 interaction

**Fig. 1:**
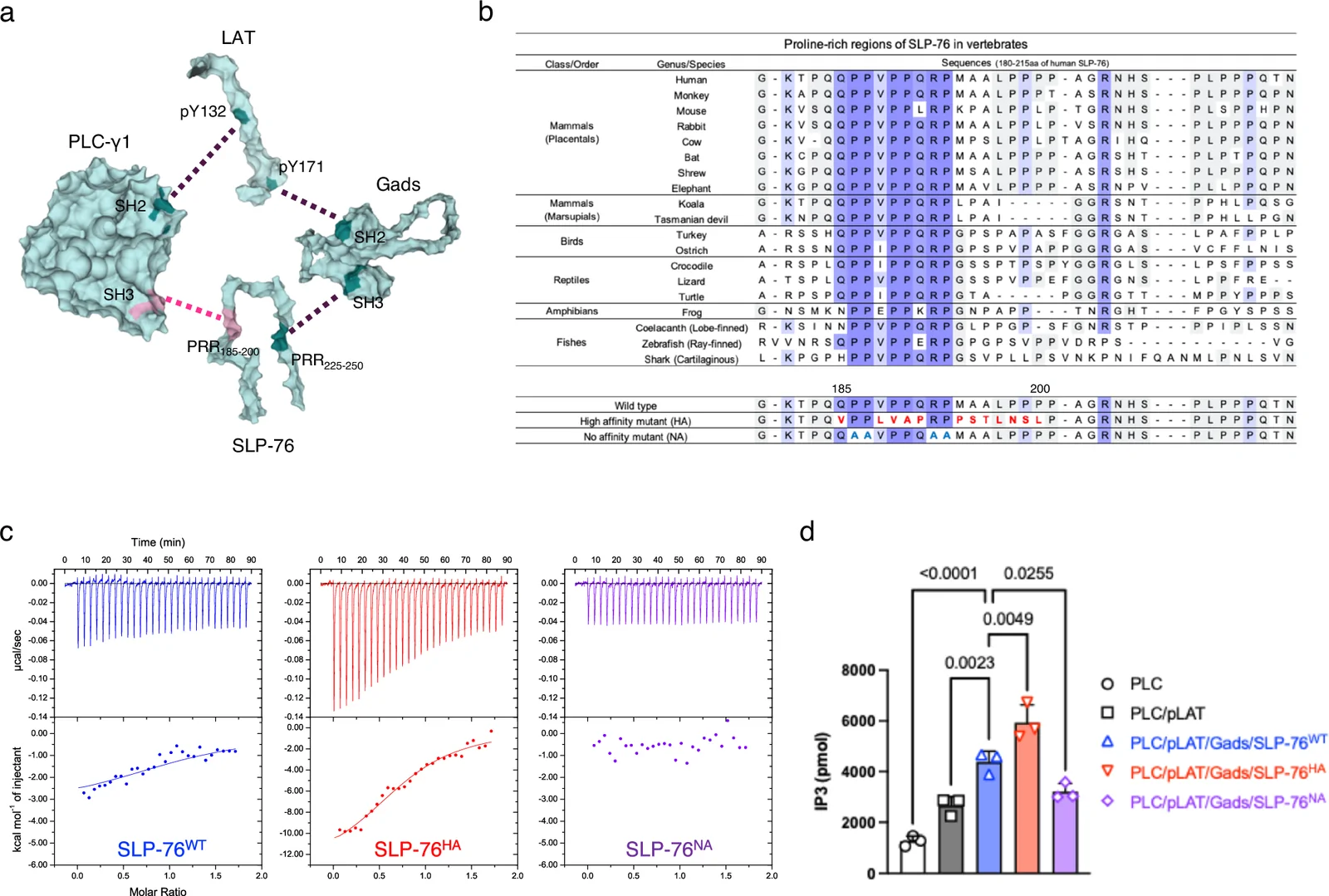
Characterization of the tetrameric protein complex containing LAT, Gads, SLP-76 and PLC-γ1 by in vitro reconstitution. **a** Schematic of tetrameric structure (dark cyan, strong interaction sites; dark red, weak interaction site between PLC-γ1 and SLP-76). The 3D structure of PLC-γ1 is based on PDB:6PBC^54,55^. The 3D model structure of LAT (aa 90–193), Gads (aa 1–330), and SLP-76 (aa 103–258) was generated by Alphafold3. **b** Sequence alignment of the proline-rich region in SLP-76 among 19 jawed vertebrates. Multiple residues shown in red are substituted amino acids in the high affinity mutant. Four alanines in cyan are substituted amino acids in the no affinity mutant. **c** Spectrum of isothermal titration calorimetry for the interaction between PLC-γ1 and SLP-76^WT^ (wild-type), SLP-76^HA^ (high affinity mutant), and SLP-76^NA^ (no affinity mutant), measured by VP-ITC. **d** The levels of IP3 determined by PLC-γ1 catalytic activity assay. Data were generated from three independent experiments and shown as mean ± SD (**d**). Statistical analysis was carried out using ordinary one-way ANOVA (**d**).

In previous studies of intracellular signaling downstream of the TCR, we used in vitro reconstitution techniques to demonstrate and verify that four signaling proteins, PLC-γ1, LAT, Gads, and SLP-76, form a heterotetrameric protein complex through the following interactions: the SH2 domain of PLC-γ1 and pY132 of LAT, the SH2 domain of Gads and pY171 of LAT, the PRR_225–250_ of SLP-76 and the SH3 domain of Gads, and the PRR_185–200_ of SLP-76 and the SH3 domain of PLC-γ1^3,5^ (Fig. 1a). Calorimetric data for the protein-protein interactions defining this tetramer are shown in Table 1. Of note is that the interaction between PLC-γ1 and SLP-76 is undetectable at room temperature and has a *K*_d_ of only 3345 nM at 4 °C^3,5^. To examine the significance of the interaction between SLP-76 and PLC-γ1 over evolution in more detail we compared the protein sequences of nineteen orthologs of SLP-76 and PLC-γ1 in jawed vertebrates, ranging from cartilaginous fish to placental mammals. These sequences were aligned by Protein BLAST (Fig. 1b and Supplementary Fig. 1a). The SH3 domain of PLC-γ1 binds to the PRR of SLP-76 (aa 180–215 from human sequence). In this PRR, two double prolines (PP) and an arginine-proline pair (RP) were perfectly conserved, which is consistent with the previous reports about the importance of the PXXP motif in this region^7,8^ (Fig. 1b). As this region interacts with the SH3 domain of PLC-γ1 (aa 791–851 from human sequence), we also aligned the sequences of vertebrate PLC-γ1 SH3 domains interacting with SLP-76 and found near perfect conservation (Supplementary Fig. 1a). The sequence conservation of the relevant regions of the SLP-76 PRR and the PLC-γ1 SH3 domain suggest a crucial role of this protein-protein interaction during the cooperative activation of PLC-γ1 by tetramer formation upon TCR engagement^3,5^.

**Table 1:** Thermodynamic parameters of individual protein-protein interactions determining tetramer formation among LAT, Gads, SLP-76 and PLC-γ1

Interactions (Protein 1/Protein 2)	Size of protein 1	Size of protein 2	*K*_d_ (nM)	Temp. (˚C)	DH (kcal/mol)	-TDS (kcal/mol)	DG (kcal/mol)	Reference
PLC-γ1/LAT	550–846	122–140 ^pY132^	62	25	−15.7 ± 0.3	6.0 ± 0.3	−9.8 ± 0.2	#5
	1–1219	125–178 ^pY132, pY171^	84	20	−5.4	−4.1	−9.5	#3
Gads/LAT	1–322	161–180 ^pY171^	164	25	−9.6 ± 0.6	0.3 ± 0.5	−9.2 ± 0.1	#5
	1–322	125–178 ^pY132, pY171^	213	20	−10.7	1.7	−9.0	#3
Gads/SLP-76	1–322	157–420 (WT)^1^	19	25	−12.0 ± 1.7	1.7 ± 1.8	−10.4 ± 0.3	#5
	1–322	159–533 (WT)	3.6	20	−15.0	4.6	−10.4	#3
PLC-γ1/SLP-76	550–846	157–420 (WT)	ND	25	ND	ND	ND	#5
	550–846	157–420 (WT)	3345	4	−1.8 ± 0.1	−5.6 ± 0.1	−7.3 ± 0.0	#5
	1–1219	159–533 (WT)	ND	20	ND	ND	ND	#3
PLC-γ1/SLP-76	1–1219	103–258 (WT)	2317 ± 853	5	−3.8 ± 1.0	−3.4 ± 1.2	−7.2 ± 0.3	This study
	1–1219	103–258 (HA)^2^	895 ± 100	5	−10.9 ± 2.3	3.2 ± 2.3	−7.7 ± 0.1	This study
	1–1219	103–258 (NA)^3^	ND	5	ND	ND	ND	This study
	1–1219	185–200 (WT)	3989 ± 1712	5	−3.3 ± 0.7	−3.6 ± 0.9	−6.9 ± 0.3	This study
	1–1219	185–200 (HA)	1116 ± 389	5	−10.3 ± 1.2	2.7 ± 1.3	−7.6 ± 0.3	This study
	1–1219	185–200 (NA)	ND	5	ND	ND	ND	This study
		1. Wild Type (WT)			ND: Not detectable			
		2. High Affinity mutant (HA)						
		3. No Affinity mutant (NA)						

This thermodynamic parameter table combines data from our previous studies^3,5^ and the current study. In all ITC analyses, LAT or SLP-76 was in the syringe and injected into PLC-γ1 or Gads in the cell. The standard deviations shown in the data from the current study were calculated from three independent experiments.

To evaluate whether an alteration of the affinity between SLP-76 and PLC-γ1 could modulate the activation of PLC-γ1 in the context of the tetrameric complex, we designed two SLP-76 mutants and compared them to wild-type SLP-76 (SLP-76^WT^) in the formation of the tetrameric complex and PLC-γ1 activation. The sequence changes employed to create the high affinity (SLP-76^HA^) and no affinity mutant of SLP-76 (SLP-76^NA^) were indicated in Fig. 1b. The sequence of SLP-76^HA^ was obtained from previous screening data for the ligand preferences of the SH3 domain of PLC-γ1 using a phage-display library^7^. SLP-76^NA^ was newly designed as a negative mutant based on the alignment and previous research^8^. Purity of the three SLP-76 polypeptides was confirmed by SDS-PAGE (Supplementary Fig. 1b). We performed the ITC assay at 5˚C to compare the thermodynamic parameters of binding to full length PLC-γ1 between the WT and SLP-76 PRR mutants (Fig. 1c and Table 1). The interaction of the SLP-76^WT^ polypeptide (aa 103–258) with PLC-γ1 had a *K*_d_ value of 2317 nM and produced a small heat change (−3.8 kcal/mol), which was consistent with our previous study^3,5^. *K*_d_ value differences between our previous and current studies are likely because we now used full length PLC-γ1 instead of the PLC-γ1 SH3 domain alone. The affinity of SLP-76^HA^ was 895 nM with a heat change (−10.9 kcal/mol), indicating that the high affinity mutation strengthens the binding of SLP-76 to PLC-γ1 and suggesting that this decrease in the *K*_d_ value is due to an increased change in enthalpy (ΔH). The SLP-76^NA^ showed no interaction with PLC-γ1, and consequently the *K*_d_ value could not be determined. We also employed a set of short peptides from the SLP-76 PRR (aa 185–200). Their interaction with PLC-γ1 was of similar but lower affinity than the longer SLP-76_103–258_ polypeptides (Table 1 and Supplementary Fig. 1c).

It was previously reported that the SH3 domains of Itk and Lck also interact with the SLP-76 PRR^8–13^. We performed sequence alignments of regions of the three SH3 domains, Itk_171–231_, Lck_61–121_ and, as above, PLC-γ1_791–851_ and found that the former two sequences lacked two of the four amino acids (indicated in red) critical for binding to the SLP-76 PRR (Supplementary Fig. 1d). We then carried out ITC assays to assess if this region of the Itk SH3 domain might bind to the three different forms of the SLP-76 PRR_185–200_ peptides and found that full-length Itk was unable to interact with the SLP-76 PRR (Supplementary Fig. 1e and Supplementary Table 1). The conserved proline-rich PIPRSP motif in LAT_54–90_ has recently been shown to associate with the Lck SH3 domain^14^. Our sequence alignment analysis revealed no similar feature between that LAT proline-rich motif and SLP-76 PRR_180–207_ (Supplementary Fig. 1f). Under the conditions of our analysis, we concluded that the SH3 domain of PLC-γ1 would be the major binding partner of the SLP-76 PRR_185–200_.

Next, we assessed the impact of the three SLP-76 PRR sequences on activation of PLC-γ1 in vitro using liposomes containing PIP2, the PLC-γ1 substrate^6^. Recruitment of PLC-γ1 to liposomes containing phospho-LAT alone, proteins interacting with a *K*_d_ of 62 nM, caused an activation of PIP2 hydrolysis of 2.1-fold compared with liposomes incubated with PLC-γ1 alone (Fig. 1d: white vs. gray). This enhancement was consistent with our previous reconstitution study^6^. Reconstitution of the LAT/Gads/SLP-76^WT^/PLC-γ1 tetramer further enhanced PLC-γ1 activity on PIP2-bearing liposomes by 1.7-fold, indicating the importance of the SLP-76/PLC-γ1 interaction in the tetramer despite its low affinity with a *K*_d_ of 2317 nM (Fig. 1d: gray vs. blue). Substitution of SLP-76^WT^ with SLP-76^HA^ further increased the activity by 1.4-fold, and the tetramer with SLP-76^HA^ activated the function of PLC-γ1 by 4.6-fold in total (Fig. 1d: blue vs. red and white vs. red, respectively). In contrast, substitution with SLP-76^NA^ decreased the activity to that induced in the presence of PLC-γ1 and phospho-LAT without the Gads/SLP-76 dimer (Fig. 1d: gray vs. purple). These results confirm that the interaction of the SLP-76 PRR and the PLC-γ1 SH3 domain regulate PLC-γ1. Enhancing the affinity of the SLP-76/PLC-γ1 interaction likely leads to an increase in the frequency and/or duration of the closed circular tetramer formation, thereby enhancing PLC-γ1 activity.

### The SLP-76^HA^ mutation alters the development of αβTCR^+^ T cells in the thymus

SLP-76 is expressed in the T cell lineage as well as in other cell types including myeloid cells, granulocytes, mast cells, NK cells, and platelets^15^. To evaluate the biological significance of the weak SLP-76/PLC-γ1 interaction in T cells under physiological settings, we created a mouse line in which the WT SLP-76 PRR_185–200_ was conditionally replaced with the SLP-76^HA^ (Supplementary Fig. 2a). A targeting vector was constructed consisting of WT SLP-76 cDNA spanning from exons 8 through 21 (SLP-76 cDNA Ex. 8–21) followed by a polyadenylation (poly A) sequence, which was flanked by LoxP sequences. The mutated exon 8 encoding the SLP-76^HA^ followed the second LoxP sequence. Using CRISPR/Cas9-mediated gene editing, the exon 8 of the *Lcp2* gene (encoding the SLP-76 protein) was replaced with the targeting vector. As the transcription of the *Lcp2*^*L8:21L-mE8*^ allele terminated at the poly A sequence without Cre recombinase activity, this recombineered allele was expected to produce WT SLP-76 protein. Cre-mediated excision of the LoxP-flanked DNA sequence caused the mutated exon 8 and the downstream exons 9 through 21 to follow the exon 7 (*Lcp2*^*mE8*^ allele). The *Lcp2*^*mE8*^ allele was expected to produce full-length SLP-76 protein carrying the SLP-76^HA^ mutation (Supplementary Fig. 2a).

**Fig. 2:**
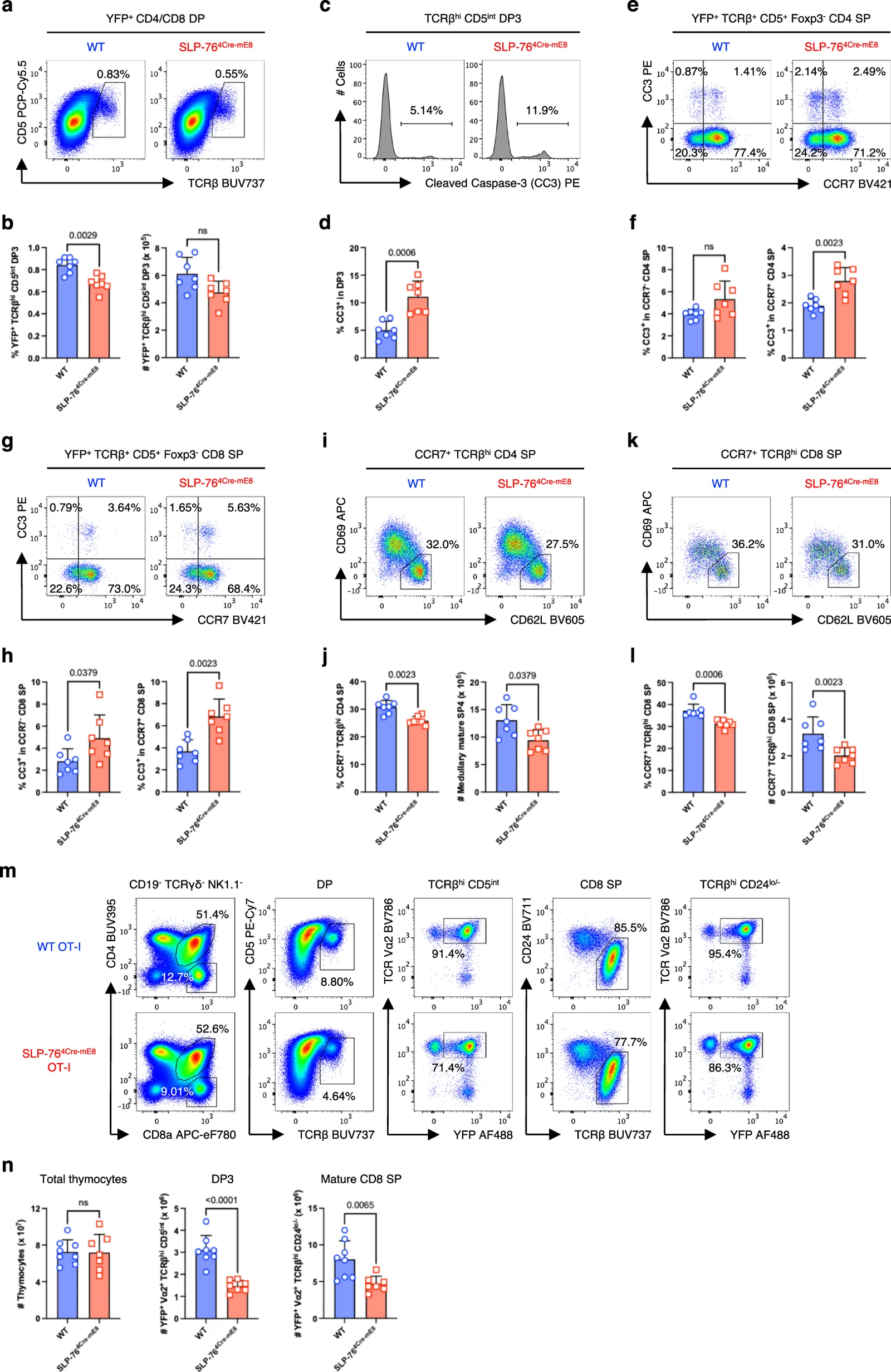
SLP-76^HA^ mutation alters the development of αβTCR^+^ thymocytes. **a** Representative pseudocolor plot analysis of WT and SLP-76^4Cre-mE8^ DP thymocytes for a TCRβ^hi^ CD5^int^ DP3 subset. **b** Statistical analysis for the frequency (left) and number (right) of DP3 thymocytes (*n* = 7 for each group). **c** Representative histogram analysis of WT and SLP-76^4Cre-mE8^ DP3 thymocytes for the frequency of cleaved Caspase-3 (CC3) expression. **d** Statistical analysis for the frequency of CC3^+^ DP3 thymocytes (*n* = 7 for each group). Representative pseudocolor plot analysis of WT and SLP-76^4Cre-mE8^ TCRβ^+^ CD5^+^ CD4 SP (**e**) and CD8 SP (**g**) thymocytes for CCR7 and CC3 expression. Numbers indicate the frequency of cells in each quadrant. Statistical analysis for the frequency of CC3^+^ cells in CCR7^-^ (left) and CCR7^+^ (right) CD4 SP (**f**) and CD8 SP (**h**) thymocytes (*n* = 7 for each group). Representative pseudocolor plot analysis of WT and SLP-76^4Cre-mE8^ CCR7^+^ TCRβ^hi^ CD4 SP (**i**) and CD8 SP (**k**) thymocytes for CD62L and CD69 expressions. Statistical analysis for the frequency (left) and number (right) of CD62L^+^ CD69^-^ most mature (M2) CD4 SP (**j**) and CD8 SP (**l**) thymocytes (*n* = 7 for each group). **m** Gating strategy to analyze for TCRβ^hi^ CD5^int^ YFP^+^ TCRVα2^+^ DP3 and YFP^+^ TCRβ^hi^ CD24^lo/-^ mature CD8 SP thymocytes from WT (upper row) and SLP-76^4Cre-mE8^ (lower row) OT-I mice. **n** Statistical analysis of WT (*n* = 8) and SLP-76^4Cre-mE8^ (*n* = 7) OT-I mice for the number of total (left), DP3 (middle), and mature CD8 SP (right) thymocytes. Each symbol represents one mouse, and data are shown as mean ± SD. Statistical analysis was carried out using the Mann-Whitney test (**b**, **d**, **f**, **h**, **j**, **l**, **n**). ns not significant.

To examine the effect of the SLP-76^HA^ mutation on the development of αβTCR^+^ T cells in the thymus, *Lcp2*^*WT*^ and *Lcp2*^*L8:21L-mE8*^ mice were crossed onto *CD4*^*Cre*^ mice^16^. Cre-mediated gene manipulation is not always lineage-specific and/or complete^17^. Therefore, the resultant mice were further mated with *Rosa26*^*LSL-eYFP*^ fate-mapping reporter mice to induce YFP expression in cells with Cre recombinase activity, thus marking the SLP-76^HA^-expressing cells^18^. At 8–12 weeks of age, total thymocyte profiles including the frequency and the number of double-negative (DN), DP, CD4 single-positive (SP), and CD8 SP cells were grossly normal in *Lcp2*^*L8:21L-mE8*^ x *Rosa26*^*LSL-eYFP*^ x *CD4*^*Cre*^ (SLP-76^4Cre-mE8^) mice, with a slight increase in the frequency of DN thymocytes (Supplementary Fig. 2b, c). However, SLP-76^4Cre-mE8^ YFP^+^ DP thymocytes showed a slight but significant decrease in the frequency of post-positive selection TCRβ^high^ CD5^int^ DP3 cells^19^ (Fig. 2a, b). This finding was accompanied by an increased frequency of DP3 thymocytes expressing cleaved Caspase-3 (CC3) in SLP-76^4Cre-mE8^ mice (Fig. 2c, d). We confirmed that CD4^Cre^-mediated SLP-76^HA^ mutation was completed in YFP^+^ DP3 thymocytes, and that SLP-76^4Cre-mE8^ YFP^+^ DP3 thymocytes expressed a comparable level of SLP-76 protein with the WT counterpart (Supplementary Fig. 2d–f). These results indicate that the SLP-76^HA^ mutation promotes the apoptosis of DP thymocytes that normally survive after positive selection, presumably due to enhanced TCR signal strength during the recognition of MHC.

Next, we assessed whether the SLP-76^HA^ mutation would affect negative selection of CD4 and CD8 SP thymocytes. As with DP3 thymocytes, both YFP^+^ CD4 SP and CD8 SP thymocytes completed the CD4^Cre^-mediated SLP-76^HA^ mutation and expressed a comparable level of SLP-76 protein (Supplementary Fig. 2d–f). YFP^+^ TCRβ^+^ CD5^+^ Foxp3^-^ conventional CD4 and CD8 SP thymocytes that had signaled via a TCR were subdivided based on CCR7 expression. CCR7 is required for positively selected CD4 and CD8 SP thymocytes to migrate from cortex to medulla, a major niche for negative selection^20,21^. SLP-76^4Cre-mE8^ mice showed a slight but not significant increase in the frequency of CC3^+^ cells in CCR7^-^ CD4 SP thymocytes but had a significant enhancement of that in CCR7^+^ CD4 SP thymocytes (Fig. 2e, f and Supplementary Fig. 2g). Moreover, the frequency of CC3^+^ cells was increased in both CCR7^-^ and CCR7^+^ CD8 SP thymocytes from SLP-76^4Cre-mE8^ mice (Fig. 2g, h and Supplementary Fig. 2g). Consistent with these results, SLP-76^4Cre-mE8^ mice showed a significant decrease in the frequency and number of CCR7^+^ TCRβ^high^ CD62L^+^ CD69^-^ medullary CD4 and CD8 SP most mature (M2) thymocytes that had completed negative selection^22,23^ (Fig. 2i–l). In SLP-76^4Cre-mE8^ mice carrying a transgene of OT-I TCR which has a relatively high affinity for self-ligands, a profound decrease in both DP3 and mature CD8 SP thymocytes was evident without any influence on total thymocyte number, compared to those with endogenous TCR repertoire (Fig. 2m, n and Supplementary Fig. 2h). These results indicate that the SLP-76^HA^ mutation promotes the apoptosis of CD4 and CD8 SP thymocytes that normally pass the negative selection, presumably due to enhanced TCR signal strength during the recognition of self-peptide/MHC.

### The SLP-76^HA^ mutation promotes the development of agonist-selected invariant NKT cells and intraepithelial lymphocyte precursors in the thymus

**Fig. 3:**
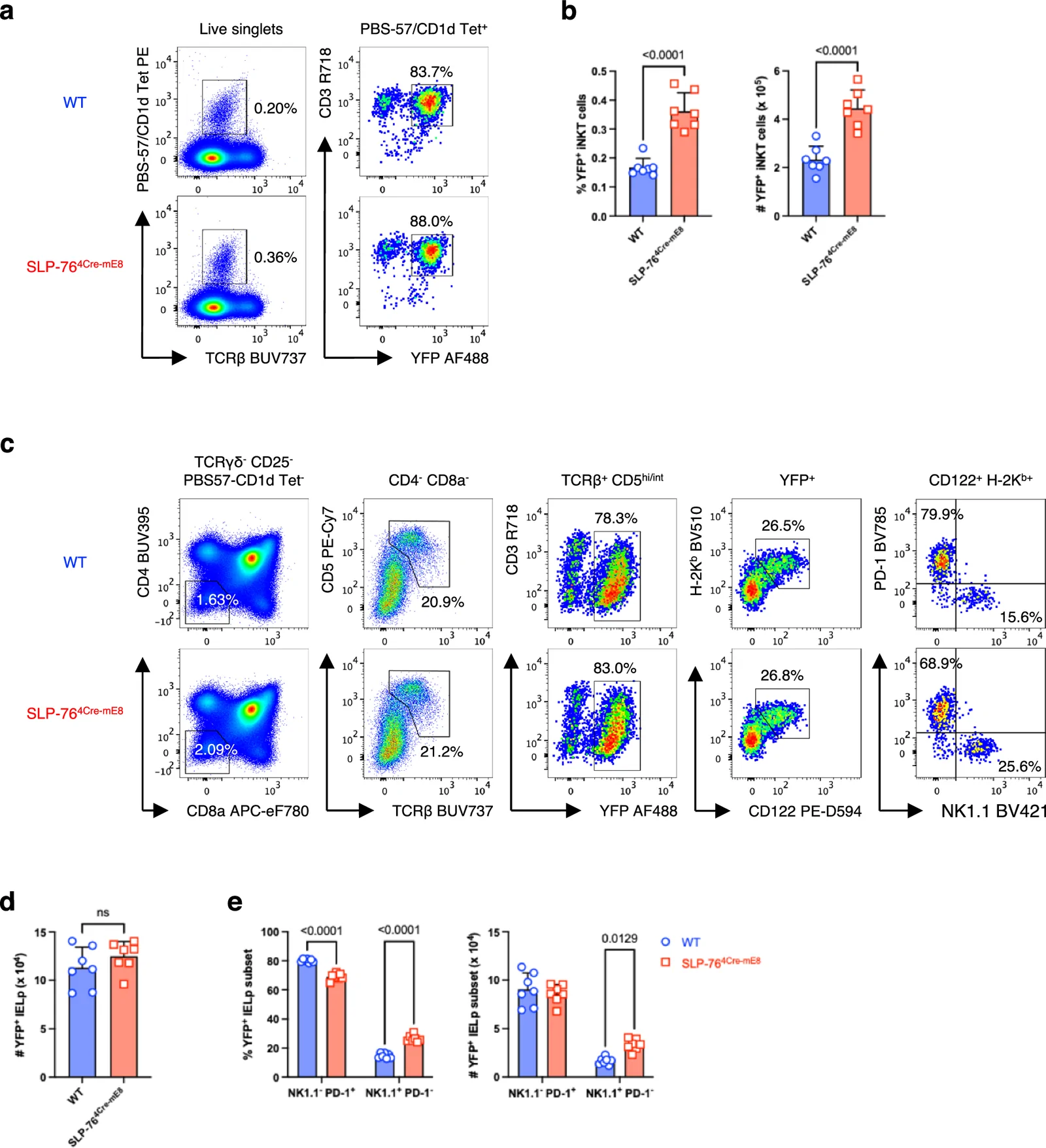
SLP-76^HA^ mutation enhances the development of invariant NKT cells and intraepithelial lymphocyte precursors in the thymus. **a** Representative pseudocolor plot analysis of WT and SLP-76^4Cre-mE8^ thymocytes for invariant NKT (iNKT) cells. **b** Statistical analysis for the frequency (left) and number (right) of iNKT cells (*n* = 7 for each group). **c** Representative pseudocolor plot analysis of WT and SLP-76^4Cre-mE8^ DN thymocytes for intraepithelial lymphocyte precursors (IELp). **d** Statistical analysis for the number of IELp (*n* = 7 for each group). **e** Statistical analysis for the frequency (left) and number (right) of type A (NK1.1^-^ PD-1^+^) and type B (NK1.1^+^ PD-1^-^) IELp subsets (*n* = 7 for each group). Each symbol represents one mouse, and data are shown as mean ± SD. Statistical analysis was carried out using the Mann-Whitney test (**b**, **d**) and two-way ANOVA (**e**). ns not significant.

The strength of TCR signaling during positive and negative selection is one of the key factors regulating the development of αβ T cells^24^. Additionally, some T cell lineages with innate-like properties including invariant NKT (iNKT) cells and intraepithelial lymphocyte precursors (IELp) require relatively high affinity stimulation through TCR for their development^25^. Thus, we evaluated the effect of the SLP-76^HA^ mutation on the development of these cells. We found that the frequency and number of TCRβ^int^ PBS-57/CD1d tetramer-positive CD3^+^ YFP^+^ iNKT cells were increased in the thymus of SLP-76^4Cre-mE8^ mice by more than 2-fold (Fig. 3a, b). There was no difference between WT and SLP-76^4Cre-mE8^ mice in the number of YFP^+^ IELp (TCRγδ^-^ CD25^-^ PBS-57/CD1d tetramer-negative DN thymocytes expressing TCRβ, CD5, CD122, and H-2K^b^) (Fig. 3c, d). IELp can be subdivided into T-bet^-^ NK1.1^-^ PD-1^+^ Type A and T-bet^+^ NK1.1^+^ PD-1^-^ Type B IELp subsets that localize differently within the thymus and have differential TCR specificities^26^. The frequency of NK1.1^-^ PD-1^+^ Type A IELp was slightly but significantly decreased in SLP-76^4Cre-mE8^ mice, whereas the number of these cells was comparable to that in WT mice (Fig. 3c, e). In contrast, the frequency and number of NK1.1^+^ PD-1^-^ Type B IELp were increased in SLP-76^4Cre-mE8^ mice by approximately 3-fold (Fig. 3c, e). These results indicate that the SLP-76^HA^ mutation promotes the development of iNKT cells and Type B IELp.

### The SLP-76^HA^ mutation promotes the β-selection of thymocytes

DN3 thymocytes that successfully rearrange the *Tcrb* locus to transmit signals through a pre-TCR composed of pre-TCRα and TCRβ chains for lineage commitment into αβTCR thymocytes, referred to as β-selection. To examine the effect of the SLP-76^HA^ mutation on the β-selection, *Lcp2*^*L8:21L-mE8*^ x *Rosa26*^*LSL-eYFP*^ mice were crossed onto *proximal Lck (pLck)*^*Cre*^ mice^27^. We first noted that the pLck promoter drove promiscuous expression of Cre recombinase, as judged by the expression of a YFP fate-mapping reporter, and about 30% of WT and *Lcp2*^*pLCre-mE8*^ mice expressed YFP in a T cell-specific manner (Supplementary Fig. 3a, b). Using the mice with T cell lineage-specific Cre activity, we found that a DN4/DN3 ratio within YFP^+^ DN thymocytes was elevated in *Lcp2*^*pLCre-mE8*^ mice (Supplementary Fig. 3c, d). Moreover, the frequency and number of a TCRβ^+^ CD5^int/hi^ population within YFP^+^ DN4 thymocytes were also increased in *Lcp2*^*pLCre-mE8*^ mice (Supplementary Fig. 3c, e). These results indicate that the SLP-76^HA^ mutation promotes the process of β-selection during the transition from DN3 to DN4 stages.

### Enhancement of memory-phenotype T cells in the periphery of SLP-76^4Cre-mE8^ mice

**Fig. 4:**
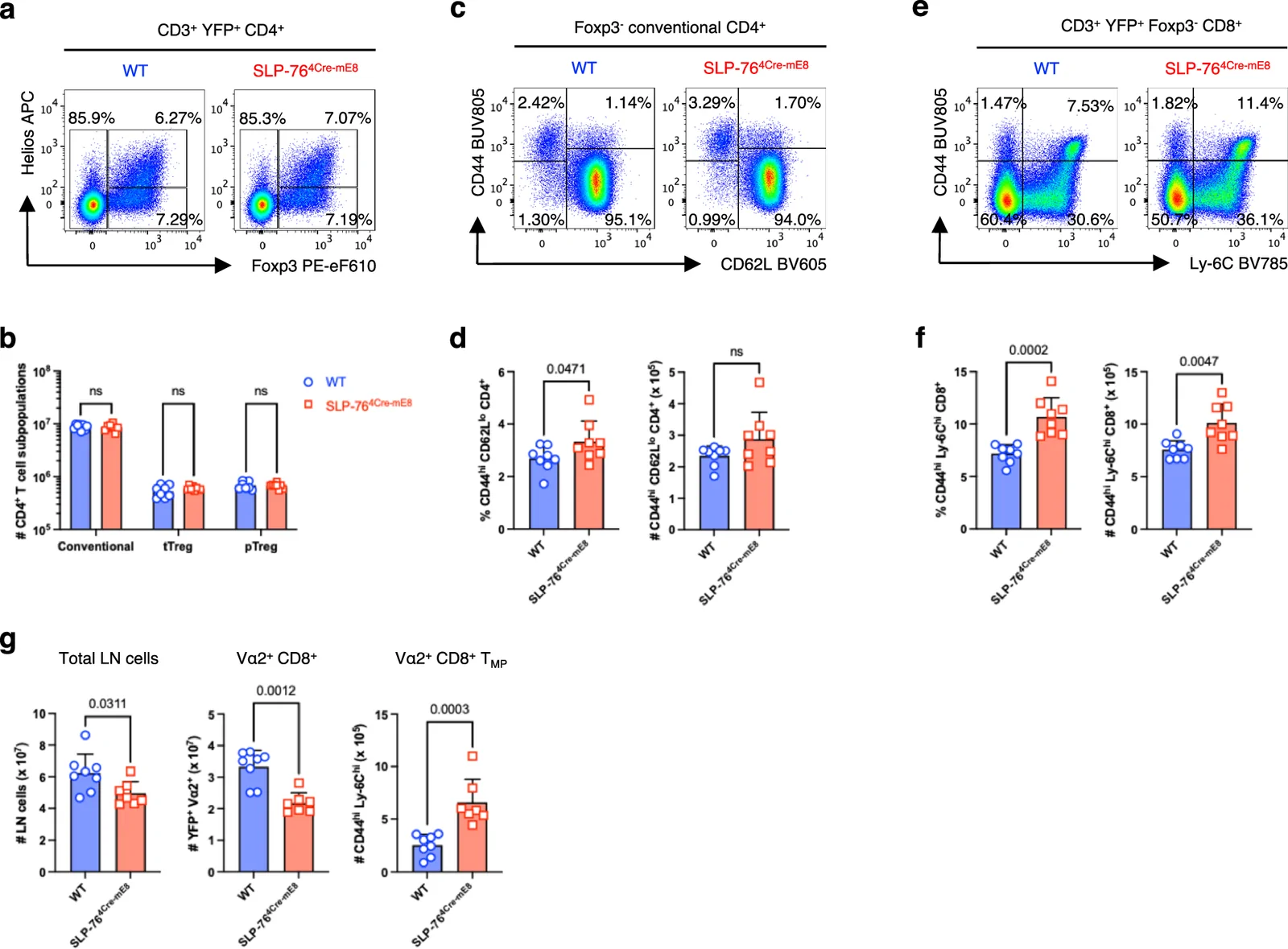
Characterization of peripheral T cells in SLP-76^4Cre-mE8^ mice. **a** Representative pseudocolor plot analysis of CD3^+^ YFP^+^ CD4^+^ T cells from pooled whole LNs for Foxp3 and Helios expression. **b** Statistical analysis for the number of Foxp3^-^ conventional CD4^+^ T cells, Foxp3^+^ Helios^+^ thymic-derived Tregs (tTregs), and Foxp3^+^ Helios^-^ peripherally induced Tregs (pTregs) (*n* = 8 for each group). **c** Representative pseudocolor plot analysis of conventional CD4^+^ T cells for CD62L and CD44 expression. **d** Statistical analysis for the frequency (left) and number (right) of CD44^hi^ CD62L^lo^ memory-phenotype CD4^+^ T (T_MP_) cells (*n* = 8 for each group). **e** Representative pseudocolor plot analysis of conventional CD8^+^ T cells for Ly-6C and CD44 expression. **f** Statistical analysis for the frequency (left) and number (right) of CD44^hi^ Ly-6C^hi^ CD8^+^ T_MP_ cells (*n* = 8 for each group). **g** Statistical analysis of WT (*n* = 8) and SLP-76^4Cre-mE8^ (*n* = 7) OT-I mice for the number of total LN cells (left), YFP^+^ TCRVα2^+^ CD8^+^ T cells (middle), and YFP^+^ TCRVα2^+^ CD8^+^ T_MP_ (right) cells. Each symbol represents one mouse, and data are shown as mean ± SD. Statistical analysis was carried out using two-way ANOVA (**b**) and the Mann-Whitney test (**d**, **f**, **g**). ns not significant.

We assessed whether the SLP-76^HA^ mutation would alter the phenotype of peripheral CD4^+^ and CD8^+^ T cells in pooled whole lymph nodes (LNs; axillary, brachial, superficial/deep cervical, inguinal, and mesenteric) of 8–12-week-old mice in a steady state. CD3^+^ YFP^+^ CD4^+^ T cells were subdivided into three populations based on Foxp3 and Helios expressions: Foxp3^-^ conventional T cells, Foxp3^+^ Helios^+^ thymic-derived regulatory T cells (tTregs), and Foxp3^+^ Helios^-^ peripherally induced regulatory T cells (pTregs)^28^. The frequency and number of all three populations were unaffected in SLP-76^4Cre-mE8^ mice (Fig. 4a, b and Supplementary Fig. 4a, b). Moreover, CD44^low^ CD62L^high^ central Tregs (cTregs) and CD44^high^ CD62L^low^ effector Tregs (eTregs) in both tTregs and pTregs were comparable between WT and SLP-76^4Cre-mE8^ mice, except a slight increase in the number of eTregs in tTregs^29–31^ (Supplementary Fig. 4c–e). These results indicate that the SLP-76^HA^ mutation barely influences the homeostasis and functional property of Tregs. Subsequent analysis of conventional CD4^+^ T cells revealed that SLP-76^4Cre-mE8^ mice had a mild increase in the frequency of CD44^high^ CD62L^low^ memory-phenotype T (T_MP_) cells that arise from naïve CD4^+^ T cells upon recognition of a self-peptide/MHC II complex^32^ (Fig. 4c, d). Additionally, SLP-76^4Cre-mE8^ mice had a marked increase in the frequency and absolute number of CD44^high^ Ly-6C^high^ CD8^+^ T_MP_ cells that also expressed CD62L and CD122/IL-2Rβ chain^33,34^ (Fig. 4e, f and Supplementary Fig. 4f, g). Notably, SLP-76^4Cre-mE8^ mice on an OT-I TCR transgenic background had a decrease in the number of total LN cells and YFP^+^ TCRVα2^+^ CD8^+^ T cells but a 2-fold increase in CD44^high^ Ly-6C^high^ T_MP_ cells due to a large increase in the frequency of this population (Fig. 4g and Supplementary Fig. 4h–j). These results suggest that when naïve CD4^+^ and CD8^+^ T cells recognize self-peptide/MHC complexes in the secondary lymphoid organs, enhanced TCR signal strength as a consequence of the SLP-76^HA^ mutation leads to increased generation of T_MP_ cells.

### The SLP-76^HA^ mutation enhances the responsiveness of OT-I CD8^+^ T cells to OVA altered peptide ligands with lower affinity in vitro and in vivo

We next examined whether the SLP-76^HA^ mutation would influence the degree of activation of peripheral T cells. To this end, *Lcp2*^*L8:21L-mE8*^ x *Rosa26*^*LSL-eYFP*^ mice were crossed onto *hCD2*^*Cre*^ (SLP-76^2Cre-mE8^) mice, which excises the LoxP-flanked SLP-76 cDNA Ex. 8–21 and the poly A sequence after negative selection, thus bypassing the effect of the SLP-76^HA^ mutation on thymocyte development^35^. *Lcp2*^*WT*^ × *Rosa26*^*LSL-eYFP*^ mice bred onto *hCD2*^*Cre*^ mice were used as a WT control. Both YFP^+^ CD4^+^ and CD8^+^ T cells from SLP-76^2Cre-mE8^ mice completed the Cre-mediated SLP-76^HA^ mutation and expressed a comparable level of SLP-76 protein (Supplementary Fig. 5a–c). Notably, in the absence of Cre recombinase, the *Lcp2*^*L8:21L-mE8*^ allele produced approximately a half as much SLP-76 protein as did the *Lcp2*^*WT*^ allele (Supplementary Fig. 5b, c). However, this decreased expression of SLP-76 did not affect the peripheral T cell pool in the *Lcp2*^*L8:21L-mE8*^ mice (Supplementary Fig. 5d). The resultant mice were further crossed onto OT-I TCR transgenic mice, to enable use of the variety of OVA_257–264_ altered peptide ligands (APLs) with various affinity for an OT-I TCR to evaluate the strength of TCR-mediated signaling in CD8^+^ T cells^36,37^. WT OT-I and SLP-76^2Cre-mE8^ OT-I mice were bred on a CD45.1 and CD45.2 background, respectively, to distinguish OT-I CD8^+^ T cells of the two different genotypes by flow cytometry^38^.

**Fig. 5:**
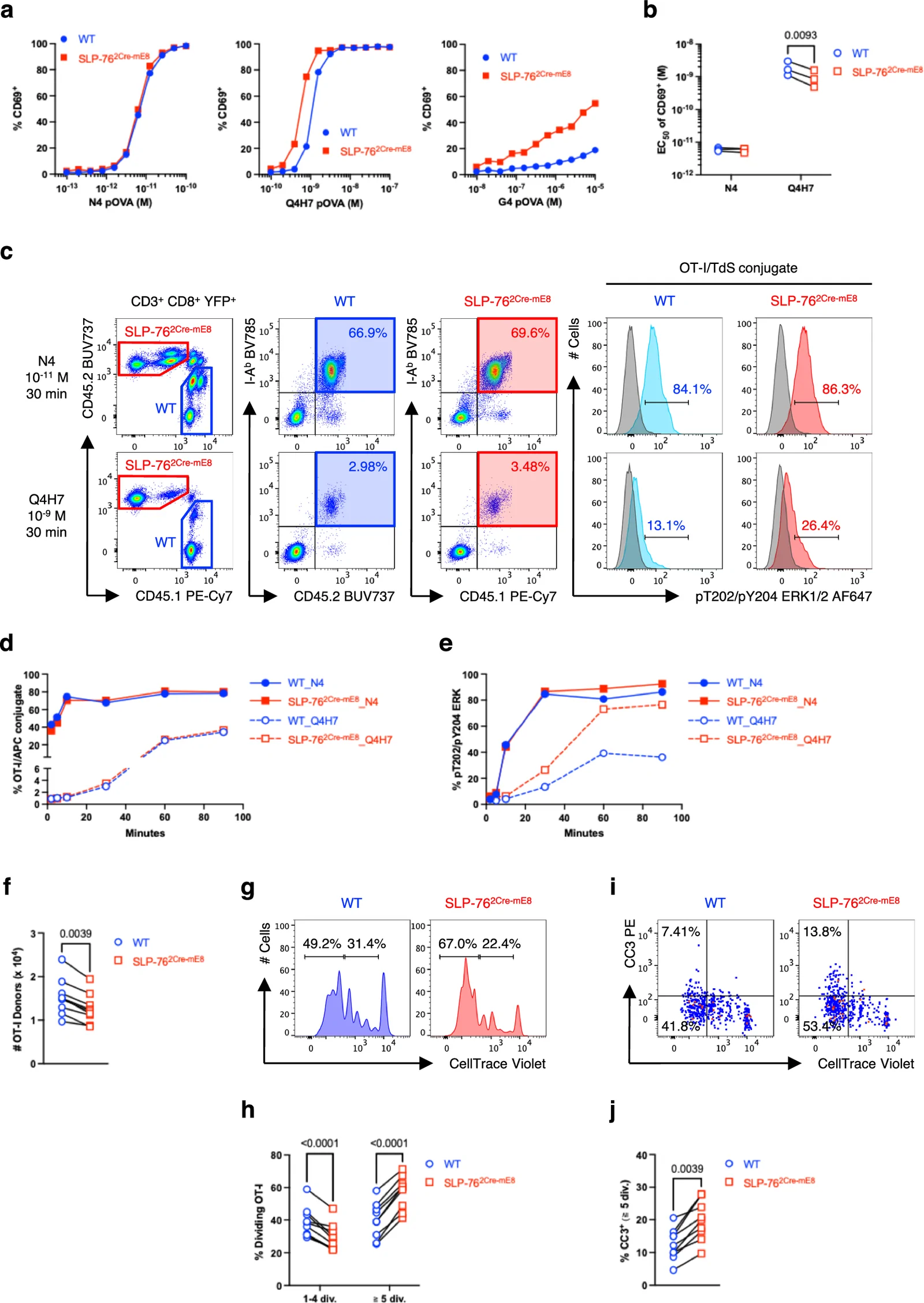
SLP-76^HA^ mutation enhances the responsiveness of OT-I CD8^+^ T cells to OVA altered peptide ligands with lower affinity in vitro and in vivo. **a** The frequency of CD69^+^ WT (CD45.1) and SLP-76^2Cre-mE8^ (CD45.2) OT-I cells stimulated for 20 h with varying concentrations of N4 (left), Q4H7 (middle), and G4 (right) peptide loaded on (CD45.1 × CD45.2) F1 TdS cells. **b** Statistical analysis for EC_50_ of CD69^+^ OT-I cells in response to N4 or Q4H7 peptide from three independent experiments. Symbols connected with a line indicate data from the same experiment. **c** Gating strategy to assess the level of phospho-ERK (pERK) in WT (CD45.1) and SLP-76^2Cre-mE8^ (CD45.2) OT-I cells stimulated for 30 min with N4 (10^−11 ^M) or Q4H7 (10^−9 ^M) peptide loaded on (CD45.1 × CD45.2) F1 TdS cells. **d** Kinetics of the frequency of WT and SLP-76^2Cre-mE8^ OT-I cells interacting with N4- or Q4H7-loaded TdS cells. **e** Kinetics of the frequency of pERK^+^ WT and SLP-76^2Cre-mE8^ OT-I cells interacting with N4- or Q4H7-loaded TdS cells. Shown is a representative set of data from 2 independent experiments. **f**–**j** CTV-labeled WT CD45.1 and SLP-76^2Cre-mE8^ CD45.2 OT-I CD8^+^ T cells were co-transferred into (CD45.1 × CD45.2) F1 mice. One day later, recipient mice (*n* = 9) were subcutaneously immunized with Q4H7 in CFA. Donor OT-I cells in skin dLNs were analyzed at 3 days post-immunization. **f** Statistical analysis for the number of WT and SLP-76^2Cre-mE8^ OT-I donor cells. **g** Representative histogram analysis for division of WT and SLP-76^2Cre-mE8^ OT-I donor cells. **h** Statistical analysis for the frequency of OT-I donor cells that have undergone 1–4 divisions and those with more than 5 divisions. **i** Representative pseudocolor plot analysis of WT and SLP-76^2Cre-mE8^ OT-I donor cells for cleaved Caspase-3 (CC3) expression. Numbers indicate the frequency of cells in each quadrant. **j** Statistical analysis for CC3^+^ OT-I donor cells that have undergone more than 5 divisions. Symbols connected with a line indicate data from the same recipient mouse (**f**, **h**, **j**). Statistical analysis was carried out using two-way ANOVA (**b**, **h**) and Wilcoxon test (**f**, **j**). ns not significant.

First, we assessed the effect of the SLP-76^HA^ mutation on early activation of OT-I CD8^+^ T cells in response to OVA-derived peptides in vitro. CD8^+^ T cells isolated from WT CD45.1 OT-I and SLP-76^2Cre-mE8^ CD45.2 OT-I mice were mixed at a 1:1 ratio and co-cultured with T cell-depleted spleens (TdS) cells from (CD45.1 × CD45.2) F1 mice that had been loaded with WT OVA peptide N4 (SIINFEKL; ligand potency of 1) or 2 different OVA APLs with lower affinities for OT-I TCR, Q4H7 (SIIQFEHL; ligand potency of 1/167) and G4 (SIIGFEKL; ligand potency of 1/7515)^37^. Twenty hours later, activated OT-I CD8^+^ T cells were analyzed for expression of CD69 and PD-1 by flow cytometry (Supplementary Fig. 5e–h). When stimulated with N4 OVA peptide, SLP-76^2Cre-mE8^ OT-I cells expressed the same levels of CD69 (combined frequency of CD69^+^ PD-1^-^ and CD69^+^ PD-1^+^) as did WT OT-I cells at all concentrations of peptide (Fig. 5a left and Supplementary Fig. 5f). The dose of N4 peptide inducing a 50% maximum of CD69 expression (EC_50_) was comparable between WT and SLP-76^2Cre-mE8^ OT-I cells in three independent experiments (Fig. 5b and Supplementary Fig. 5i). In contrast, stimulation with Q4H7 led to a higher frequency of CD69 expression on SLP-76^2Cre-mE8^ OT-I cells at lower peptide concentrations ranging from 3.91 × 10^−10^ to 1.56 × 10^−9 ^M than that on WT OT-I cells (Fig. 5a middle and Supplementary Fig. 5g). Q4H7-stimulated SLP-76^2Cre-mE8^ OT-I cells showed a 2-fold decrease in the EC_50_ of CD69 (Fig. 5b and Supplementary Fig. 5i). Moreover, SLP-76^2Cre-mE8^ OT-I cells expressed more CD69 at any given concentrations of G4 (10^−8^–10^−5 ^M), but the EC_50_ of CD69 could not be determined because of poor activation of both WT and SLP-76^2Cre-mE8^ OT-I cells (Fig. 5a right and Supplementary Fig. 5h).

Next, we examined the effect of the SLP-76^HA^ mutation on phosphorylation of ERK MAP kinase (pERK) induced by TCR engagement. WT CD45.1 and SLP-76^2Cre-mE8^ CD45.2 OT-I CD8^+^ T cells mixed at a 1:1 ratio were co-cultured for up to 90 min with (CD45.1 x CD45.2) F1 TdS cells pre-loaded with 10^−11 ^M N4 peptide or 10^−9 ^M Q4H7 peptide, which induced the EC_50_ of CD69 (Fig. 5a, b). WT and SLP-76^2Cre-mE8^ OT-I cells interacted with N4-loaded TdS cells, as measured by CD45.2^+^ I-A^b+^ for WT OT-I and CD45.1^+^ I-A^b+^ for SLP-76^2Cre-mE8^ OT-I within CD3^+^ CD8^+^ YFP^+^ cells, with the same frequency and kinetics (Fig. 5c, d). Similarly, there was no difference between Q4H7-stimulated WT and SLP-76^2Cre-mE8^ OT-I cells in the frequency and kinetics of interaction with TdS cells (Fig. 5c, d). However, Q4H7 stimulation caused substantial diminution in the magnitude and delay in the kinetics of OT-I/TdS interaction compared to N4 stimulation (Fig. 5c, d). Notably, pERK was induced in OT-I cells that were in contact with peptide-loaded TdS cells but not in OT-I or TdS singlets (Supplementary Fig. 5j, k). When stimulated with N4 peptide, the level of pERK was comparable between WT and SLP-76^2Cre-mE8^ OT-I cells in contact with TdS at any time points (Fig. 5c, e). In contrast, Q4H7 stimulation led to a 2-fold increase in the level of pERK in SLP-76^2Cre-mE8^ OT-I cells interacting with TdS compared to WT OT-I cells as well as a marked delay in the kinetics of pERK induction in both WT and SLP-76^2Cre-mE8^ OT-I cells compared to N4 stimulation (Fig. 5c, e). These results indicate that the SLP-76^HA^ mutation does not affect the responsiveness of CD8^+^ T cells to a strong agonist peptide sufficient to induce robust T cell activation but renders them more responsive to weak agonists.

To examine the effect of the SLP-76^HA^ mutation on CD8^+^ T cell priming in vivo, 1:1-mixed WT CD45.1 and SLP-76^2Cre-mE8^ CD45.2 OT-I CD8^+^ T cells were labeled with CellTrace Violet (CTV) and transferred into (CD45.1 × CD45.2) F1 mice. One day later, recipient mice were subcutaneously immunized with Q4H7 APL emulsified in complete Freund’s adjuvant (CFA). We chose this APL because it led to enhanced responsiveness of SLP-76^2Cre-mE8^ OT-I cells in vitro compared with WT OT-I cells (Fig. 5a–e). Skin draining LNs (dLNs) were excised at 3 days post-immunization to analyze donor OT-I cells by flow cytometry (Supplementary Fig. 5l). The number of SLP-76^2Cre-mE8^ OT-I cells was slightly but significantly decreased, compared with WT OT-I cells from the same recipient mice (Fig. 5f and Supplementary Fig. 5m). However, the proportion of SLP-76^2Cre-mE8^ OT-I cells that had undergone more than 5 divisions increased (Fig. 5g, h). Indeed, highly dividing SLP-76^2Cre-mE8^ OT-I cells had a significant increase in the frequency of CC3^+^ cells than did WT OT-I cells (Fig. 5i, j). These results indicate that the SLP-76^HA^ mutation accelerates the proliferation of CD8^+^ T cells primed with a weak agonist in vivo but renders highly dividing CD8^+^ T cells more apoptotic, thus decreasing the number of antigen-specific CD8^+^ T cells during the priming phase.

### The SLP-76^HA^ mutation restrains the polarization of P14 CD8^+^ T cells toward T_CM_ cells in the memory phase upon acute LCMV infection

**Fig. 6:**
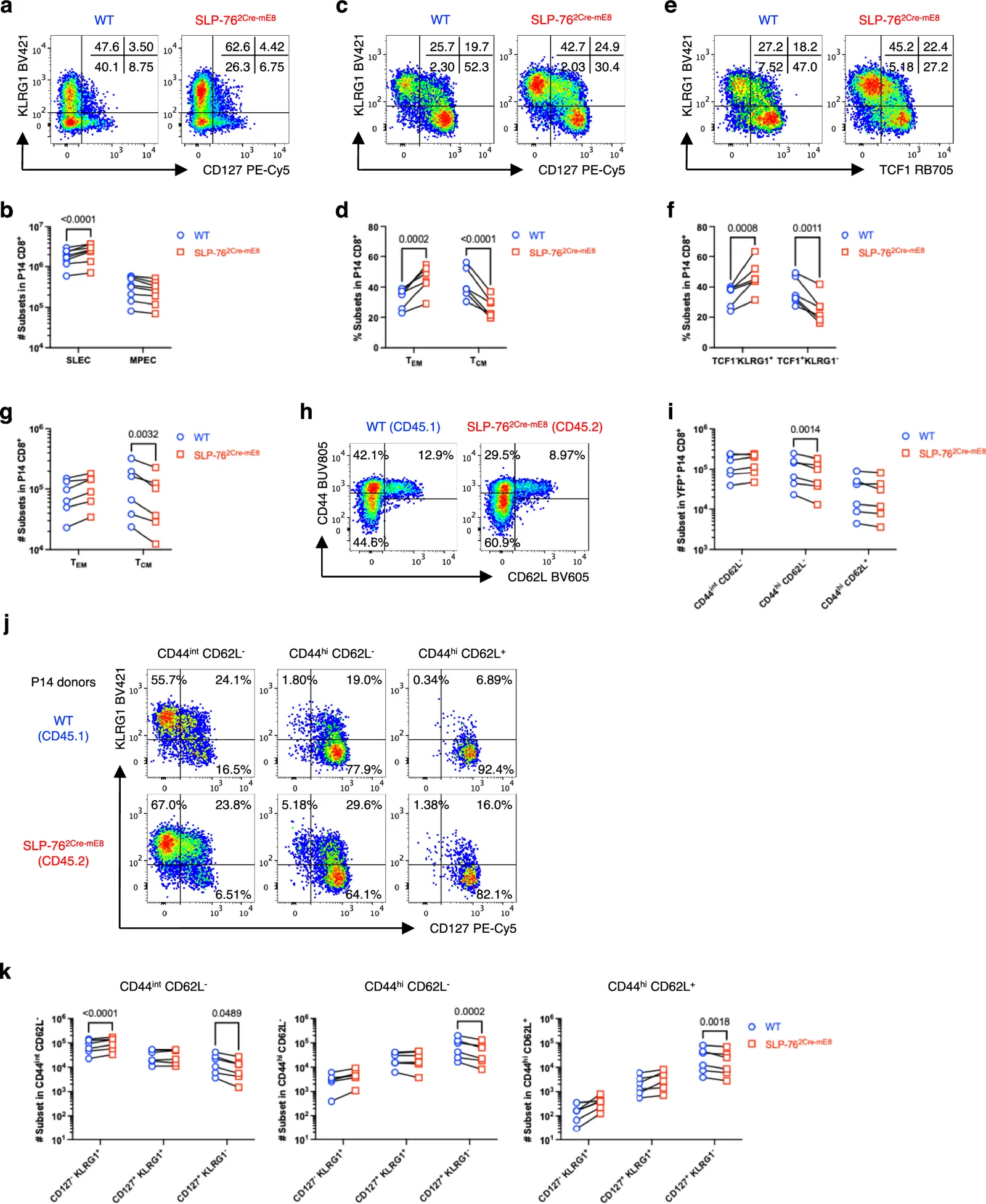
SLP-76^HA^ mutation restrains the polarization of P14 CD8^+^ T cells toward T_CM_ cells in the memory phase upon acute LCMV infection. WT CD45.1 and SLP-76^2Cre-mE8^ CD45.2 P14 CD8^+^ T cells were co-transferred into (CD45.1 x CD45.2) F1 mice. One day later, the recipients (*n* = 8 for 7 days and *n* = 6 for 28 days post-infection) were infected with LCMV_Arm_. **a**, **b** Seven days post-infection. **a** Representative pseudocolor plot analysis of WT and SLP-76^2Cre-mE8^ P14 donor cells for CD127 and KLRG1 expression. Numbers indicate the frequency of cells in each quadrant. **b** Statistical analysis for the number of SLEC (CD127^-^ KLRG1^+^) and MPEC (CD127^+^ KLRG1^-^) in P14 donor cells. **c**–**k** Twenty-eight days post-infection. **c** Representative pseudocolor plot analysis of WT and SLP-76^2Cre-mE8^ P14 donor cells for CD127 and KLRG1 expression. Numbers indicate the frequency of cells in each quadrant. **d** Statistical analysis for the frequency of T_EM_ (CD127^-^ KLRG1^+^) and T_CM_ (CD127^+^ KLRG1^-^) P14 donor cells. **e** Representative pseudocolor plot analysis of WT CD45.1 and SLP-76^2Cre-mE8^ P14 donor cells for TCF1 and KLRG1 expression. Numbers indicate the frequency of cells in each quadrant. **f** Statistical analysis for the frequency of TCF1^-^ KLRG1^+^ and TCF1^+^ KLRG1^-^ P14 donor cells. **g** Statistical analysis for the number of T_EM_ and T_CM_ P14 donor cells. **h** Representative pseudocolor plot analysis of WT and SLP-76^2Cre-mE8^ P14 donor cells for CD62L and CD44 expression. **i** Statistical analysis for the number of CD44^int^ CD62L^-^, CD44^hi^ CD62L^-^, and CD44^hi^ CD62L^+^ subpopulations. **j** Representative pseudocolor plot analysis for CD127 and KLRG1 expression in CD44^int^ CD62L^-^, CD44^hi^ CD62L^-^, and CD44^hi^ CD62L^+^ subpopulations of WT and SLP-76^2Cre-mE8^ P14 donor cells. **k** Statistical analysis for the number of CD127^-^ KLRG1^+^, CD127^+^ KLRG1^+^, and CD127^+^ KLRG1^-^ cells within CD44^int^ CD62L^-^ (left), CD44^hi^ CD62L^-^ (middle), and CD44^hi^ CD62L^+^ (right) subpopulations. Symbols connected with a line indicate data from the same recipient mouse. Statistical analysis was carried out using two-way ANOVA (**b**, **d**, **f**, **g**, **i**, **k**).

TCR signal strength has been demonstrated as one of the key determinants regulating a balance between effector memory (T_EM_) and central memory (T_CM_) CD8^+^ T cell generation in acute bacterial and viral infection models^39–44^. To assess the T cell-autonomous effect of the SLP-76^HA^ mutation on the T_EM_/T_CM_ balance, CD8^+^ T cells isolated from WT CD45.1 and SLP-76^2Cre-mE8^ CD45.2 mice on a P14 TCR transgenic background (specific for a glycoprotein of lymphocytic choriomeningitis virus; aa 33–41) were mixed at a 1:1 ratio and transferred into (CD45.1 × CD45.2) F1 mice^45^. One day later, recipient mice were infected with the Armstrong strain of lymphocytic choriomeningitis virus (LCMV_Arm_)^46^. YFP^+^ P14 donor cells in the spleen were analyzed at 7 and 28 days post-infection by flow cytometry. The SLP-76^HA^ mutation slightly enhanced the number of CD127^-^ KLRG1^+^ short-lived effector cells (SLEC) but had little effect on that of CD127^+^ KLRG1^-^ memory-precursor effector cells (MPEC) at 7 days post-infection (Fig. 6a, b). At 28 days post-infection, the number of SLP-76^2Cre-mE8^ P14 cells was equivalent to that of co-transferred WT P14 cells (Supplementary Fig. 6a, b). Notably, SLP-76^2Cre-mE8^ P14 cells showed a marked decrease in the frequency of CD127^+^ KLRG1^-^ T_CM_ cells with a concomitant increase in that of CD127^-^ KLRG1^+^ T_EM_ cells (Fig. 6c, d). Consistent with these results, the frequency of a TCF1^+^ KLRG1^-^ population corresponding to stem-like memory T cells was markedly decreased in SLP-76^2Cre-mE8^ P14 donor cells^47^ (Fig. 6e, f). The number of SLP-76^2Cre-mE8^ P14 T_EM_ cells was slightly increased by 1.3-fold, but this increase was not statistically significant. In contrast, there was a 2-fold decrease in the number of SLP-76^2Cre-mE8^ P14 T_CM_ cells (Fig. 6g and Supplementary Fig. 6c). These results indicate that the SLP-76^HA^ mutation does not affect the skewing toward MPEC in the expansion phase but hinders the generation of T_CM_ cells in the early memory phase in a T cell-autonomous manner. The same trend was evident when splenic CD8^+^ T cells bound to LCMV_gp33-41_/H-2D^b^ tetramer (gp33-Tet) were analyzed for T_EM_ and T_CM_ cells in SLP-76^2Cre-mE8^ mice bearing endogenous TCR repertoire that had been infected with LCMV_Arm_ for 30 days (Supplementary Fig. 7c, d). However, the numbers of gp33-Tet^+^ T_EM_ and T_CM_ cells were not significantly different between WT and SLP-76^2Cre-mE8^ mice because of a large variation in the robustness of gp33-Tet^+^ CD8^+^ T cell responsiveness in both groups (Supplementary Fig. 7a, b, e).

We further assessed the expression of CD44 and CD62L on WT CD45.1 and SLP-76^2Cre-mE8^ CD45.2 P14 donor cells at 28 days post-infection with LCMV_Arm_. Both donor cells developed into three distinct populations: CD44^int^ CD62L^-^, CD44^hi^ CD62L^-^, and CD44^hi^ CD62L^+^ (Fig. 6h). The SLP-76^HA^ mutation did not affect the absolute number of CD44^int^ CD62L^-^ and CD44^hi^ CD62L^+^ cells, except for a slight reduction in CD44^hi^ CD62L^-^ cells (Fig. 6i). When these three subpopulations were analyzed for CD127 and KLRG1 expression, a large proportion of CD44^int^ CD62L^-^ cells differentiated into CD127^-^ KLRG1^+^ T_EM_ cells, and the SLP-76^HA^ mutation significantly increased the number of T_EM_ cells (Fig. 6j, k). In contrast, both CD44^hi^ CD62L^-^ and CD44^hi^ CD62L^+^ cells predominantly skewed toward CD127^+^ KLRG1^-^ T_CM_ cells, and the SLP-76^HA^ mutation significantly decreased the number of T_CM_ cells (Fig. 6j, k). Similarly, we analyzed the CD44^int^ CD62L^-^, CD44^hi^ CD62L^-^, and CD44^hi^ CD62L^+^ subpopulations for TCF1 and KLRG1 expression. Consistent with the T_EM_/T_CM_ analysis shown in Fig. 6h–k, the CD44^int^ CD62L^-^ subpopulation mostly differentiated into TCF1^-^ KLRG1^+^ cells, and the SLP-76^HA^ mutation promoted this differentiation (Supplementary Fig. 6d, e). However, both the CD44^hi^ CD62L^-^ and CD44^hi^ CD62L^+^ subpopulations preferentially developed into TCF1^+^ KLRG1^-^ stem-like memory T cells, thus the SLP-76^HA^ mutation reduced the polarization toward stem-like memory cells (Supplementary Fig. 6d, e). In summary, the SLP-76^HA^ mutation influences the differentiation state of LCMV-specific CD8^+^ T cells in the memory phase with a minimal impact on CD44 and CD62L expression.

### The SLP-76^HA^ mutation leads to the diminution in mature Tfh cells and impairs germinal center reaction

**Fig. 7:**
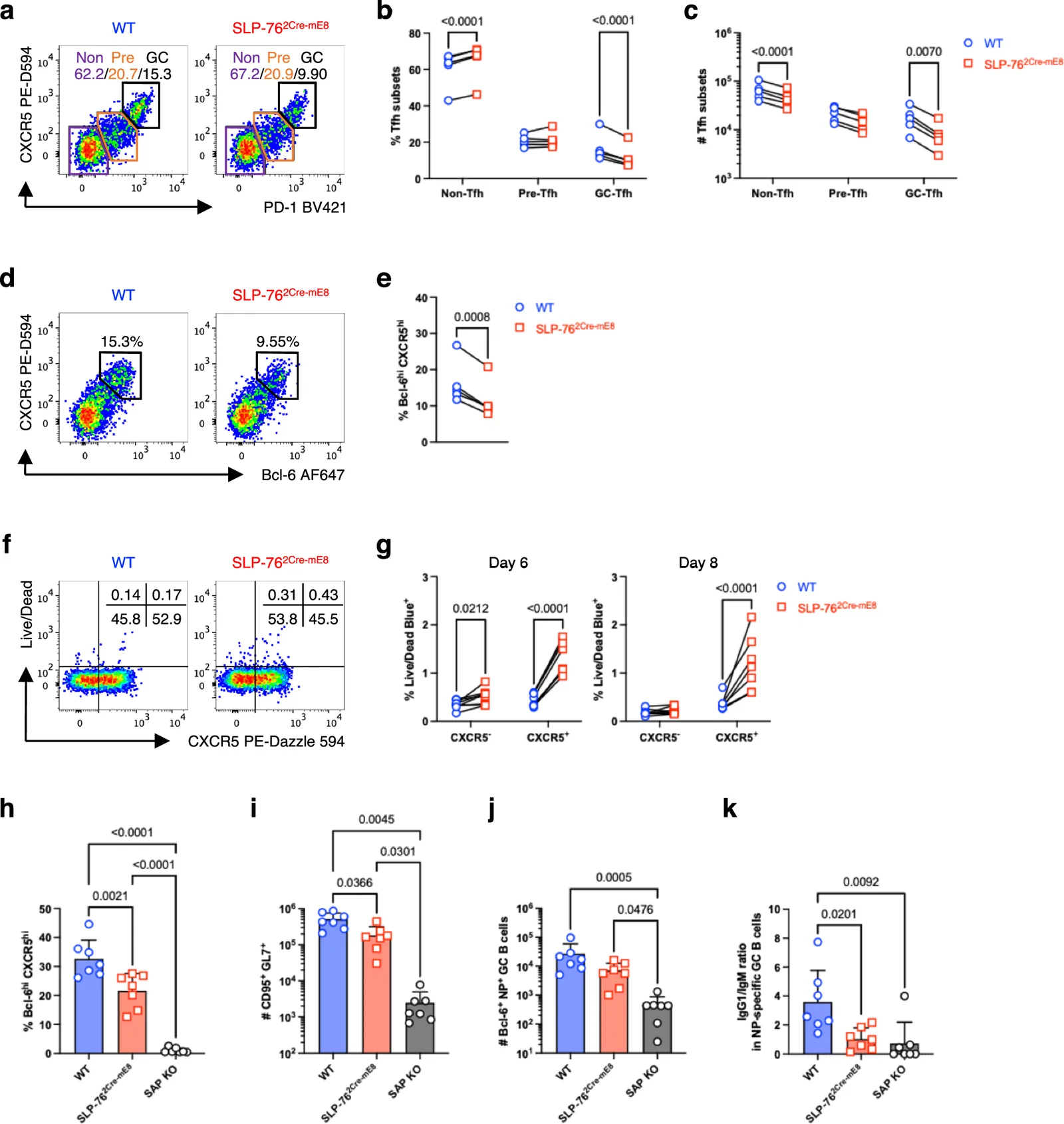
SLP-76^HA^ mutation decreases the number of mature Tfh cells and impairs germinal center reaction. **a**–**g** WT CD45.1 and SLP-76^2Cre-mE8^ CD45.2 OT-II CD4^+^ T cells were co-transferred into (CD45.1 × CD45.2) F1 mice. One day later, recipient mice (*n* = 8 for 6 days, *n* = 7 for 8 days, and *n* = 5 for 10 days post-immunization) were subcutaneously immunized with EndoFit OVA in CFA. **a** Representative pseudocolor plot analysis of WT and SLP-76^2Cre-mE8^ OT-II donor cells for PD-1 and CXCR5 expression at 10 days post-immunization. Numbers indicate the frequency of cells in each population. Statistical analysis for the frequency (**b**) and number (**c**) of non-Tfh (PD-1^-^ CXCR5^-^), pre-Tfh (PD-1^int^ CXCR5^int^), and mature (GC) Tfh (PD-1^hi^ CXCR5^hi^) OT-II donor cells. **d** Representative pseudocolor plot analysis of WT and SLP-76^2Cre-mE8^ OT-II donor cells for Bcl-6 and CXCR5 expression. **e** Statistical analysis for the frequency of Bcl-6^hi^ CXCR5^hi^ OT-II donor cells. **f** Representative pseudocolor plot analysis of WT and SLP-76^2Cre-mE8^ OT-II donor cells for CXCR5 and Live/Dead Blue staining at 6 days post-immunization. Numbers indicate the frequency of cells in each population. **g** Statistical analysis for the frequency of dead cells in CXCR5^-^ and CXCR5^+^ populations at 6 and 8 days post-immunization. **h**–**k** WT, SLP-76^2Cre-mE8^, or SAP KO OT-II CD4^+^ T cells (all CD45.2) were transferred into SAP KO CD45.1 mice. One day later, recipient mice (*n* = 7 for each group) were subcutaneously immunized with NP-OVA in CFA. Statistical analysis for the frequency of Bcl-6^hi^ CXCR5^hi^ OT-II donor cells (**h**), the number of total (**i**) and NP-specific (**j**) GC B cells, and IgG1/IgM ratio in NP-specific GC B cells (**k**) at 10 days post-immunization. Symbols connected with a line indicate data from the same recipient mouse (**b**, **c**, **e**, **g**). Data are shown as mean ± SD (**h**–**k**). Statistical analysis was carried out using two-way ANOVA (**b**, **c**, **g**), Wilcoxon test (**e**), and Kruskal-Wallis test (**h**–**k**).

T follicular helper (Tfh) cells are a subset of CD4^+^ helper T (Th) cells that supports humoral immune responses to T-dependent antigens by B cells in the germinal center (GC)^48^. As TCR signal strength has been proposed to play a crucial role in the fate decision of naïve CD4^+^ T cells toward either Tfh cells or non-Tfh cells^49,50^, we assessed the T cell-autonomous effect of the SLP-76^HA^ mutation on Tfh differentiation. CD4^+^ T cells isolated from WT CD45.1 and SLP-76^2Cre-mE8^ CD45.2 mice on an OT-II TCR transgenic background (specific for OVA_323–339_ peptide) were mixed at a 1:1 ratio and transferred into (CD45.1 × CD45.2) F1 mice^51^. One day later, recipient mice were immunized with OVA protein in CFA. YFP^+^ OT-II donor cells in skin dLNs were analyzed by flow cytometry at 10 days post-immunization (Supplementary Fig. 8a). SLP-76^2Cre-mE8^ OT-II cells showed a slight but significant decrease in number by 1.6-fold, compared with WT OT-II cells from the same recipient mice (Supplementary Fig. 8b). Notably, the frequencies of PD-1^high^ CXCR5^high^ and Bcl-6^high^ CXCR5^high^ mature Tfh (GC-Tfh) cells were selectively decreased, whereas those of PD-1^-^CXCR5^-^ non-Tfh cells and PD-1^int^ CXCR5^int^ pre-Tfh cells was either slightly increased or unchanged^48^ (Fig. 7a, b). Neither WT nor SLP-76^2Cre-mE8^ OT-II donor cells (that were all YFP^+^ CD44^high^) differentiated into Foxp3^+^ CXCR5^+^ T follicular regulatory (Tfr) cells, whereas CD44^high^ CD4^+^ T cells derived from the recipient mice had a small but distinct population of Tfr cells (Supplementary Fig. 8d, e). The numbers of SLP-76^2Cre-mE8^ non-Tfh and pre-Tfh cells were slightly decreased to a degree equivalent to that of total SLP-76^2Cre-mE8^ OT-II donor cells, whereas SLP-76^2Cre-mE8^ mature Tfh cells were markedly decreased by 2.5-fold (Fig. 7c and Supplementary Fig. 8c). Consistent with these results, the frequency of dead cells in a CXCR5^+^ fraction was substantially enhanced in SLP-76^2Cre-mE8^ OT-II donor cells at 6 and 8 days post-immunization (Fig. 7f, g and Supplementary Fig. 8f). These results indicate that the SLP-76^HA^ mutation does not prevent antigen-primed CD4^+^ T cells from the fate decision toward Tfh cells but instead leads to a mild decrease in the number of clonally expanded CD4^+^ T cells. Importantly, the SLP-76^HA^ mutation results in the failure to maintain mature Tfh cells, presumably because the enhanced TCR signal strength caused by this mutation may promote death of pre-Tfh cells during cognate interaction with B cells.

To assess whether the decreased mature Tfh cells by the SLP-76^HA^ mutation would influence GC reaction, CD4^+^ T cells isolated from WT or SLP-76^2Cre-mE8^ CD45.2 OT-II mice were transferred into CD45.1 mice deficient in SLAM-associated protein (SAP). SAP deficiency severely impairs humoral immune response due to the failure to generate mature Tfh cells, and reconstitution with SAP sufficient CD4^+^ T cells can restore GC reaction in SAP KO mice^52,53^. One day after OT-II cell transfer, recipient SAP KO mice were immunized with NP-OVA (4-hydroxy-3-nitrophenylacetyl conjugated to OVA) in CFA. SAP KO CD45.1 mice receiving SAP KO CD45.2 OT-II cells were used as a negative control. Ten days after immunization, donor CD45.2 OT-II cells and recipient SAP KO CD45.1 B cells were analyzed for development into Tfh cells and GC B cells, respectively. YFP^+^ SLP-76^2Cre-mE8^ OT-II donor cells had a decreased frequency of Bcl-6^high^ CXCR5^high^ mature Tfh cells compared to YFP^+^ WT OT-II donor cells (Fig. 7h and Supplementary Fig. 8g, h). Consistent with these results, the numbers of total GC B cells (CD45.1^+^ CD19^+^ CD3^-^ CD38^-^ IgD^-^ Blimp-1^-^ CD138^-^ CD95^+^ GL7^+^) and NP-specific (Bcl-6^+^ NP^+^) GC B cells in SLP-76^2Cre-mE8^ OT-II donor group were decreased by 2.8-fold and 3.8-fold, respectively, compared to those in WT OT-II donor group (Fig. 7i, j and Supplementary Fig. 8g, i, j). However, due to a high variation of the frequency of an NP^+^ population within GC B cells, the difference in the number of NP-specific GC B cells between WT and SLP-76^2Cre-mE8^ OT-II donor groups was not statistically significant (*p* = 0.5043 by Kruskal-Wallis’ test). Nonetheless, NP-specific GC B cells in the SLP-76^2Cre-mE8^ OT-II donor group showed impaired antibody class switching, as evident by a significantly lower IgG1/IgM ratio than that in the WT OT-II donor group (Fig. 7k and Supplementary Fig. 8k). As expected, SAP KO OT-II donor cells failed to generate mature Tfh cells or induce GC reaction in SAP KO recipients (Fig. 7h–k and Supplementary Fig. 8g–k). These results indicate that the SLP-76^HA^ mutation in CD4^+^ T cells impairs the induction of Tfh-mediated GC reaction.

Taken together, the affinity of interaction between the SLP-76 PRR_185–200_ and the PLC-γ1 SH3 domain, which defines the level of PLC-γ1 activation induced upon TCR engagement, has a critical role in fine-tuning TCR signal strength in multiple settings. The native sequence and the low affinity of the interaction prevent the excessive death of thymocytes during positive and negative selection, restrains over-activation of CD8^+^ T cells leading to enhanced apoptosis during the priming period, limits the polarization toward CD8^+^ T_EM_ cells upon acute virus infection, and maintains normal levels of mature Tfh cells.

## Discussion

Activation of phospholipases is central to induction of many cellular changes in eukaryotic systems including proliferation, growth and migration. One of the multiple isoforms of phospholipase, PLC-γ1, is activated during the process of lymphocyte activation. This form of phospholipase C contains three regulatory domains, N-terminus SH2 (nSH2), C-terminus SH2 (cSH2), and SH3, that are important for its recruitment and activation^54,55^. In T lymphocytes, antigen-induced activation through the TCR leads to phosphorylation of the LAT adapter molecule at its four distal tyrosine residues^1,4^. The nSH2 domain of PLC-γ1 binds to phosphorylated Y132 of LAT. Many other adapter and signaling proteins bind to the three distal LAT phosphorylated tyrosine residues including a cytosolic dimer comprised of Gads and SLP-76. The proximity of PLC-γ1 and the Gads/SLP-76 dimer all bound to a LAT molecule enables formation of a tetrameric structure (LAT/Gads/SLP-76/PLC-γ1) in which the PLC-γ1 SH3 domain is able to bind to the SLP-76 PRR^2^.

Our laboratory subsequently identified the affinities of the domain interactions within this tetramer^5^. The LAT/Gads, Gads/SLP-76 and LAT/PLC-γ1 interactions are easily determined by ITC at room temperature. In contrast, the low affinity SLP-76/PLC-γ1 interaction can only be determined at 4 °C. We then measured PLC-γ1 activation in an in vitro system using liposomes containing the PLC-γ1 substrate PIP2 and demonstrated that formation of the LAT/Gads/SLP-76/PLC-γ1 tetramer at the membrane results in the maximal induction of PLC-γ1 hydrolysis as measured by IP3 production^6^. This functional data in conjunction with our previous biophysical studies confirmed the importance of the SLP-76/PLC-γ1 interaction leading to circular tetramer formation. We proposed that formation of the circular tetramer would induce conformational changes in PLC-γ1 leading to its activation.

In the present study we first compared the amino acid sequences of SLP-76 and PLC-γ1 from multiple vertebrates to further test the significance of the low affinity SLP-76/PLC-γ1 interaction within the tetrameric complex. We confirmed that there was a high degree of sequence conservation in the two critical sites of interaction, the SLP-76 PRR_185–200_ and the PLC-γ1 SH3 domain, from human to fishes. These data suggest that the conserved low affinity interaction between these protein domains would play a critical role in modulating the circular tetrameric complex between open and closed conformations to optimize PLC-γ1 activation.

To test this hypothesis, we generated a set of recombinant SLP-76 proteins with the wild-type PRR_185–200_ sequence and with the SLP-76^NA^ and SLP-76^HA^ mutations predicted to lose and enhance the capacity to interact with PLC-γ1 via its SH3 domain, respectively. As predicted, our ITC analysis revealed that substitution of the native SLP-76 protein with the SLP-76^HA^ mutant resulted in enhanced binding to PLC-γ1, and that the SLP-76^NA^ mutant was unable to interact with PLC-γ1. We then performed the liposome-based assay to evaluate the effect of these sequence changes on PLC-γ1 activity. We found that incubation of the liposomes with the LAT/Gads/SLP-76^HA^/PLC-γ1 tetramer led to a small but significant increase in IP3 production compared to that with the LAT/Gads/SLP-76^WT^/PLC-γ1 tetramer. In contrast, formation of the tetramer containing the SLP-76^NA^ resulted in significantly lower levels of IP3 production than that with the SLP-76^WT^. These results indicate that the SLP-76/PLC-γ1 interaction is essential for regulation of the level of PLC-γ1 activation via formation of the closed circular tetramer.

The SLP-76 PRR_185–194_, identified as the minimal binding site for the PLC-γ1 SH3 domain, has also been reported to associate with the SH3 domain of Lck and Itk in vitro and in vivo^8–13^. These observations raise the possibility that the active SLP-76 PRR_185–200_ mutation used in our study might enhance the association of Lck and/or Itk within the TCR-induced multi-protein complex, thus increasing the activity of PLC-γ1 and the resultant TCR signal strength. However, this possibility can be excluded by the following pieces of evidence. First, sequence alignment of the Itk and Lck SH3 domains revealed that both lacked two of the four amino acids critical for binding to the SLP-76 PRR, which are present in the PLC-γ1 SH3 domain^8^. Second, our ITC analysis revealed no measurable interaction of Itk with any of the three forms of SLP-76 PRR_185–200_ peptides. Third, neither Lck nor Itk co-immunoprecipitates with SLP-76 in carefully controlled experiments using Lck-deficient J.CaM1 cells as a negative control, as demonstrated by Yablonski et al.^2^. Fourth, the SLP-76 PRR_180–207_ did not have any sequence feature similar to the PIPRSP motif in LAT_54–90_ that associates with the Lck SH3 domain^14^.

Although interaction of the Itk SH3 domain with the SLP-76 PRR is unlikely to occur, Itk binds to phosphorylated SLP-76 at Y145 via its SH2 domain with the adapter Nck and the enzyme Vav, thereby forming a heptameric signaling complex^56–58^. Activated Itk in turn phosphorylates SLP-76 at Y173, which primes PLC-γ1 for Itk-mediated phosphorylation of a regulatory tyrosine residue Y783^59,60^. Structural studies have demonstrated how this phosphorylation event would result in conformation changes in PLC-γ1 critical for its activation^54,55^. We speculate that the closed circular tetrameric formation mediated by the SLP-76/PLC-γ1 interaction would regulate the frequency and/or duration of SLP-76-bound Itk recruitment to the PLC-γ1 regulatory domain. Moreover, we previously demonstrated that replacement of PLC-γ1 in the tetramer by PLC-γ1 that have been phosphorylated at Y783 maximizes PLC-γ1 hydrolysis^6^. We thus propose that formation of the closed circular tetramer and phosphorylation of PLC-γ1 at Y783 co-operate to induce conformational changes necessary to optimize enzyme activation.

Replacement of SLP-76^WT^ with the SLP-76^HA^ protein led to a decrease in the number of post-positive selection DP3 thymocytes and post-negative selection medullary CD4 or CD8 SP most mature M2 thymocytes, accompanied by increased apoptosis. These results imply that the weak SLP-76/PLC-γ1 interaction acts as a checkpoint for αβ T cell development. However, the degree of such decreases was very modest compared with that observed in LAT^G135D^ mice, suggesting that the SLP-76^HA^ mutation used in our study is less effective in enhancing TCR signal strength than the LAT^G135D^ mutation which accelerates the kinetics and degree of LAT phosphorylation at Y136^44^. We also noted that SLP-76^4Cre-mE8^ mice had a slight but significant increase in the number of memory phenotype (T_MP_) CD8^+^ T cells in the periphery. This phenotype was further enhanced when these mice were bred onto OT-I TCR transgenic mice that have a relatively high affinity for self-ligands. Notably, none of SLP-76^4Cre-mE8^ mice showed any signs of autoimmunity as monitored for 1.5 years, whereas LAT^G135D^ mice spontaneously develop autoimmune diseases at 1 year of age^44^. This difference is accompanied by a marked increase in dysfunctional Treg cells by the LAT^G135D^ mutation that fail to suppress the activation of CD4^+^ and CD8^+^ T_MP_ cells, thus breaking peripheral tolerance^44^. In contrast, the SLP-76^HA^ mutation barely affected the homeostasis and functional property of the Treg compartment, which would be capable of preventing the aberrant activation of autoreactive T_MP_ cells in SLP-76^4Cre-mE8^ mice. As opposed to αβ T cell development, the SLP-76^HA^ mutation promoted the development of agonist-selected iNKT cells and Type B IELp, indicating that the weak SLP-76/PLC-γ1 interaction limits the selection of these innate-like T cell lineages. Whether and/or how the SLP-76^HA^ mutation would alter the homeostasis and functional properties of these T cells in the secondary lymphoid organs and non-immune tissues requires further investigation.

We employed TCR transgenic systems to evaluate the effect of the SLP-76^HA^ mutation on the responsiveness of antigen-specific T cells. We showed that the SLP-76^HA^ mutation rendered OT-I CD8^+^ T cells more responsive to OVA APLs with lower affinities but not to the WT N4 peptide in vitro, as measured by TCR-induced pERK and CD69 expression. However, the degree of enhanced TCR signal strength by the SLP-76^HA^ mutation was much smaller than that induced by the CD3ζ 6 F(i) or LAT^G135D^ mutations, as judged by the EC_50_ of CD69 expression on OT-I cells stimulated with Q4H7 or an equivalent OVA APL^44,61^. The latter two mutations lead to a substantial increase by over 10-fold and 100-fold, respectively, whereas the SLP-76^HA^ mutation resulted in only a 2-fold increase. Nonetheless, our observations that the SLP-76^HA^ mutation accelerated the division of donor OT-I cells in response to Q4H7 but led to increased apoptosis in those cells in vivo highlight the biological relevance of the weak SLP-76/PLC-γ1 interaction in optimizing the responsiveness of peripheral T cells by fine-tuning TCR signal strength.

The potency of TCR signaling has been demonstrated as a key determinant underlying the fate decision of CD8^+^ T cells toward T_CM_ and T_EM_ cells^39–44^. Using a P14 TCR transgenic and endogenous TCR systems, we showed that the SLP-76^HA^ mutation markedly reduced the generation of T_CM_ cells in the memory phase upon acute LCMV_Arm_ infection but had very little effect on the bifurcation into MPEC in the expansion phase. In contrast, the LAT^G135D^ mutation restrained OT-I cells from skewing toward MPEC at 7 days post-infection with *Listeria monocytogenes* expressing a WT OVA peptide or a low affinity V4 OVA APL^44^. These results indicate that the SLP-76^HA^ mutation is less potent than the LAT^G135D^ mutation at enhancing TCR signal strength in LCMV-reactive CD8^+^ T cells during the expansion phase when the virus-derived antigens are abundantly present. Notably, dampening TCR signal strength caused by the conditional SLP-76 deletion during the contraction phase has been demonstrated to enhance LCMV-specific T_CM_ cells in the memory phase^40^. This leads us to speculate that the SLP-76^HA^ mutation would enhance TCR signal strength in LCMV_Arm_-primed CD8^+^ T cells that recognize limited amounts of LCMV-derived peptide/MHC I complexes during the contraction phase, thus restraining the polarization toward T_CM_ cells.

Higher potency of TCR-mediated signaling has been demonstrated to favor the fate decision of naïve CD4^+^ T cells toward Tfh cells over other effector Th subsets^49,50^. Using an OT-II CD4^+^ T cell adoptive transfer system, we showed that the SLP-76^HA^ mutation did not alter the frequency of pre-Tfh cells, indicating that the degree of enhanced TCR signal strength by the SLP-76^HA^ mutation is insufficient to force antigen-primed OT-II cells to undertake the Tfh differentiation initiation program^48^. Full polarization into mature Tfh cells requires a stable and prolonged interaction of pre-Tfh cells with B cells presenting a cognate antigen, where pre-Tfh cells tend to receive stronger TCR-mediated signaling^48^. Indeed, the SLP-76^HA^ mutation decreased the number of mature Tfh cells, which was accompanied by increased death of CXCR5^+^ OT-II donor cells. Thus, the SLP-76^HA^ mutation might elevate TCR signaling to a level that triggers the death of Tfh cells upon recognition of a cognate antigen presented by B cells, thereby significantly reducing B cell activation and limiting their maturation into GC B cells. We noted that NP-OVA-immunized SAP KO mice that had received SLP-76^2Cre-mE8^ OT-II cells showed a 3-fold decrease in the frequency of CD38^-^ IgD^-^ activated B cells, which largely contributed to the impaired generation of GC B cells. While SLP-76^HA^ mature Tfh cells could induce GC B cell development to a lesser extent, the resulting GC B cells exhibited severely impaired immunoglobulin class switching, indicating that the helper function of SLP-76^HA^ mature Tfh cells is compromised. We ruled out the possibility that the impaired GC reaction observed in the SLP-76^2Cre-mE8^ OT-II donor group was a consequence of enhanced generation of Tfr cells. However, we could not determine whether the SLP-76^HA^ mutation might not affect Tfr differentiation or whether the OVA/CFA immunization system might not be optimal for inducing Tfr cells. Further investigation is required to address the mechanisms underlying the death and dysfunction of Tfh cells mediated by the SLP-76^HA^ mutation.

There has been much attention recently to the central role of LAT phosphorylation on the Y132 residue in determining the response of TCR signaling^44,62^. The kinetics of phosphorylation at this site, as regulated by the identity of the preceding glycine residue, are slower than the phosphorylation of the other three phosphorylated LAT residues. This slower phosphorylation acts as a proof-reading step necessary for defining the ultimate response by the T cell. Moreover, six tyrosine residues on immunoreceptor tyrosine-based activation motifs within the CD3ζ chains play a critical role in ligand discrimination by the TCR recognizing antigenic peptides with lower affinities in the context of MHC^61^. Our observations using the SLP-76^HA^ mutant shed light on the importance of the SLP-76 PRR_185–200_ in optimizing the activation of T cells. As the LAT/Gads/SLP-76/PLC-γ1 tetramer physiologically forms by the cooperative interaction of the four proteins following engagement of the TCR/CD3 complex and LAT phosphorylation, the SLP-76/PLC-γ1 interaction would serve as an additional regulatory kinetic proof-reading mechanism determining optimal TCR signaling.

Our present study, combining biophysical analyses, biochemical reconstitution, and use of a conditional knock-in mouse, indicate that it is the formation of the LAT/Gads/SLP-76/PLC-γ1 circular tetramer that is central to appropriate PLC-γ1 activation. Recruitment of PLC-γ1 to phosphorylated LAT associated with Gads and the SLP-76^NA^ mutant, which is unable to interact with PLC-γ1, was insufficient for full PLC-γ1 activation. Use of the SLP-76^HA^ mutant enhancing the affinity for PLC-γ1 by 2–3-fold over SLP-76^WT^ led to a slight but significant increase in PLC-γ1 activity. However, this small increase in the affinity of the SLP-76/PLC-γ1 interaction resulted in impaired thymocyte development and peripheral T cell responses in vivo. These results and the fact that the amino acid sequences of the relevant interacting sites of PLC-γ1 and SLP-76 are conserved lead to the conclusion that the affinities of protein-protein interaction defined in the tetramer formation, especially the weak SLP-76/PLC-γ1 interaction, are critical to the controlled activation of PLC-γ1. This weak interaction thus fine-tunes TCR signal strength to generate appropriate T cell-mediated immune responses for all vertebrates.

## Methods

### Protein purification

The cDNA fragments of Halo-tagged PLC-γ1 and dual-tagged (His_6_- and Halo-) Gads were inserted into Gateway pDest vectors (Thermo Fisher Scientific). Each protein was expressed in Sf9 cells in Sf-900 III SFM (Thermo Fisher Scientific) and harvested (Leidos Biomedical Research, Inc.). The protein lysates were prepared by French press and centrifugation. HaloLink Resin (Promega) and a HisTrap HP column (Cytiva) were used for PLC-γ1 and Gads purification, respectively. The HaloTag on PLC-γ1 was cleaved away by TEV protease (Leidos Biomedical Research, Inc.), but the HaloTag on Gads remained to enhance protein solubility. After the affinity purifications, these proteins were further purified by size-exclusion chromatography (SEC) using Superdex 200 Increase SEC column, 10/300 GL (Cytiva).

The SLP-76 cDNA (103-258aa with Y113F and Y173F mutation) linked to a His_6_-GST-TEV protease cleavage sequence at N-terminus was inserted into pET28(+) vector (Novagen). The protein was expressed in Rosetta 2(DE3)pLysS Competent Cells (Novagen). The protein lysate prepared by French press and centrifugation was incubated with Glutathione Resin (GenScript) for 2 h at 4 °C. After washing the resin, the SLP-76 protein was cleaved off from the resin with TEV protease. The liberated SLP-76 protein and the His_6_-TEV protease were separated by Ni-affinity chromatography using a HisTrap HP column with a shallow imidazole gradient. The eluted SLP-76 protein was further purified by SEC.

The cDNA fragment of LAT (48-233aa) linked to His_6_- and MBP-tag sequences at the N-terminus was inserted into pET28(+) vector. The protein was expressed in Rosetta2(DE3)pLysS cells. Multi-tagged LAT protein was purified from the protein lysate using a HisTrap HP column and a RESOURCE Q anion exchange chromatography (AEX) column (Cytiva). The His_6_- and MBP-tag at the N-terminus were removed using TEV protease. The cleaved LAT protein was purified by the same AEX column again and then treated with in vitro phosphorylation at tyrosines 132 and 171. Final AEX and SEC were performed to purify the phosphorylated LAT, and phosphorylation was validated by mass spectrometry.

The bacmid encoding full-length mouse ITK with C96E/T110I mutations (His_6_-3C-Halo-tev-ITK(C96E/T110I)-FLAG) was created as previously described^63^. Protein expression was carried out in Tni-FNL insect cells following previously established protocol (Leidos Biomedical Research, Inc.)^64,65^. The cells were lysed by passing through a microfluidizer and the lysate was clarified by centrifugation. The tagged protein was purified by Ni-affinity chromatography with an increasing imidazole gradient. The fractions containing ITK were pooled and directly analyzed by SEC without further concentration or freezing to obtain the maximum amount of monomeric protein. After SEC, the fractions containing oligomerized and monomeric protein were separated and the monomeric ITK sample was used in analysis.

### Enzymatic assay for PIP2 cleavage by PLC-γ1

The detailed experimental procedures were described in Wada et al.^6^. Large Unilamellar Vesicles (LUVs) provided lipid bilayer surfaces as a surrogate of the cellular membrane environment and also contained PIP2, a substrate for the PLC-γ1 enzymatic reaction. The LUVs were prepared using a Mini extruder kit (Avanti Polar Lipids) and comprised of phosphatidylcholine (PC), phosphatidylethanolamine (PE), phosphatidylserine (PS), phosphatidylinositol 4,5-bisphosphate (PIP2), cholesterol, and 1,2-dioleoyl-sn-glycero-3-[(N-(5-amino-1-carboxypentyl) iminodiacetic acid) succinyl]; DGS-NTA(Ni). At the enzymatic reaction step, the proteins were mixed and incubated at 30˚C, and the reaction was initiated by adding the LUVs. The diameter of the LUV was 0.4 µm, and protein concentrations were 75 nM of PLC-γ1, 375 nM of LAT, 562 nM of Gads and SLP-76. The reaction was stopped by flash freezing in liquid nitrogen. After removing the LUVs and proteins through a Captiva EMR Lipid (Agilent) and a Macro Spin Column C18 (Harvard Apparatus), the IP3 in the elution was quantified by HPLC analysis as described in Mayr et al.^66,67^. Quantification was performed using Image J.

### Isothermal titration calorimetry for measuring the affinity of protein interaction

The reference experimental procedures were described in Manna et al.^3^ and Houtman et al.^5^. ITC measurements were performed using a VP-ITC calorimeter or PEAQ-ITC (Malvern Panalytical). Titrations were performed in 20 mM Hepes (pH 7.4), 100 mM NaCl, with 0.1 mM DTT at 5˚C. The initial protein concentration of SLP-76 in the cell and that of PLC-γ1 or Itk in the syringe were 4 µM and 40 µM, respectively. The injections of 8 µL were repeated 29 times in VP-ITC. The injections of 2 µL were repeated 19 times in PEAQ-ITC. Heat changes in each injection were fitted with the single binding site mechanism with 1:1 stoichiometry. The values for the stoichiometry of binding and thermodynamic constants were determined using the software package provided by Malvern Panalytical.

### Sequence alignment analysis of proline-rich region in SLP-76 and SH3 domain in PLC-γ1

Nineteen sequences of SLP-76 orthologs in jawed vertebrates from cartilaginous fish to primates, were selected. The sequences were searched by UniProt and Ensembl database, and the alignment analysis was performed using Protein BLAST.

### Mice

All mice used in the present study were on a C57BL/6 background. C57BL/6 J (B6/J) mice (Strain #: 000664), B6 CD45.1 mice (B6.SJL-*Ptprc*^*a*^*Pepc*^*b*^/BoyJ; Strain #: 002014)^38^, CD4^Cre^ mice (B6.Cg-Tg(Cd4-cre)1Cwi/BfluJ; Strain #: 022071)^16^, pLck^Cre^ mice (B6.Cg-Tg(Lck-cre)548Jxm/J, Strain #: 003802)^27^, OT-I mice (C57BL/6-Tg(TcraTcrb)1100Mjb/J; Strain #: 003831)^36^, OT-II mice (B6.Cg-Tg(TcraTcrb)425Cbn/J; Strain #: 004194)^51^, and Rosa26^LSL-eYFP^ mice (B6.129×1-*Gt(ROSA)26Sor*^*tm1(EYFP)Cos*^/J; Strain #: 006148)^18^ were purchased from The Jackson Laboratory. C57BL/6Ncr (B6/Ncr) mice used as a source of fertilized one-cell embryos for microinjection were purchased from Charles River Laboratories. P14 mice (C57BL/6Ai-[Tg]TCR P14 LCMV N10) were purchased from Taconic Biosciences^45^. hCD2^Cre^ mice (C57BL/6-Tg(CD2-cre)1Lov/J) and SAP KO mice (B6.129S6-*Sh2d1a*^*tm1Pls*^/J) were a kind gift from Dr. Paul. E. Love (NICHD, NIH) and Dr. Pamela L. Schwartzberg (NIAID, NIH), respectively^35,68^. Mice were housed in specific pathogen-free facilities. Animal rooms operate on a 12-h light cycle, with lights turning on at 6 am and off at 6 pm. Temperature stays around 21–23 °C. Humidity is maintained between 30% and 70%. Both female and male mice at 8–20 weeks of age were used except for adoptive T cell transfer experiments where only females were used as a source of donor T cells. Animal procedures were approved by the NCI Animal Care and Use Committee.

### Generation of Lcp2^L8:21L-mE8^ mice

We utilized the CRISPR/Cas9 system to generate *Lcp2*^*L8:21L-mE8*^ mice. Briefly, four single guide (sg) RNAs were designed using sgRNA Scorer 2.0 (PMID: 28146356) and subsequently tested for in vitro cutting activity measured by high throughput sequencing of which sgRNA-1: AGAGCCGTTTAATTCCATCC and sgRNA-2: AACCACTGAACAGAACGTGA were chosen for subsequent mouse generation. To generate the donor/targeting vector, a double stranded plasmid DNA was first cloned using a combination of synthesized DNA fragments (Twist Bioscience) and isothermal assembly (PMID: 19363495) into the pGMC00018 backbone (Addgene #195320). This plasmid consisted of WT SLP-76 cDNA spanning exons 8 through 21 followed by a poly A sequence, which was flanked by LoxP sequences. The second LoxP sequence was followed by the mutated exon 8 encoding the SLP-76^HA^. Upon Sanger sequence validation of this construct, a long single stranded DNA (ssDNA) was then generated using the Guide-it Long ssDNA Production system (Takara Bio). Finally, Cas9 protein, chemically modified versions of the above listed sgRNAs (Synthego) targeting the upstream and downstream introns of exon 8, and the long ssDNA fragment were then microinjected into fertilized one-cell embryos of B6/Ncr mice. After microinjection, the embryos were transferred into the oviduct of pseudo-pregnant B6D2F1 females. Offspring from the recipient females were genotyped to identify a founder mouse carrying the successfully recombineered *Lcp2*^*L8:21L-mE8*^ allele by PCR using two primers, GT-1F: CATGCACACGGATCATGTTGC and GT-2R: ACTGGTGTGAAGTAGCTGCC. The founder mouse was backcrossed onto B6/J mice at least 8 generations prior to further breeding as described below.

### Mouse breeding

To evaluate the effect of the SLP-76^HA^ mutation on thymocyte development and on peripheral T cell activation and differentiation, *Lcp2*^*L8:21L-mE8*^ mice were bred onto *CD4*^*Cre*^ mice, *pLck*^*Cre*^ mice, and *hCD2*^*Cre*^ mice, respectively. *Lcp2*^*L8:21L-mE8*^ mice carrying a CD4^Cre^, pLck^Cre^, or hCD2^Cre^ transgene were further mated with *Rosa26*^*LSL-eYFP*^ mice to induce YFP expression in cells with Cre activity, thus marking SLP-76^HA^-expresssing cells. *Rosa26*^*LSL-eYFP*^ mice carrying a CD4^Cre^, pLck^Cre^, or hCD2^Cre^ transgene were used as a control. To assess the effect of the SLP-76^HA^ mutation on thymocyte development and on antigen-specific T cell responses, *Lcp2*^*L8:21L-mE8*^ × *Rosa26*^*LSL-eYFP*^ × *CD4*^*Cre*^ mice and *Lcp2*^*L8:21L-mE8*^ × *Rosa26*^*LSL-eYFP*^ × *hCD2*^*Cre*^ mice were further crossed onto OT-I, P14, or OT-II TCR transgenic mice. *Rosa26*^*LSL-eYFP*^ × *CD4*^*Cre*^ mice and *Rosa26*^*LSL-eYFP*^ × *hCD2*^*Cre*^ mice carrying a corresponding TCR transgene were used as a control. In all mouse lines generated by the genetic cross, the *Lcp2*^*L8:21L-mE8*^ and *Rosa26*^*LSL-eYFP*^ alleles were maintained homozygous, but the Cre and TCR transgenes were maintained hemizygous. B6 CD45.1 mice were bred onto B6/J mice and SAP KO mice to generate (CD45.1 × CD45.2) F1 mice and SAP KO CD45.1 mice, respectively. These mice were used as recipients for adoptive T cell transfer experiments. All the mice used in this study were euthanized by CO_2_ inhalation.

### Antibodies and other reagents for flow cytometry and ImageStream analyses

Biotinylated anti-γδTCR (GL3, 1:1000 dilution), BUV395 anti-CD4 (RM4-5, 1:200), BUV395 anti-CD19 (1D3, 1:100), BUV737 anti-CD45.2 (104, 1:200), BUV737 anti-TCRβ (H57-597, 1:200), BUV805 anti-CD38 (90/CD38, 1:200), BUV805 anti-CD44 (IM7, 1:200), BV421 anti-Vα2 TCR (B20.1, 1:100), BV421 anti-NK1.1 (PK136, 1:200), BV510 anti-CD19 (1D3, 1:100), BV510 anti-γδTCR (GL3, 1:100), BV650 anti-γδTCR (GL3, 1:100), BV711 anti-CD24 (M1/69, 1:400), BV711 anti-CD95 (Jo2, 1:100), BV711 anti-CD103 (M290, 1:200), BV786 anti-Vα2 TCR (B20.1, 1:200), RB705 anti-GL7 (GL7, 1:2000), RB705 anti-TCF1 (S33-966, 1:200), PE anti-SLP-76 (H3, 1:10), PE-CF594 anti-Blimp-1 (5E7, 1:200), PE-Cy7 anti-CD45.1 (A20, 1:200), Alexa Flour 647 anti-Bcl-6 (K112-91, 1:60), Alexa Flour 647 anti-ERK1/2 pT202/pY204 (20A, 1:10), and R718 anti-CD3 (17A2, 1:500) were purchased from BD Biosciences. Biotinylated antibodies to CD3 (17A2, 1:1000), CD4 (RM4-5, 1:1000), CD8a (53-6.7, 1:1000), CD11b (M1/70, 1:1000), CD11c (N418, 1:1000), CD25 (PC61, 1:1000), CD49b (DX5, 1:1000), CD69 (H1.2F3, 1:1000), CD90.2 (30-H12, 1:1000), NK1.1 (PK136, 1:1000), ICOS (C398.4A, 1:1000), PD-1 (29F.1A12, 1:1000), I-A/I-E (M5/114.15.2, 1:1000), and TER-119 (TER-119, 1:1000), BV421 anti-CCR7 (4B12, 1:60), BV421 anti-CXCR5 (L138D7, 1:100), BV421 anti-PD-1 (29F.1A12, 1:100), BV421 anti-KLRG1 (2F1/KLRG1, 1:100), BV510 anti-I-A/I-E (M5/114.15.2, 1:200), BV510 anti-NK1.1 (PK136, 1:100), BV510 anti-H-2K^b^ (AF6-88.5, 1:200), BV510 anti-IgD (11-26c.2a, 1:200), BV605 anti-CD62L (MEL-14, 1:200), BV650 anti-mouse IgG1 (RMG1-1, 1:400), BV785 anti-CD138 (281-2, 1:200), BV785 anti-I-A/I-E (M5/114.15.2, 1:1000), BV785 anti-Ly-6C (HK1.4, 1:1000), BV785 anti-PD-1 (29F.1A12, 1:200), Alexa Flour 488 anti-GFP (FM264G; This antibody cross-reacts with YFP, 1:200), PerCP-Cy5.5 anti-CD5 (53-7.3, 1:200), PerCP-Cy5.5 anti-CD25 (PC61, 1:200), PE anti-PD-1 (29F.1A12, 1:200), PE-Dazzle 594 anti-CD122 (TM-β1, 1:100), PE-Dazzle 594 anti-CCR7 (4B12, 1:20), PE-Dazzle 594 anti-CXCR5 (L138D7, 1:100), PE-Cy5 anti-CD127 (A7R34, 1:100), PE-Cy5 anti-mouse IgM (RMM-1, 1:200), PE-Cy7 anti-CD5 (53-7.3, 1:500), PE-Cy7 anti-CD25 (PC61, 1:500), APC anti-CD69 (H1.2F3, 1:200), APC anti-Helios (22F6, 1:200), APC-Cy7 anti-CD19 (6D5, 1:200), purified anti-CD16/CD32 (93, 10 µg/mL), and DAPI (100 ng/mL) were purchased from BioLegend. Biotinylated antibodies to CD16/CD32 (93, 1:1000) and CD19 (1D3, 1:1000), PE-eFluor 610 anti-Foxp3 (FJK-16s, 1:100), and APC-eFluor 780 anti-CD8a (53-6.7, 1:200) were purchased from eBiosciences. PE anti-Cleaved Caspase-3 (D3E9, 1:100) was purchased from Cell Signaling. PE-conjugated NP (4-hydroxy-3-nitrophenylacetyl, 10 µg/mL) was purchased from LGC Biosearch Technologies and Creative Diagnostics. PE-conjugated PBS-57/CD1d tetramer (1:200) and PE-conjugated LCMV_gp33-41_/H-2D^b^ tetramer (1:400) were provided by NIH Tetramer Core Facility.

### Isolation of T cells and T cell-depleted spleen cells

A single cell suspension prepared from whole LNs (axillary, brachial, superficial/deep cervical, inguinal, and mesenteric) and spleens of T cell donor mice was treated with ACK lysis buffer (KD Medicals) to deplete red blood cells (RBC). The remaining cells were suspended in 1X PBS (KD Medicals) containing 2% fetal bovine serum (FBS; Gibco) and incubated with biotinylated antibodies to CD4 (for CD8^+^ T cell isolation), CD8a (for CD4^+^ T cell isolation), CD19, I-A/I-E, CD16/CD32, NK1.1, CD25, γδTCR, CD11b, CD11c, CD49b, CD69, ICOS, PD-1, and TER-119. After washing extensively, the antibody-bound cells were incubated with Streptavidin Microbeads (Miltenyi) and loaded onto LS Columns (Miltenyi), following the manufacturer’s instruction. The magnetically unbound cells were used for an in vitro T cell activation assay and for adoptive transfer to assess the responsiveness of donor T cells in vivo. For isolation of T cell-depleted spleen (TdS) cells, a single cell suspension was prepared from spleens of (CD45.1 × CD45.2) F1 mice. Most of the procedures were the same as the T cell isolation except that the remaining cells after RBC lysis were incubated with biotinylated antibodies to CD3, γδTCR, and CD90.2. The magnetically unbound cells were used as antigen-presenting cells for in vitro T cell activation assay.

### In vitro OT-I CD8^+^ T cell activation for induction of CD69 and pERK

TdS cells from (CD45.1 × CD45.2) F1 mice were loaded with varying concentrations of WT OVA peptide N4 (SIINFEKL; Anaspec), Q4H7 (SIIQFEHL; Anaspec), or G4 (SIIGFEKL; Anaspec) in 200 µL of RPMI 1640 (Gibco) containing 5% FBS, 1 mM Sodium Pyruvate (Gibco), 1X MEM Non-Essential Amino Acids (Gibco), 2 mM L-Glutamine (Gibco), and 100 U/mL Penicillin-Streptomycin (Gibco) (thereafter cRPMI) on a 96-well round bottom cell culture plate (Coster) at 37 °C for 3 h in a humidified CO_2_ incubator. After peptide loading, TdS cells were washed 3 times with 1X PBS. CD8^+^ T cells isolated from WT CD45.1 OT-I and SLP-76^2Cre-mE8^ CD45.2 OT-I mice were mixed at a 1:1 ratio (5 × 10^4^ cells/well of each genotype) and stimulated with peptide-loaded TdS cells (1 × 10^5^ cells/well) on a 96-well round bottom cell culture plate (200 µL of cRPMI/well). After 20 h of incubation, primed OT-I cells were subject to CD69 staining. For pERK analysis by ImageStream, cell cultures were set up as described above except that TdS cells were pre-loaded with 10^−11 ^M N4 or 10^−9^ Q4H7 peptide. After spinning culture plates for 3 min to form a pellet, cells were incubated in a humidified CO_2_ incubator for the indicated time. Primed OT-I cells were fixed with Cytofix/Cytoperm Solution (BD Biosciences) and permeabilized with ice-cold methanol. The fixed/permeabilized cells were subject to pERK staining for ImageStream analysis.

### Adoptive T cell transfer and immunization

CD8^+^ T cells isolated from WT CD45.1 OT-I and SLP-76^2Cre-mE8^ CD45.2 OT-I mice were mixed at a 1:1 ratio and labeled with 5 µM CTV (Invitrogen) following the manufacturer’s instruction. After CTV labeling and extensive washing with 1X PBS, cells were suspended in 1X PBS at 1 × 10^6^ cells/mL. (CD45.1 × CD45.2) F1 mice received 100 µL of cell suspension (containing 5 × 10^4^ OT-I cells of each genotype) intravenously via retro-orbital sinus. One day later, recipient mice were immunized with 10 nmol of Q4H7 emulsified in complete Freund’s adjuvant (CFA; Sigma) subcutaneously via left and right lateral. Donor OT-I cells in skin dLNs (axillary, brachial, and inguinal) were analyzed at 3 days post-immunization by flow cytometry.

To assess the responsiveness of CD4^+^ T cells in vivo, 1:1-mixed OT-II cells isolated from WT OT-II CD45.1 and SLP-76^2Cre-mE8^ OT-II CD45.2 mice were suspended in 1X PBS at 1 × 10^6^ cells/mL and injected into (CD45.1 × CD45.2) F1 mice with a volume of 100 µL containing 5 × 10^4^ OT-II cells of each genotype. One day later, recipient mice were immunized with 50 µg of EndoFit OVA (Invivogen) in CFA subcutaneously via left and right lateral. Donor OT-II cells in skin dLNs were analyzed at 6, 8, and 10 days post-immunization by flow cytometry. In some experiments, WT, SLP-76^2Cre-mE8^, or SAP KO OT-II cells (all CD45.2; 1 × 10^5^ cells/mouse) were transferred into SAP KO CD45.1 mice. One day later, recipient mice were immunized with 50 µg of NP-OVA (LGC Biosearch Technologies and Creative Diagnostics). Ten days after immunization, cells from skin dLNs were analyzed for Tfh cells (CD45.2 OT-II donor cells) and GC B cells (SAP KO CD45.1 cells).

### LCMV infection

CD8^+^ T cells isolated from WT P14 CD45.1 and SLP-76^2Cre-mE8^ P14 CD45.2 mice were mixed at a 1:1 ratio and suspended in 1X PBS at 1 × 10^5^ cells/mL. (CD45.1 × CD45.2) F1 mice were injected with 100 µL of cell suspension (containing 5 × 10^3^ P14 cells of each genotype) in PBS intravenously via retro-orbital sinus. One day later, recipient mice were infected intraperitoneally with 5 × 10^4^ plaque-forming units of LCMV_Arm_. Donor P14 cells in the spleens of infected recipients were analyzed at 7 and 28 days post-infection by flow cytometry. In some experiments, WT and SLP-76^2Cre-mE8^ mice bearing endogenous TCR repertoire were infected with the same dose of LCMV_Arm_ to assess the virus-specific CD8^+^ T cells at 30 days post-infection.

### Cell preparation and staining for flow cytometry and ImageStream analyses

Thymi or pooled whole LNs (axillary, brachial, superficial/deep cervical, inguinal, and mesenteric) of WT, SLP-76^4Cre-mE8^, and SLP-76^pLCre-mE8^ mice at 8–12 weeks of age were mashed on a 40 µm cell strainer, and thymocytes or LN cells were suspended in 1X PBS containing 2% FBS. Cells were stained with Blue Fluorescent Reactive Dye (Invitrogen) for dead cell exclusion, followed by staining for surface molecules in the presence of purified anti-CD16/CD32 to prevent FcR-mediated non-specific binding. In some experiments, thymocytes were stained with PE-conjugated PBS-57/CD1d tetramer in the presence of 2 nM Dasatinib (Selleckchem) to prevent the downregulation of the tetramer-bound TCR. The tetramer staining was performed prior to staining for surface markers. The stained cells were fixed/permeabilized first with Cytofix/Cytoperm Solution and then with Foxp3/Transcription Factor Fixation/Permeabilization Solution (eBiosciences), followed by staining for cytoplasmic and nuclear molecules. This serial fixation/permeabilization method was very effective in co-staining for transcription factors and YFP (using Alexa Fluor 488 anti-GFP mAb) that was quenched by a single fixation with Foxp3/Transcription Factor Fixation/Permeabilization Solution.

For SLP-76 staining, thymocytes or LN cells were stained with Blue Fluorescent Reactive Dye, fixed with Cytofix/Cytoperm Solution, and then permeabilized with ice-cold methanol. These cells were stained with PE anti-SLP-76 and Alexa Fluor 488 anti-GFP in combination with mAbs against lineage-specific markers.

To analyze in vitro activated OT-I cells for CD69 expression, cultured cells were stained with Blue Fluorescent Reactive Dye and then with mAbs against surface molecules in the presence of purified anti-CD16/CD32. After surface staining, cells were treated with Cytofix/Cytoperm Solution and stained with Alexa Fluor 488 anti-GFP. For pERK analysis by ImageStream, after primed OT-I cells were fixed and permeabilized (described above in “In vitro *OT-I CD8*^*+*^ *T cell activation for induction of CD69 and pERK*”), they were stained with Alexa Flour 647 anti-ERK1/2 pY202/pT204 and Alexa Fluor 488 anti-GFP in combination with mAbs against lineage-specific markers.

To analyze donor T cells or recipient-derived B cells for in vivo activation and differentiation after immunization with cognate antigens or infection with LCMV_Arm_, cells from skin dLNs or spleens were stained with Blue Fluorescent Reactive Dye, followed by staining with mAbs against surface molecules in the presence of purified anti-CD16/CD32. After surface staining, those cells were subject to the serial fixed/permeabilized method as described above, followed by staining with Alexa Flour 488 anti-GFP in combination with mAbs against cytoplasmic molecules and transcription factors. In the experiments where non-TCR transgenic mice were infected with LCMV_Arm_, virus-reactive CD8^+^ T cells were detected by staining with PE-conjugated LCMV_gp33-41_/H-2D^b^ tetramer in the presence of 2 nM Dasatinib prior to surface marker staining. In the experiments where SAP KO CD45.1 mice receiving CD45.2 OT-II donor cells were immunized with NP-OVA, NP-specific GC B cells were detected by staining with PE-conjugated NP in the presence of 2 nM Dasatinib prior to surface marker staining.

Stained cell samples were run on the FACSymphony A5 (BD Biosciences) or ImageStream Mk II (Cytec Biosciences). The acquired flow cytometry and ImageStream data were analyzed with FlowJo 10 software (BD Biosciences) and IDEAS 6.03 (Cytec Biosciences), respectively. Gating strategies to analyze the populations of interest were shown in main figures and Supplementary Information.

### Statistical analyses

All statistical analyses were done with Prism 10 software (GraphPad Software). Unless noted otherwise in the figure legends, all experiments were performed at least 3 times. Bars and errors in graphs indicate mean ± SD. Comparisons were done using Mann-Whitney test, Wilcoxon matched-pairs signed rank test, ordinary one-way ANOVA, Kruskal-Wallis test, or two-way ANOVA. Exact *p*-values were shown in all graphs, and non-significant results were marked as “ns” or not marked. For statistical comparisons, sample size was greater than five except for the experiments using Lcp2^pLCre-mE8^ mice and determined empirically based on pilot analyses. Animals were excluded from analyses only because of poor health status unrelated to the experiment.

### Reporting summary

Further information on research design is available in the Nature Portfolio Reporting Summary linked to this article.

## Supplementary information


Supplementary Information
Transparent Peer Review file
Reporting Summary


## Source data


Source Data


## Data Availability

All data required for the authors to draw the conclusions are present in the current study and available from the corresponding authors. Reagents used in the current study will be available upon request to the corresponding authors after completing a materials transfer agreement with NCI, NIH. Source data are provided with this paper.
