# Supplementary material for: Weak SLP-76/PLC-γ1 interaction fine-tunes T cell receptor signal strength to optimize T cell responsiveness
Source: Nat Commun. 2026 Jul 29;17:9189. doi: 10.1038/s41467-026-75599-0 (PMC13522589; doi:10.1038/s41467-026-75599-0)
Supplement: Supplementary file 1 [file 41467_2026_75599_MOESM1_ESM.pdf]

Supplementary Information

Weak SLP-76/PLC-γ1 interaction fine-tunes T cell receptor signal strength to optimize T cell responsiveness

Hidehiro Yamane*, Junya Wada, Elizabeth N. Stassenko, Natalie D. Convertino, Mariah E. Lee, Melanie S. Vacchio, Wenmei Li, Ferenc Livak, Udumbara M. Rathnayake, Lakshmi Balagopalan, Raj Chari, Herbert Hagenau, Parirokh Awasthi, Dorian B. McGavern, Remy Bosselut, and Lawrence E. Samelson*

*Corresponding Authors:
Hidehiro Yamane (hidehiro.yamane@nih.gov)
Lawrence E. Samelson (samelsonl@mail.nih.gov)

This file includes:
Supplementary Figures 1–8
Supplementary Table 1

a

SH3 domains of PLC-γ1 in vertebrates

Class/Order	Genus/Species	Sequences (791-851aa of human PLC-γ1)
Mammals (Placentals)	Human	T F K C A V K A L F D Y K A Q R E D E L T F I K S A I I Q N V E K Q E G G W W R G D Y G G K K Q L W F P S N Y V E E M V N
	Monkey	T F K C A V K A L F D Y K A Q R D D E L T F T K S A I I Q N V E K Q E G G W W R G D Y G G K K Q L W F P S N Y V E E M V S
	Mouse	T F K C A V K A L F D Y K A Q R E D E L T F T K S A I I Q N V E K Q D G G W W R G D Y G G K K Q L W F P S N Y V E E M I N
	Rabbit	T F K C A V K A L F D Y K A Q R E D E L T F T K S A I I Q N V E K Q E G G W W R G D Y G G K K Q L W F P S N Y V E E M V S
	Cow	T F K C A V K A L F D Y K A Q R E D E L T F T K S A I I Q N V E K Q E G G W W R G D Y G G K K Q L W F P S N Y V E E M V S
	Bat	T F K C A V K A L F D Y K A Q R D D E L T F T K S A I I Q N V E K Q D G G W W R G D Y G G K K Q L W F P S N Y V E E M V S
	Shrew	T F K C A V K A L F D Y K A Q R E D E L T F T K S A I I Q N V E K Q E G G W W R G D Y G G K K Q L W F P S N Y V E E M V S
	Elephant	T F K C A V K A L F D Y K A Q R E D E L T F T K S A I I Q N V E K Q E G G W W R G D Y G G K K Q L W F P S N Y V E E M V S
Mammals (Marsupials)	Koala	T F K C A V K A L F D Y K A Q R E D E L T F T K S A I I Q N V E K Q E G G W W R G D Y G G K K Q L W F P S N Y V E E M A N
	Tasmanian devil	T F K C A V K A L F D Y K A Q R E D E L T F T K S A I I Q N V E K Q E G G W W R G D Y G G K K Q L W F P S N Y V E E M A N
Birds	Turkey	V A Q C T V K A L Y D Y K A Q R E D E L S F C K Q A I I H N V D K Q D G G W W R G D Y G G K K Q L W F P A N Y V E E I V S
	Ostrich	T F K C A V K A L F D Y K A Q R E D E L T F T K N A I I Q N V E K Q E G G W W R G D Y G G K K Q L W F P S N Y V E E I T S
Reptiles	Crocodile	T F K C A V K A L F D Y K A Q R D D E L T F T K N A I I Q N V E K Q E G G W W R G D Y G G K K Q L W F P S N Y V E E I V S
	Lizard	T F K C A V K A L F D Y R A Q R E D E L S F T K N A I I Q N V E K Q D G G W W R G D Y G G K K Q L W F P S N Y V E E I A N
	Turtle	T F K C A V K A L F D Y K A Q R E D E L T F M K N A I I Q N V E K Q E G G W W R G D Y G G K K Q L W F P S N Y V E E L A S
Amphibians	Frog	T F K C S V R A L F D Y K A Q R E D E L T F T K N T I I Q N V E K Q E G G W W R G D C G G K K Q M W F P A N Y V E E I F S
Fishes	Coelacanth (Lobe-finned)	V F K C A V K A L Y D Y K A Q R E D E L S F T K K A I I Q N V E K Q E G G W W K G D Y G G K K Q L W F P A N Y I E E L P S
	Zebrafish (Ray-finned)	T F K C T V K A M Y E Y K A Q R D D E L S F I K N A I I S N V D K Q E G G W W K G D C G G K K Q L W F P A N Y V E E I S -
	Shark (Cartilaginous)	M F K C T V K A M Y D Y R Q Q R D D E L S F C K H A I I Q N V D K Q D G G W W K G D Y G G K R Q L W F P A N Y V E E I T S

b

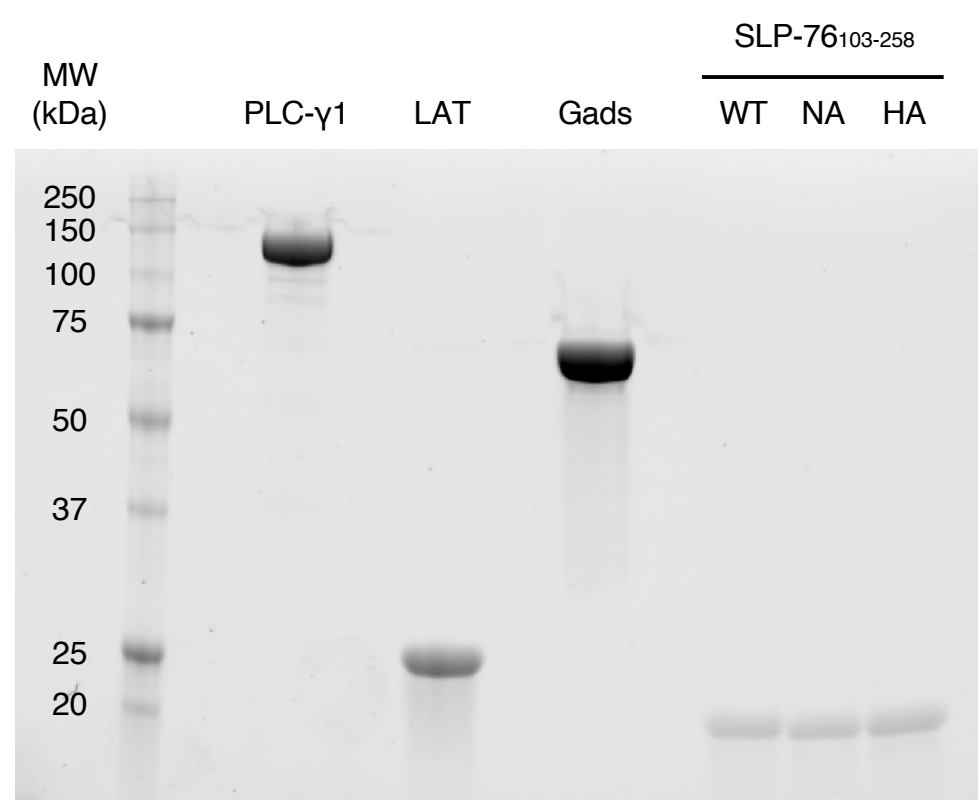

c

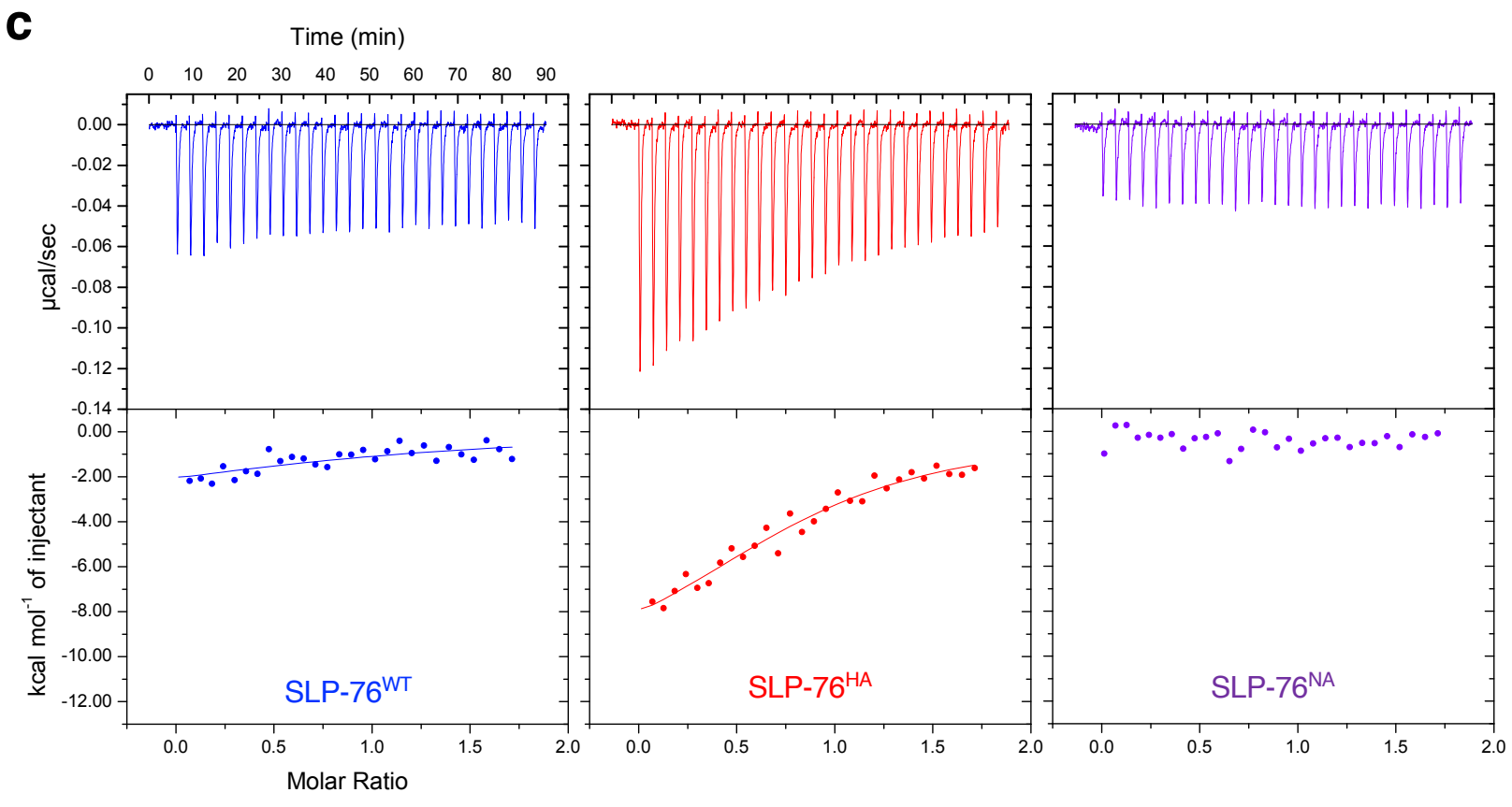

d

SH3 domains of PLC-γ1, Itk, and Lck

	Sequence (808, 828, 844, 845 marked above PLC-γ1 row)	Identity (%)
PLC-γ1	T F K C A V K A L F D Y K A Q R E D E L T F I K S A I I Q N V E K Q E G G W W R G D - Y G G K K Q L W F P S N Y V E E M V N	100
Itk	P E E T V V I A L Y D Y Q T N D P Q E L A L R R N E E Y C L L D S S E I H W W R V Q - D R N G H E G Y V P S S Y L V E K S P	24.6
Lck	L Q D N L V I A L H S Y E P S H D G D L G F E K G E Q L R I L E Q - S G E W W K A Q S L T T G Q E G F I P F N F V A K A N S	23.3

(791-851aa of human PLC-γ1, 171-231aa of human Itk, 61-121aa of human Lck)

e

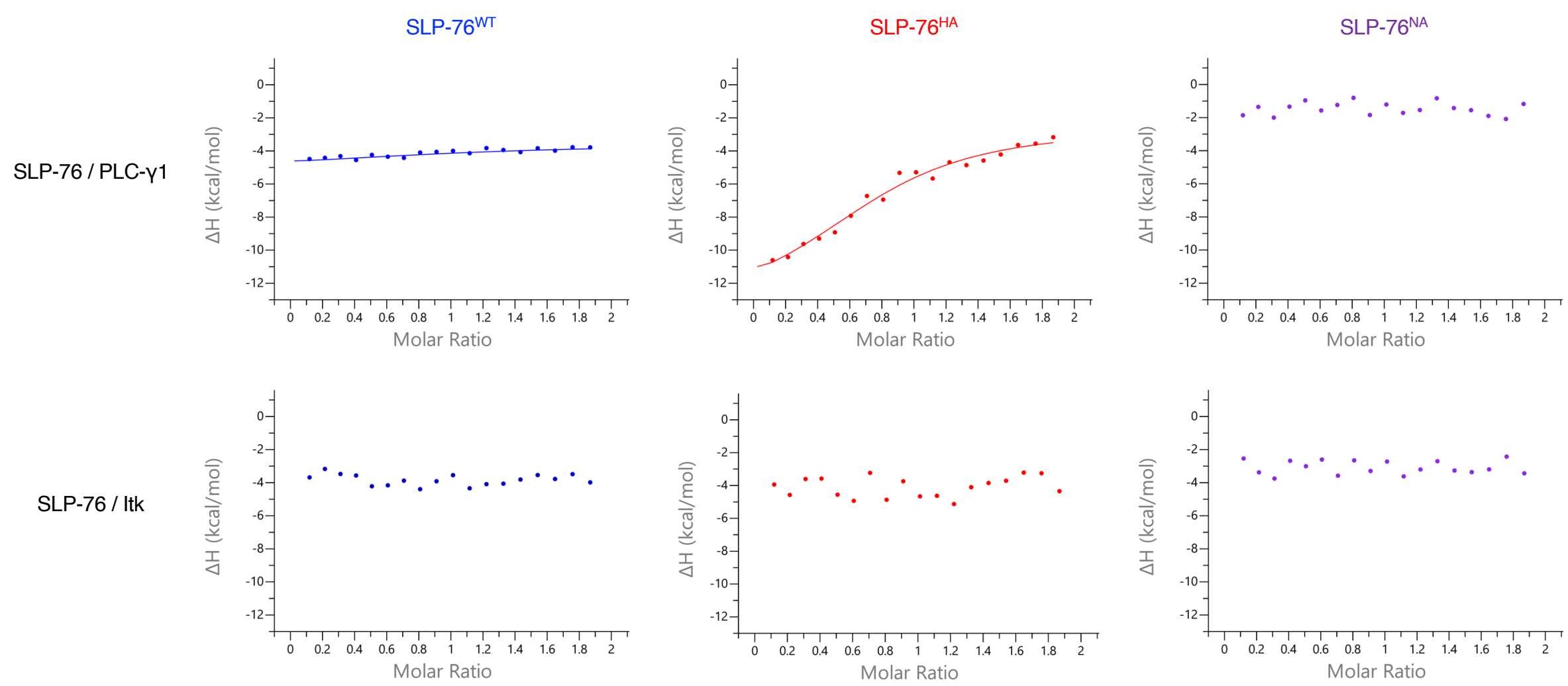

f

Proline-rich domains of SLP-76 and LAT

	Sequence	Identity (%)
SLP-76	- - G K - - - T P Q Q P P V P P Q R - - - - - - - - P M A A L P P P P A G R N H S	100
LAT	P H T V A P W P P A Y P P V T S Y P P L S Q P D L L P I P R S P Q P L G G - - - -	33.3

(180-207aa of human SLP-76, 54-90aa of human LAT)

Supplementary Figure 1: *In vitro* reconstitution studies of tetramer formation.
a, Sequence alignment of the SH3 domain of PLC-γ1 among 19 jawed vertebrates. **b**, Purified recombinant proteins used in the current study were resolved by SDS-PAGE and imaged with the Chemi-doc system. **c**, Spectrum of isothermal titration calorimetry (ITC) for the interaction of SLP-76 peptides with PLC-γ1 measured by VP-ITC. **d**, Sequence alignment of the SH3 domains of PLC-γ1, Itk, and Lck. PLC-γ1 residues D808, W828, N844, and Y845, in green, are critical residues for binding SLP-76 (Ref. 8). In both Itk and Lck, multiple residues in red corresponding to those critical residues in PLC-γ1 are mutated. **e**, Spectrum of ITC for the interaction of SLP-76 with PLC-γ1 or Itk, measured by PEAQ-ITC. **f**, Sequence alignment of the proline-rich regions of SLP-76 and LAT. In the SLP-76 sequence, prolines and arginine in red are critical residues for binding to PLC-γ1. In the LAT sequence, amino acids in green are critical residues for binding to Lck (Ref. 14).

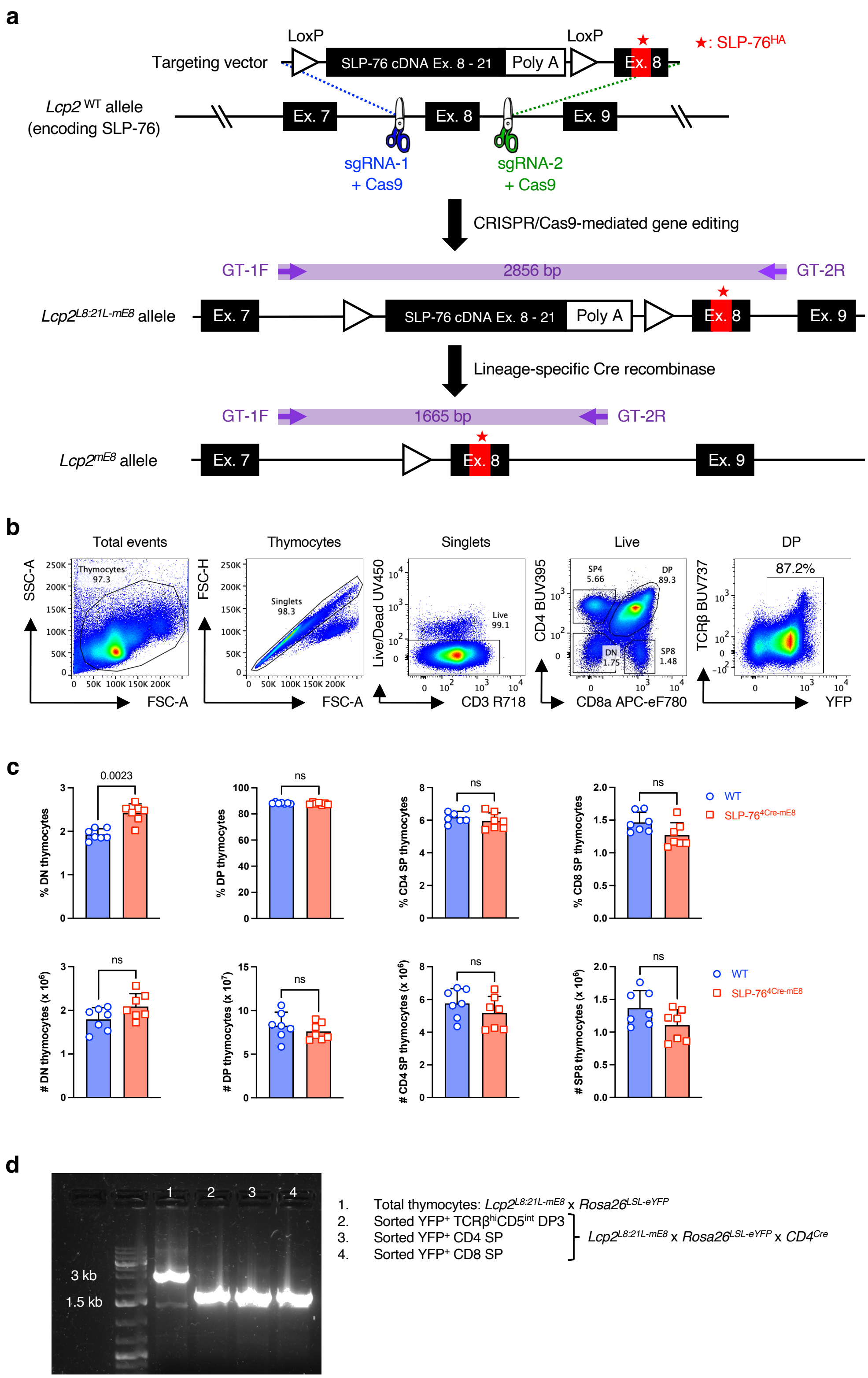

a
Targeting vector
LoxP
SLP-76 cDNA Ex. 8 - 21
Poly A
LoxP
Ex. 8
★: SLP-76HA
Lcp2 WT allele
(encoding SLP-76)
Ex. 7
Ex. 8
Ex. 9
sgRNA-1
+ Cas9
sgRNA-2
+ Cas9
CRISPR/Cas9-mediated gene editing
GT-1F
2856 bp
GT-2R
Lcp2L8:21L-mE8 allele
Ex. 7
SLP-76 cDNA Ex. 8 - 21
Poly A
Ex. 8
Ex. 9
Lineage-specific Cre recombinase
GT-1F
1665 bp
GT-2R
Lcp2mE8 allele
Ex. 7
Ex. 8
Ex. 9
b
Total events
Thymocytes 97.3
SSC-A
FSC-A
Thymocytes
Singlets 98.3
FSC-H
FSC-A
Singlets
Live 99.1
Live/Dead UV450
CD3 R718
Live
SP4 5.66
DP 89.3
DN 1.75
SP8 1.48
CD4 BUV395
CD8a APC-eF780
DP
87.2%
TCRβ BUV737
YFP
c
% DN thymocytes
0.0023
% DP thymocytes
ns
% CD4 SP thymocytes
ns
% CD8 SP thymocytes
ns
WT
SLP-764Cre-mE8
DN thymocytes (x 10^6)
ns
DP thymocytes (x 10^7)
ns
CD4 SP thymocytes (x 10^6)
ns
SP8 thymocytes (x 10^6)
ns
WT
SLP-764Cre-mE8
d
1
2
3
4
3 kb
1.5 kb
1. Total thymocytes: Lcp2L8:21L-mE8 x Rosa26LSL-eYFP
2. Sorted YFP+ TCRβhiCD5int DP3
3. Sorted YFP+ CD4 SP
4. Sorted YFP+ CD8 SP
Lcp2L8:21L-mE8 x Rosa26LSL-eYFP x CD4Cre

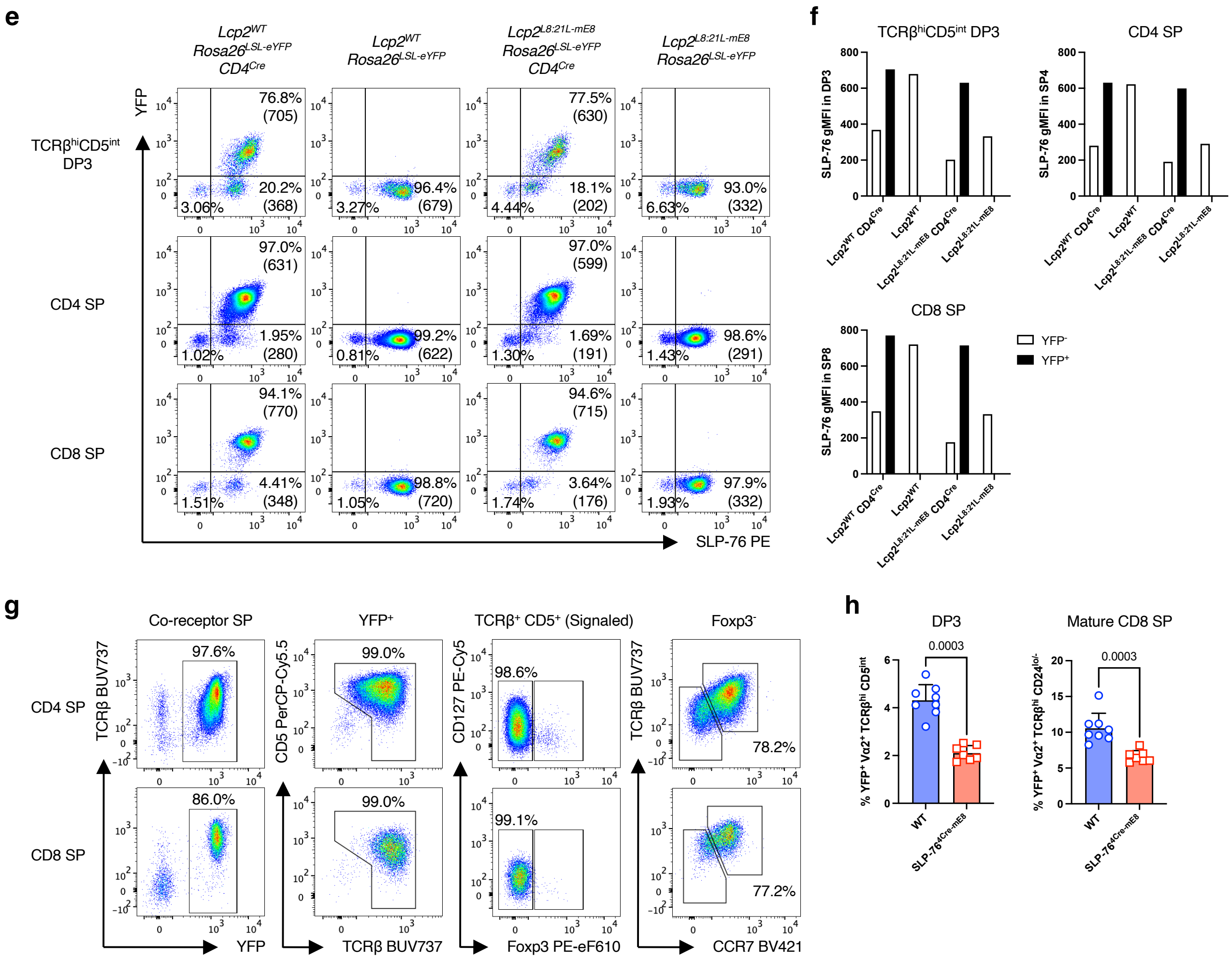

Supplementary Figure 2: Generation of Lcp2$^{\text{L8:21L-mE8}}$ mice, verification of CD4^{Cre}-mediated SLP-76^{HA} mutation, and FACS analysis of SLP-76$^{\text{4Cre-mE8}}$ thymocytes.

a, Diagram of gene targeting strategy to generate an Lcp2$^{\text{L8:21L-mE8}}$ mouse and to achieve a lineage-specific Cre-mediated SLP-76^{HA} mutation. Genomic PCR using the GT-1F/GT-2R primer set distinguishes the unmutated Lcp2$^{\text{L8:21L-mE8}}$ (2856 bp) and mutated Lcp2$^{\text{mE8}}$ (1665 bp) alleles. **b**, Gating strategy for analysis of YFP^{+} CD4/CD8 DP thymocytes. **c**, Statistical analysis for the frequency (upper row) and number (lower row) of DN, DP, CD4 SP, and CD8 SP thymocytes from WT and SLP-76$^{\text{4Cre-mE8}}$ mice (n=7 for each group). **d**, Verification of a conditional SLP-76^{HA} mutation in sorted TCRβ^{hi} CD5^{int} DP3, CD4 SP, and CD8 SP thymocytes (all YFP^{+}) of the Lcp2$^{\text{L8:21L-mE8}}$ x Rosa26$^{\text{LSL-eYFP}}$ x CD4^{Cre} mouse by genomic PCR. **e**, SLP-76 and YFP expression in TCRβ^{hi} CD5^{int} DP3, CD4 SP, and CD8 SP thymocytes of the Lcp2^{WT} and Lcp2$^{\text{L8:21L-mE8}}$ mice with or without CD4^{Cre} (all carrying the Rosa26$^{\text{LSL-eYFP}}$ allele). Numbers in parentheses indicate a geometric mean fluorescence intensity (gMFI) of SLP-76 staining in each quadrant. **f**, Bar graphs for gMFI of SLP-76 generated from the flow cytometric analysis (**e**). Shown is a representative of 3 independent experiments. **g**, Gating strategy for analysis of YFP^{+} CD4 SP (upper row) and CD8 SP (lower row) thymocytes. **h**, Statistical analysis for the frequency of YFP^{+} TCRVα2^{+} TCRβ^{hi} CD5^{int} DP3 (left) and YFP^{+} TCRVα2^{+} TCRβ^{hi} CD24$^{\text{lo/-}}$ mature CD8 SP (right) thymocytes from WT OT-I (n=8) and SLP-76$^{\text{4Cre-mE8}}$ OT-I (n=7) mice. Each symbol represents one mouse. Data were shown as mean ± SD, and statistics was determined by Mann-Whitney test (**c**,**h**). ns; not significant.

a

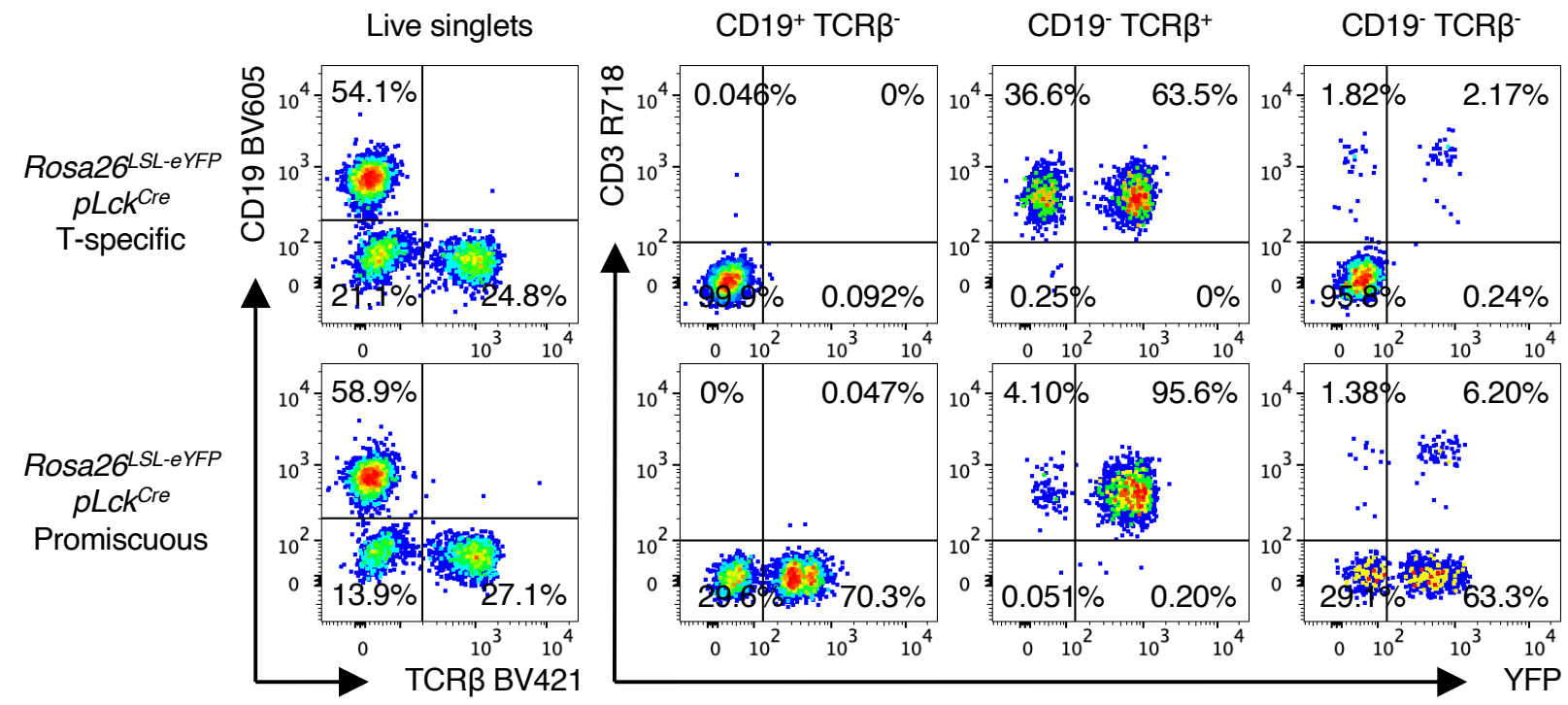

b

	SLP-76WT	SLP-76pLCre-mE8
# T cell-specific	5	4
# Promiscuous	11	8
% T cell-specific	31.3	33.3

c

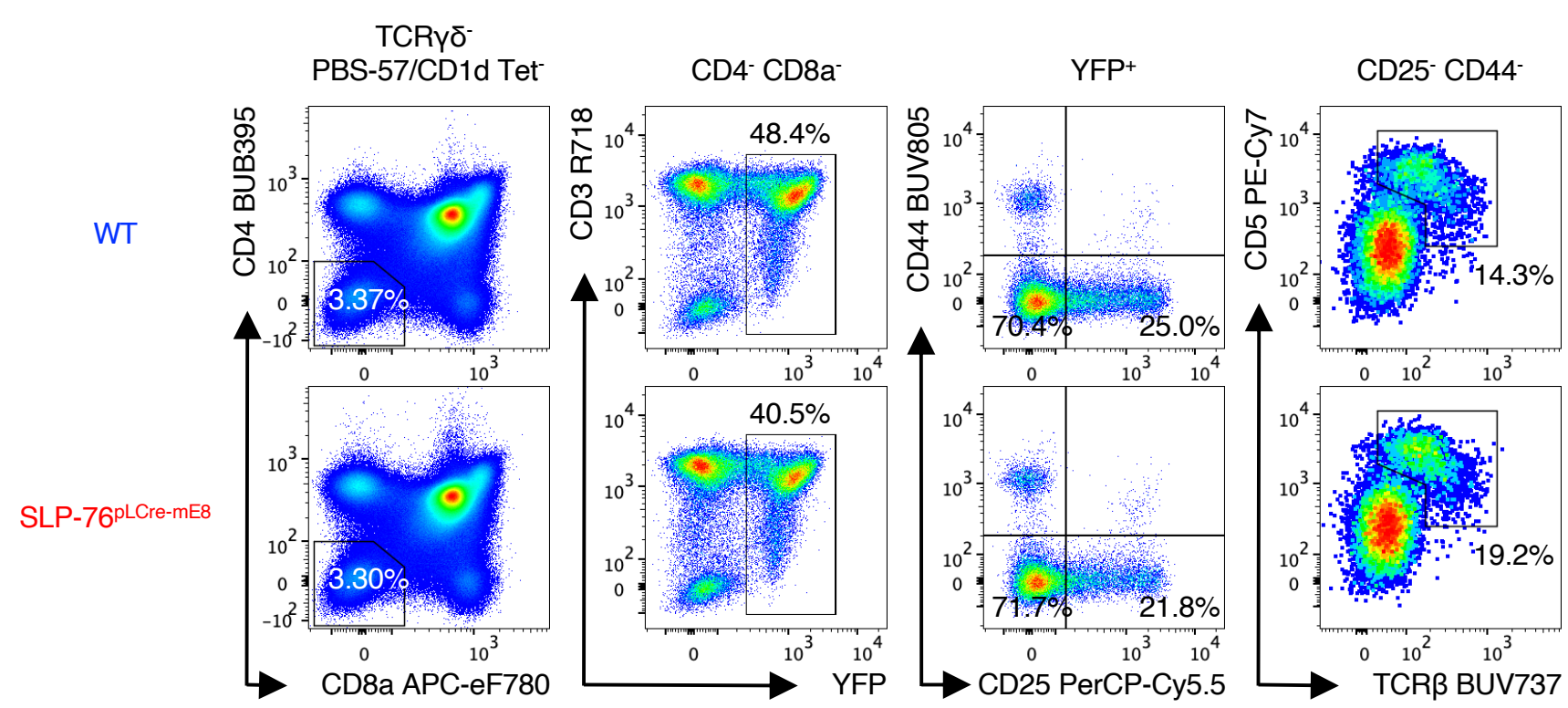

d e

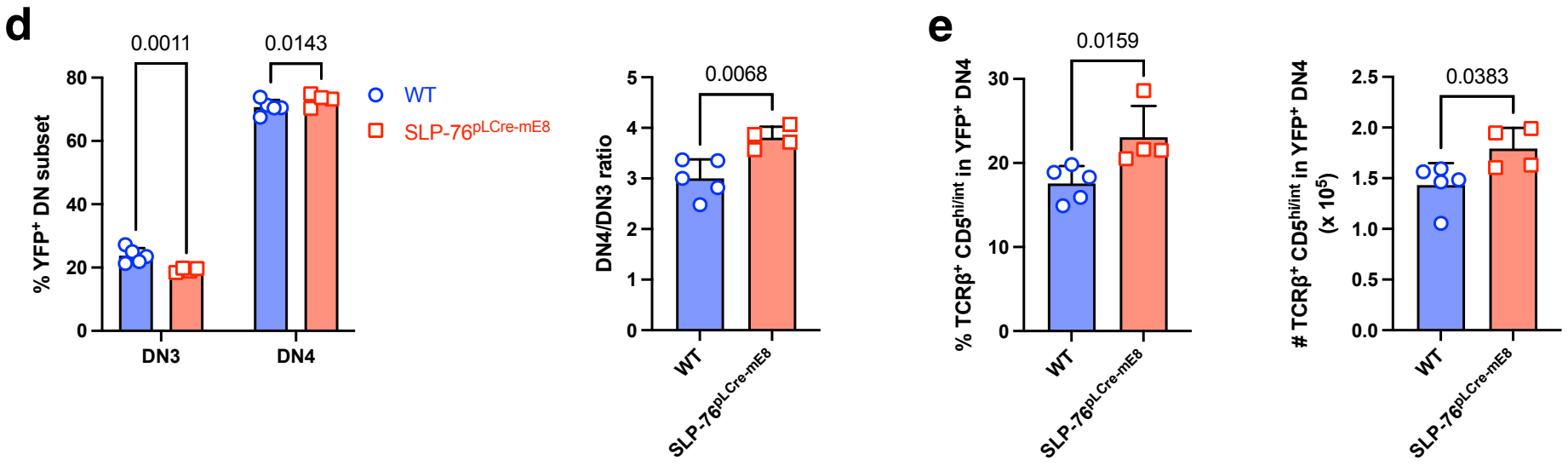

Supplementary Figure 3: FACS analysis of SLP-76pLCre-mE8 thymocytes for β-selection. **a**, Blood lymphocytes from *Rosa26LSL-eYFP* x *pLckCre* mice were analyzed for lineage-specific markers (CD3, CD19, and TCRβ) and YFP fate-mapping reporter. Shown are the representatives of T cell-specific (upper) and promiscuous (lower) expression of YFP. **b**, The number of WT and SLP-76pLCre-mE8 mice (at 8 – 10 weeks of age) with T cell-specific or promiscuous YFP expression. **c**, Gating strategy for analysis of WT and SLP-76pLCre-mE8 mice (at 9 – 10 weeks of age) for YFP+ CD4/CD8 double-negative (DN) thymocytes. **d**, Statistical analysis for the frequency (left) and ratio (right) of YFP+ DN3 (CD25+ CD44-) and DN4 (CD25- CD44-) thymocytes (n=5 for WT and n=4 for SLP-76pLCre-mE8). **e**, Statistical analysis for the frequency (left) and number (right) of TCRβ+ CD5hi/int DN4 thymocytes that have signaled through pre-TCR (n=5 for WT and n=4 for SLP-76pLCre-mE8). Each symbol represents one mouse. Data were shown as mean ± SD, and statistics was determined by two-way ANOVA (**d**: left) and Mann-Whitney test (**d**: right and **e**).

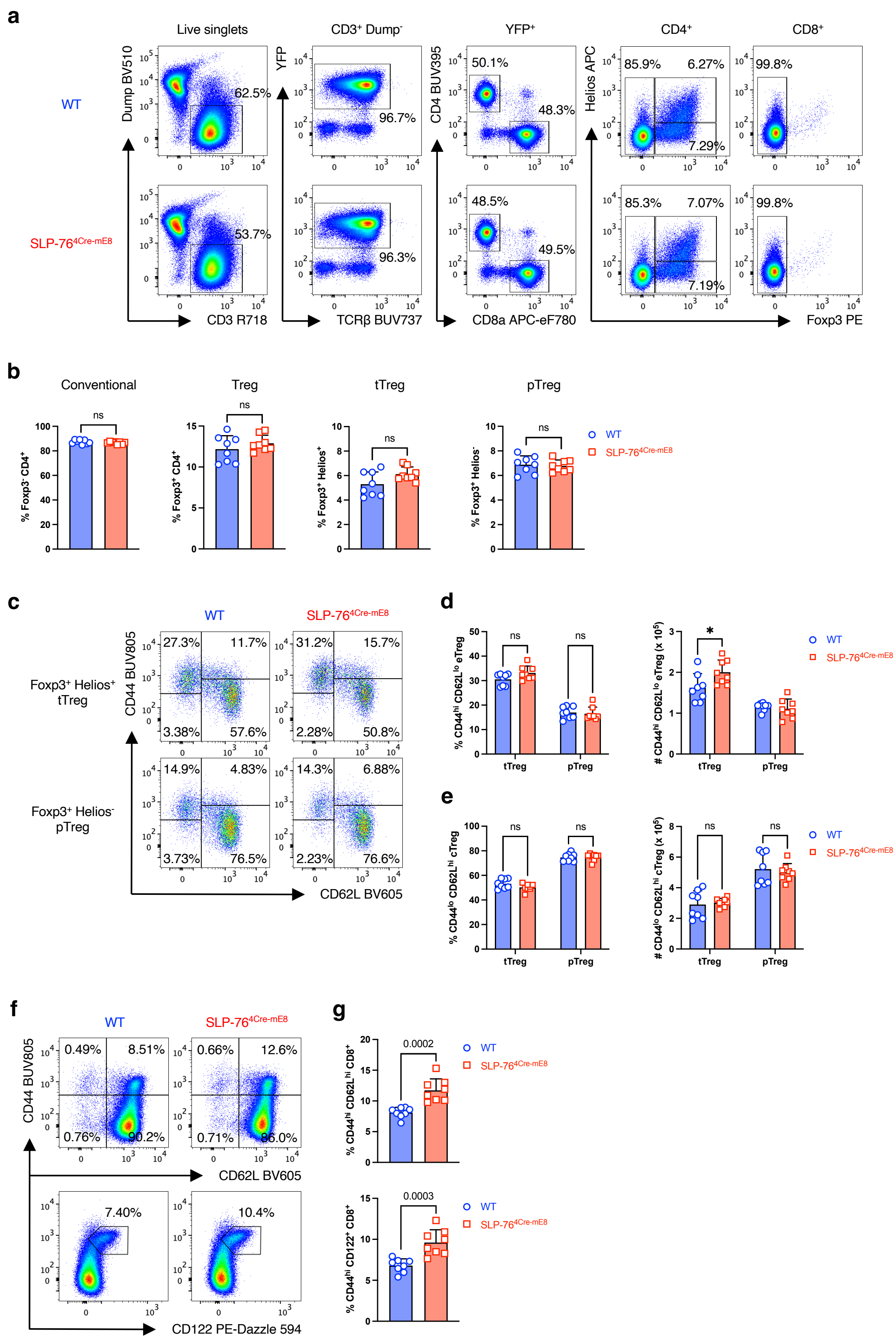

a
Live singlets
CD3+ Dump-
YFP+
CD4+
CD8+
WT
SLP-76 4Cre-mE8
Dump BV510
YFP
CD4 BUV395
Helios APC
62.5%
96.7%
50.1%
48.3%
85.9%
6.27%
7.29%
99.8%
53.7%
96.3%
48.5%
49.5%
85.3%
7.07%
7.19%
99.8%
CD3 R718
TCRβ BUV737
CD8a APC-eF780
Foxp3 PE
b
Conventional
Treg
tTreg
pTreg
% Foxp3- CD4+
% Foxp3+ CD4+
% Foxp3+ Helios+
% Foxp3+ Helios-
ns
WT
SLP-76 4Cre-mE8
c
WT
SLP-76 4Cre-mE8
Foxp3+ Helios+
tTreg
Foxp3+ Helios-
pTreg
CD44 BUV805
27.3%
11.7%
3.38%
57.6%
31.2%
15.7%
2.28%
50.8%
14.9%
4.83%
3.73%
76.5%
14.3%
6.88%
2.23%
76.6%
CD62L BV605
d
% CD44hi CD62Llo eTreg
# CD44hi CD62Llo eTreg (x 10^5)
tTreg
pTreg
*
e
% CD44lo CD62Lhi cTreg
# CD44lo CD62Lhi cTreg (x 10^5)
f
WT
SLP-76 4Cre-mE8
CD44 BUV805
0.49%
8.51%
0.76%
90.2%
0.66%
12.6%
0.71%
86.0%
CD62L BV605
7.40%
10.4%
CD122 PE-Dazzle 594
g
% CD44hi CD62Lhi CD8+
0.0002
% CD44hi CD122+ CD8+
0.0003

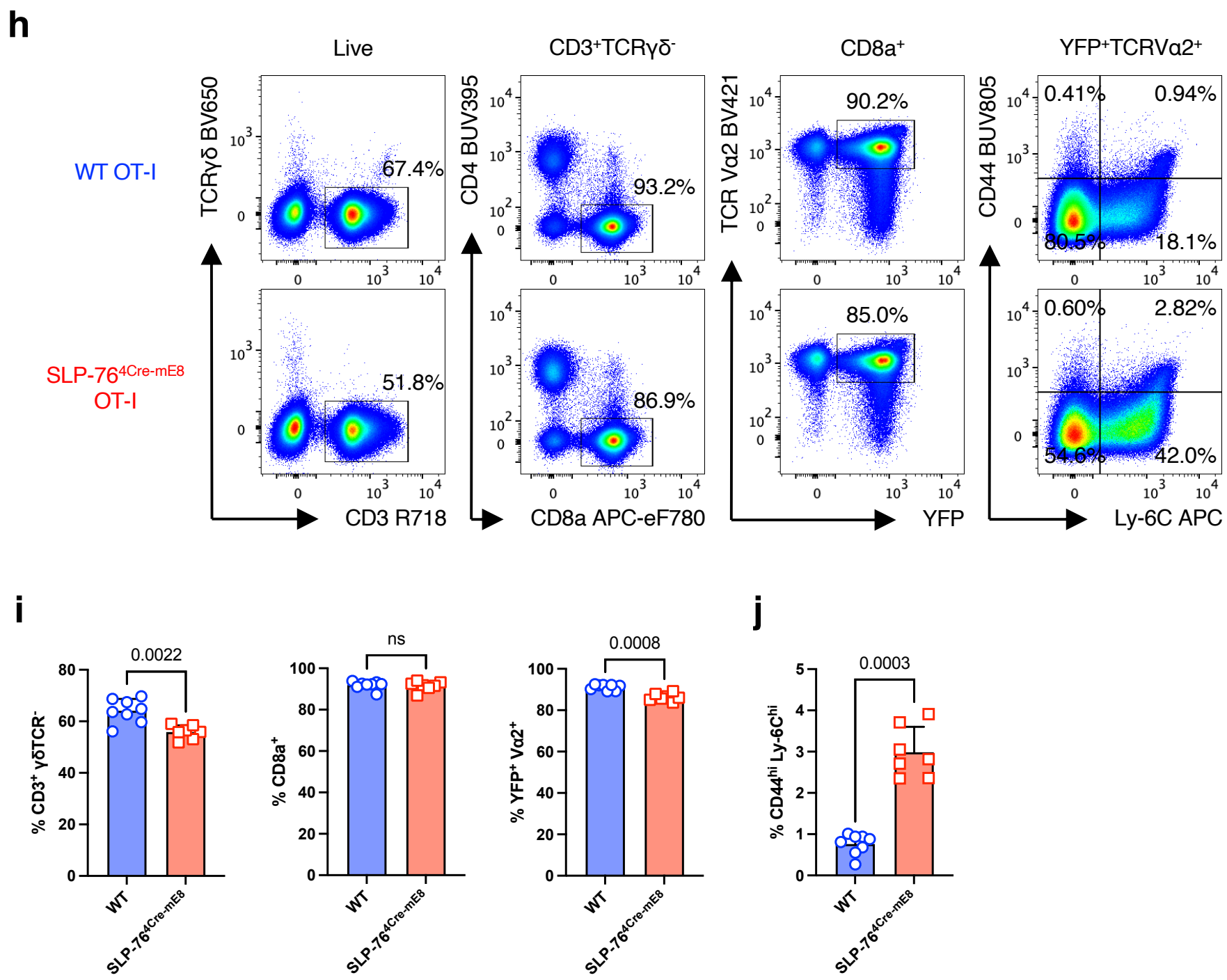

Supplementary Figure 4: FACS analysis of peripheral T cells in SLP-76$^{4Cre-mE8}$ mice. **a**, Gating strategy for analysis of YFP$^+$ CD4$^+$ and YFP$^+$ CD8$^+$ T cells in pooled whole LNs from WT (upper row) and SLP-76$^{4Cre-mE8}$ (lower row) mice. Dump channel contains CD19, I-A^b, NK1.1, and γδTCR. **b**, Statistical analysis of CD3$^+$ YFP$^+$ CD4$^+$ T cells for the frequency of conventional, total Tregs, tTregs, and pTregs (n=8 for each group). **c**, Representative pseudocolor plot analysis of tTregs and pTregs for CD44 and CD62L expression in WT and SLP-76$^{4Cre-mE8}$ mice. **d**,**e**, Statistical analysis of tTreg and pTreg compartments for the frequency (left) and number (right) of CD44^{high} CD62L^{low} eTregs (**d**) and CD44^{low} CD62L^{high} cTregs (**e**) (n=8 for each group). **f**,**g**, Representative pseudocolor plot analysis of CD3$^+$ YFP$^+$ Foxp3$^-$ conventional CD8$^+$ T cells from WT and SLP-76$^{4Cre-mE8}$ mice for the expression of CD62L and CD44 (upper row) and CD122 and CD44 (lower row) (**f**). Statistical analysis for the frequency of CD44^{high} CD62L^{high} (upper row) and CD44^{high} CD122$^+$ (lower row) CD8$^+$ T cells corresponding to CD8$^+$ T_{MP} cells (**g**) (n=8 for each group). **h**–**j**, Analysis of OT-I CD8$^+$ T cells in the periphery of WT (n=8) and SLP-76$^{4Cre-mE8}$ (n=7) OT-I mice. **h**, Representative pseudocolor plot analysis for CD8a$^+$ YFP$^+$ TCRVα2$^+$ CD44^{high} Ly-6C^{high} T_{MP} cells. **i**, Statistical analysis of the frequency of CD3$^+$ γδTCR$^-$ (left), CD8a$^+$ (middle), and YFP$^+$ TCRVα2$^+$ (right) cells. **j**, Statistical analysis of the frequency of a CD44^{high} Ly-6C^{high} population in CD8a$^+$ YFP$^+$ TCRVα2$^+$ T cells. Each symbol represents one mouse. Data were shown as mean ± SD, and statistics was determined by Mann-Whitney test (**b**,**g**,**i**,**j**) and two-way ANOVA (**d**,**e**). ns; not significant.

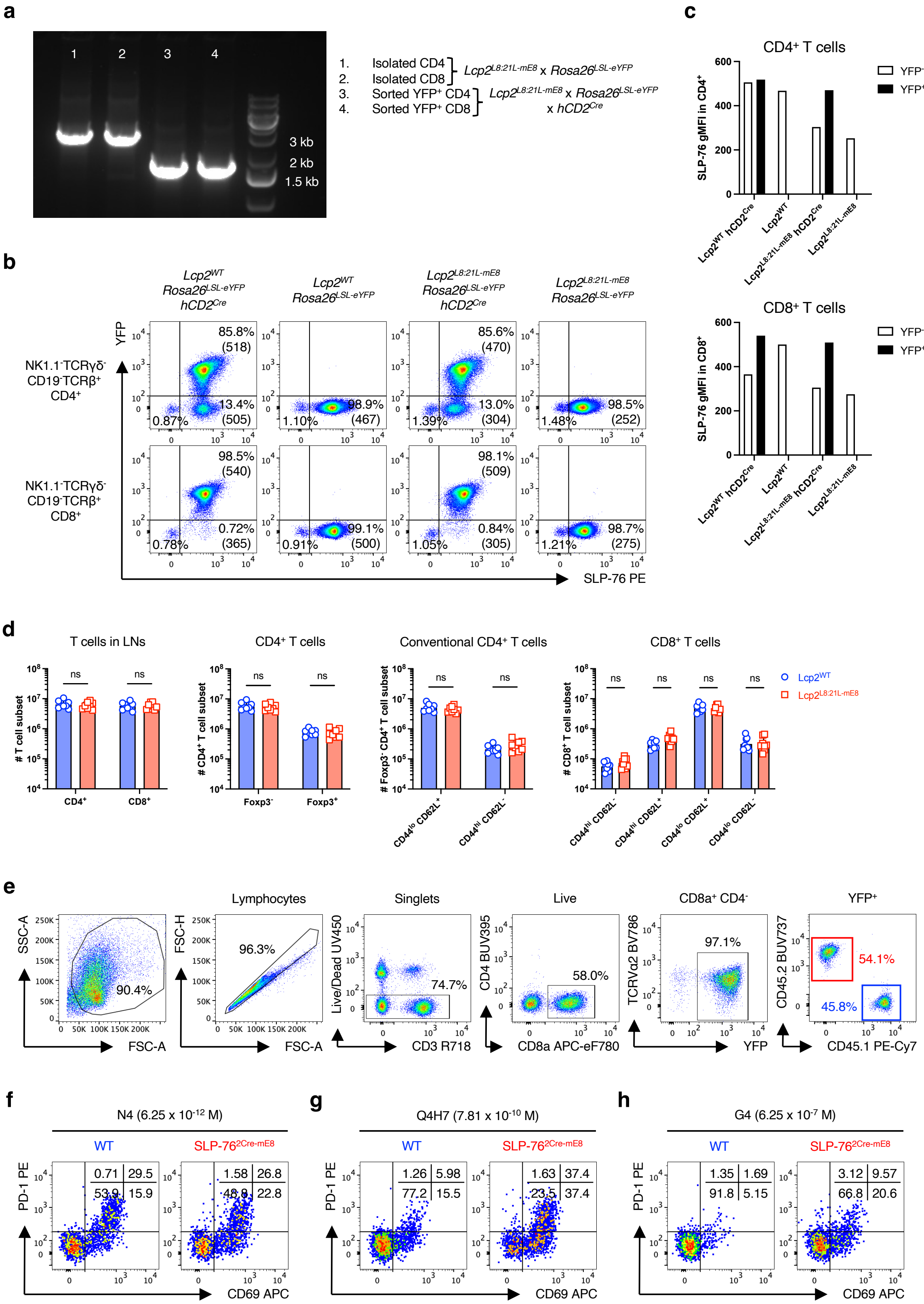

a
1 2 3 4
3 kb
2 kb
1.5 kb
1. Isolated CD4
2. Isolated CD8
Lcp2L8:21L-mE8 x Rosa26LSL-eYFP
3. Sorted YFP+ CD4
4. Sorted YFP+ CD8
Lcp2L8:21L-mE8 x Rosa26LSL-eYFP x hCD2Cre
b
Lcp2WT Rosa26LSL-eYFP hCD2Cre
Lcp2WT Rosa26LSL-eYFP
Lcp2L8:21L-mE8 Rosa26LSL-eYFP hCD2Cre
Lcp2L8:21L-mE8 Rosa26LSL-eYFP
NK1.1-TCRγδ- CD19-TCRβ+ CD4+
NK1.1-TCRγδ- CD19-TCRβ+ CD8+
YFP
SLP-76 PE
85.8% (518)
13.4% (505)
0.87%
1.10%
98.9% (467)
85.6% (470)
13.0% (304)
1.39%
1.48%
98.5% (252)
98.5% (540)
0.72% (365)
0.78%
0.91%
99.1% (500)
98.1% (509)
0.84% (305)
1.05%
1.21%
98.7% (275)
c
CD4+ T cells
CD8+ T cells
SLP-76 gMFI in CD4+
SLP-76 gMFI in CD8+
YFP-
YFP+
Lcp2WT hCD2Cre
Lcp2WT
Lcp2L8:21L-mE8 hCD2Cre
Lcp2L8:21L-mE8
d
T cells in LNs
CD4+ T cells
Conventional CD4+ T cells
CD8+ T cells
T cell subset
CD4+ T cell subset
Foxp3- CD4+ T cell subset
CD8+ T cell subset
ns
CD4+
CD8+
Foxp3-
Foxp3+
CD44lo CD62L+
CD44hi CD62L-
CD44hi CD62L+
Lcp2WT
Lcp2L8:21L-mE8
e
Lymphocytes
Singlets
Live
CD8a+ CD4-
YFP+
SSC-A
FSC-A
90.4%
FSC-H
96.3%
Live/Dead UV450
CD3 R718
74.7%
CD4 BUV395
CD8a APC-eF780
58.0%
TCRVα2 BV786
YFP
97.1%
CD45.2 BUV737
CD45.1 PE-Cy7
54.1%
45.8%
f
N4 (6.25 x 10-12 M)
WT
SLP-762Cre-mE8
PD-1 PE
CD69 APC
0.71 29.5
53.9 15.9
1.58 26.8
48.8 22.8
g
Q4H7 (7.81 x 10-10 M)
WT
SLP-762Cre-mE8
1.26 5.98
77.2 15.5
1.63 37.4
23.5 37.4
h
G4 (6.25 x 10-7 M)
WT
SLP-762Cre-mE8
1.35 1.69
91.8 5.15
3.12 9.57
66.8 20.6

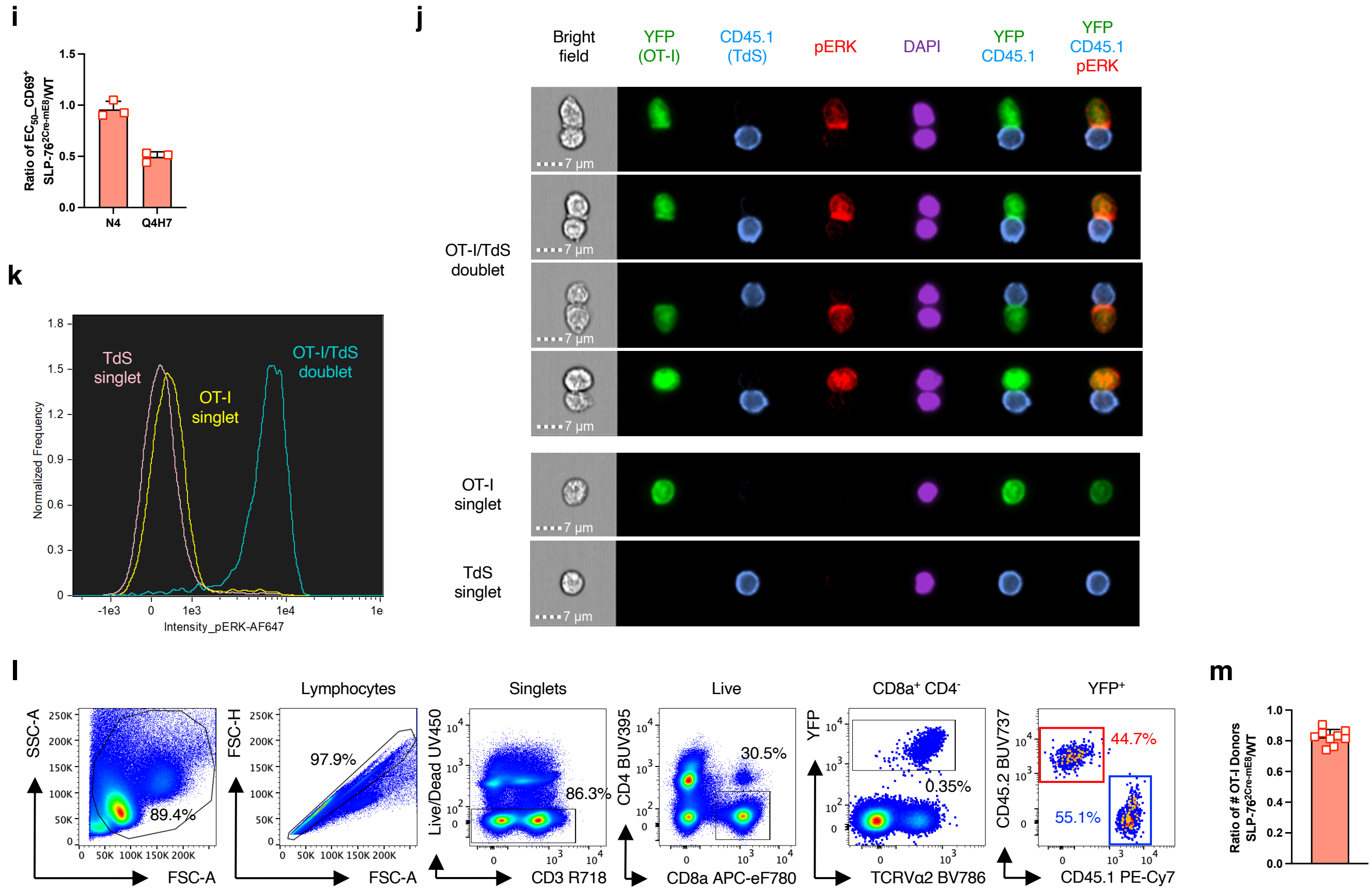

Supplementary Figure 5: Verification of hCD2^{Cre}-mediated SLP-76^{HA} mutation, FACS analysis of WT and SLP-76$^{2Cre\text{-}mE8}$ OT-I CD8^{+} T cells stimulated with peptide-loaded TdS cells, and identification of pERK^{+} cells by ImageStream.

a, Verification of a conditional SLP-76^{HA} mutation in CD4^{+} and CD8^{+} T cells isolated from Lcp2$^{L8:21L\text{-}mE8}$ x Rosa26$^{LSL\text{-}eYFP}$ mouse as well as sorted YFP^{+} CD4^{+} and YFP^{+} CD8^{+} T cells from Lcp2$^{L8:21L\text{-}mE8}$ x Rosa26$^{LSL\text{-}eYFP}$ x hCD2^{Cre} mouse by genomic PCR. **b**, Expression of SLP-76 and YFP in CD4^{+} T cells (upper row) and CD8^{+} T cells (lower row) of Lcp2^{WT} Rosa26$^{LSL\text{-}eYFP}$ and Lcp2$^{L8:21L\text{-}mE8}$ Rosa26$^{LSL\text{-}eYFP}$ mice with or without hCD2^{Cre}. Numbers in parentheses indicate a gMFI of SLP-76 in each quadrant. **c**, Bar graphs for gMFI of SLP-76 in CD4^{+} T cells and CD8^{+} T cells generated from the flowcytometric analysis (**b**). Shown is a representative data set from 3 independent experiments. **d**, Statistical analysis for the number of LN T cells (left), CD4^{+} T cells (middle left), Foxp3^{-} conventional CD4^{+} T cells (middle right), and CD8^{+} T cells (right) from Lcp2^{WT} Rosa26$^{LSL\text{-}eYFP}$ and Lcp2$^{L8:21L\text{-}mE8}$ Rosa26$^{LSL\text{-}eYFP}$ mice without Cre (n=8 for each group). **e**, Gating strategy for analysis of WT (CD45.1) and SLP-76$^{2Cre\text{-}mE8}$ (CD45.2) OT-I CD8^{+} T cells stimulated with peptide-loaded TdS cells. **f–h**, Representative pseudocolor plot analysis of WT and SLP-76$^{2Cre\text{-}mE8}$ OT-I CD8^{+} T cells for CD69 and PD-1 expression upon stimulation with 6.25 x 10^{-12} M N4 (**f**), 7.81 x 10^{-10} M Q4H7 (**g**), or 6.25 x 10^{-7} M G4 (**h**). Numbers indicate the frequency of cells in each quadrant. **i**, Fold change in EC_{50} of CD69^{+} between WT and SLP-76$^{2Cre\text{-}mE8}$ OT-I cells in response to N4 or Q4H7 peptide from 3 independent experiments. **j**,**k**, Identification of phospho-ERK (pERK)$^{+}$ cells in WT OT-I/N4-loaded TdS (APC) co-culture for 30 minutes by ImageStream. **j**, Representative images of OT-I/APC doublets, OT-I singlet, and APC singlet. **k**, Histograms for the level of pERK in OT-I/APC doublet, OT-I singlet, and APC singlet. **l**, Gating strategy for analysis of co-transferred WT CD45.1 and SLP-76$^{2Cre\text{-}mE8}$ CD45.2 OT-I CD8^{+} T cells in skin dLNs of (CD45.1 x CD45.2) F1 recipient mice (n=9) immunized with Q4H7 in CFA for 3 days. **m**, Ratio of SLP-76$^{2Cre\text{-}mE8}$ to WT OT-I donor cells. Each symbol represents one mouse. Data were shown as mean ± SD, and statistics was determined by two-way ANOVA (**d**). ns; not significant.

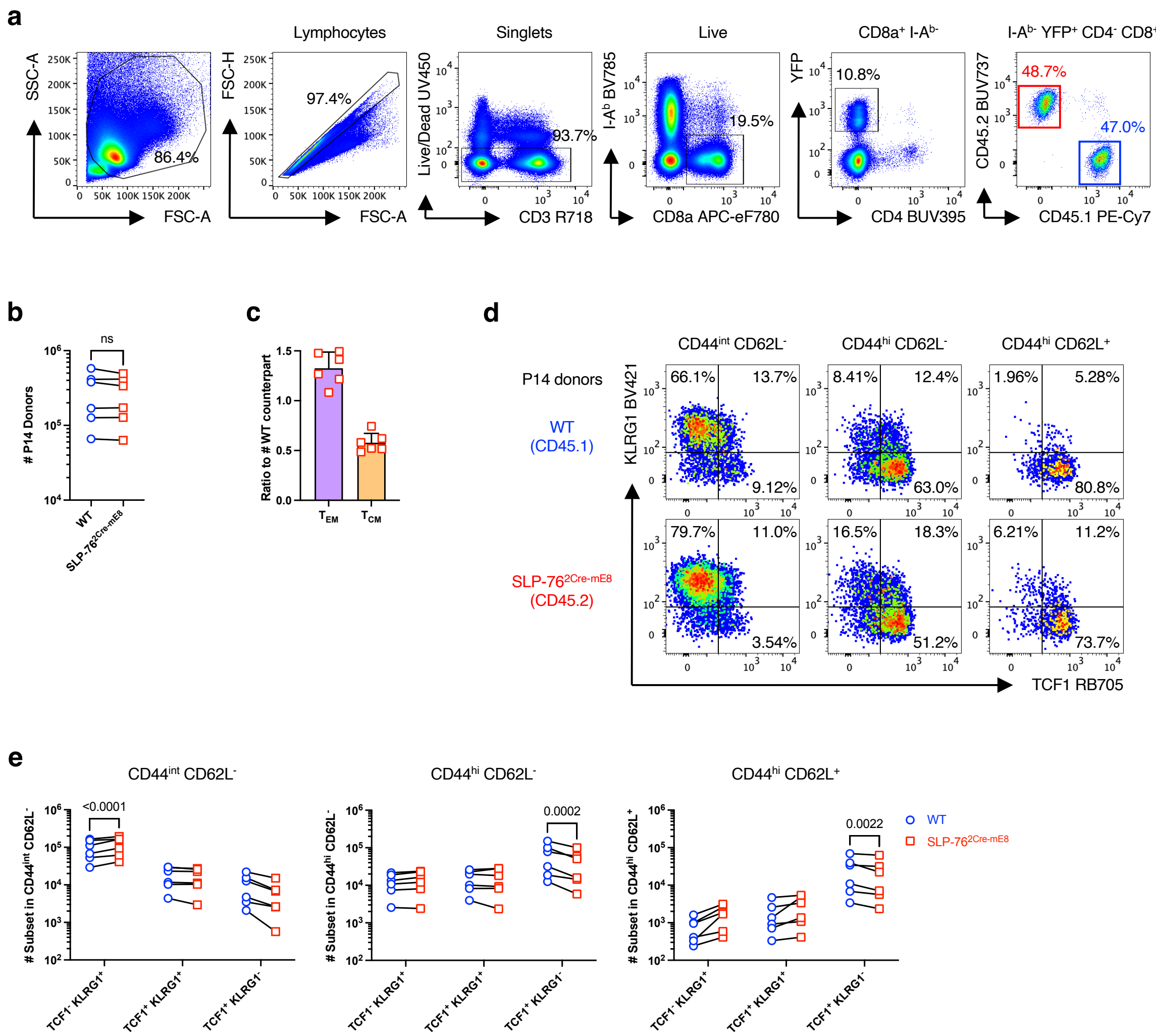

Supplementary Figure 6: FACS analysis of WT and SLP-76$^{2Cre\text{-}mE8}$ P14 CD8^{+} donor T cells in the memory phase of LCMV$_{Arm}$ infection.

a,**b**, Gating strategy for analysis of co-transferred WT CD45.1 and SLP-76$^{2Cre\text{-}mE8}$ CD45.2 P14 CD8^{+} T cells (**a**) and statistical analysis for the number of the two donor P14 cells (**b**) in the spleens of (CD45.1 x CD45.2) F1 recipient mice (n=6) at 28 days post-infection with LCMV$_{Arm}$. **c**, Ratio of SLP-76$^{2Cre\text{-}mE8}$ P14 T$_{EM}$ and T$_{CM}$ cells to WT P14 counterparts (n=6). **d**, Representative pseudocolor plot analysis of WT and SLP-76$^{2Cre\text{-}mE8}$ P14 donor cells for TCF1 and KLRG1 in three distinct subpopulations based on CD62L and CD44 expression. **e**, Statistical analysis for the number of TCF1^{-} KLRG1^{+}, TCF1^{+} KLRG1^{+}, and TCF1^{+} KLRG1^{-} cells within CD44^{int} CD62L^{-} (left), CD44^{hi} CD62L^{-} (middle), and CD44^{hi} CD62L^{+} (right) subpopulations (n=6). Symbols connected with a line indicate data from the same recipient mouse (**b**,**e**). Statistics was determined by Wilcoxon test (**b**) and two-way ANOVA (**e**).

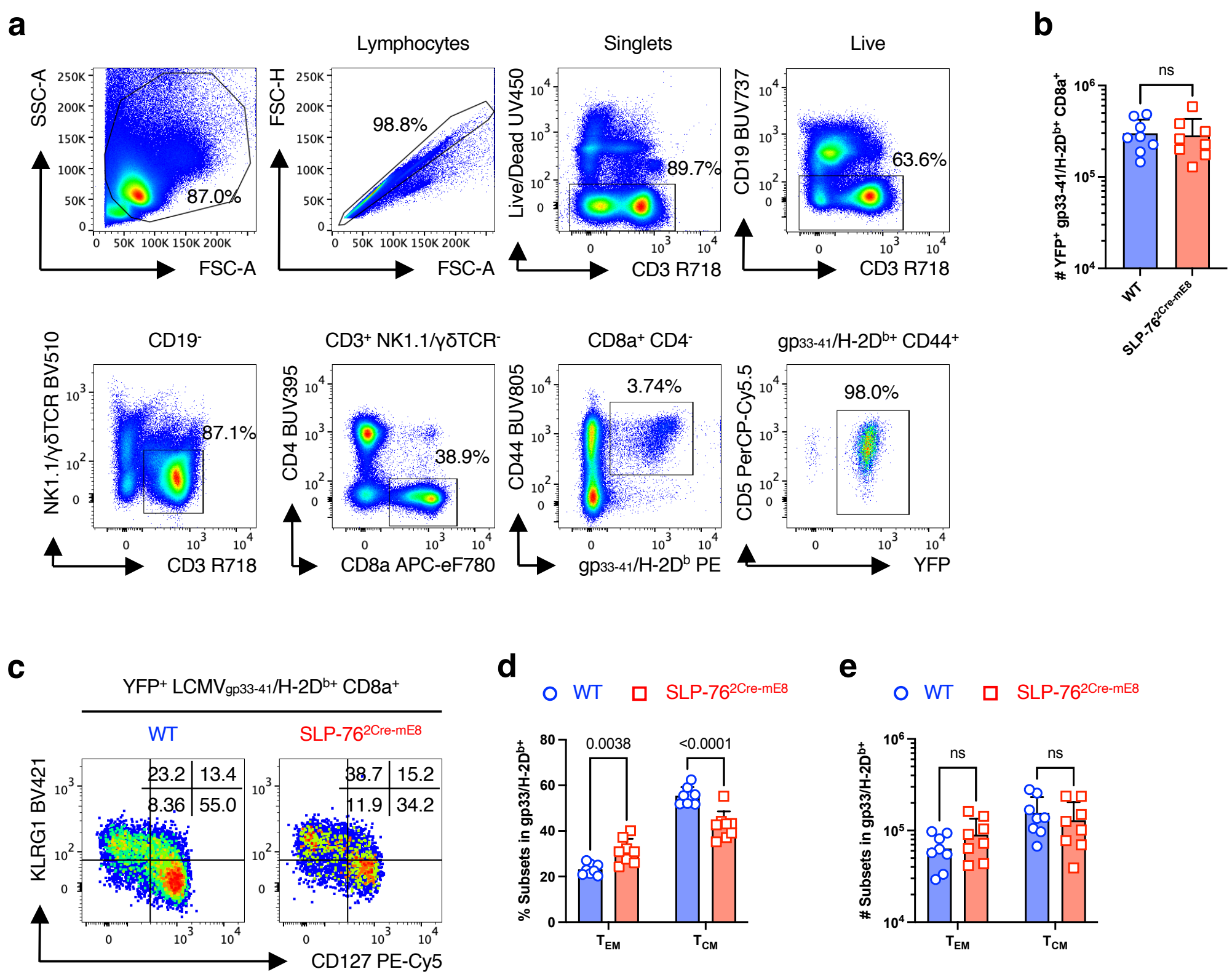

Supplementary Figure 7: FACS analysis of LCMV-reactive CD8+ T cells in $LCMV_{Arm}$-infected WT and SLP-76$^{2Cre\text{-}mE8}$ mice with endogenous TCR repertoire.

a,**b**, Gating strategy for analysis of $LCMV_{gp33\text{-}41}$-reactive CD8+ T cells in $LCMV_{Arm}$-infected WT mice (**a**) and statistical analysis for the number of YFP+ $LCMV_{gp33\text{-}41}$-reactive CD8+ T cells in WT (n=8) and SLP-76$^{2Cre\text{-}mE8}$ (n=8) mice (**b**) at 30 days post-infection with $LCMV_{Arm}$. **c**, Representative pseudocolor plot analysis of YFP+ $LCMV_{gp33\text{-}41}$/H-2D^b-tetramer+ CD8+ T cells for CD127 and KLRG1 expression at 30 days post-infection with $LCMV_{Arm}$. Numbers indicate the frequency of cells in each quadrant. **d**,**e**, Statistical analysis of $gp_{33\text{-}41}$/H-2D^b-tetramer+ CD8+ T cells for the frequency (**d**) and number (**e**) of T_{EM} (CD127- KLRG1+) and T_{CM} (CD127+ KLRG1-) cells (n=8 for each group). Each symbol represents one mouse (**b**,**d**,**e**). Data were shown as mean ± SD, and statistics was determined by Mann-Whitney test (**b**), and two-way ANOVA (**d**,**e**). ns; not significant.

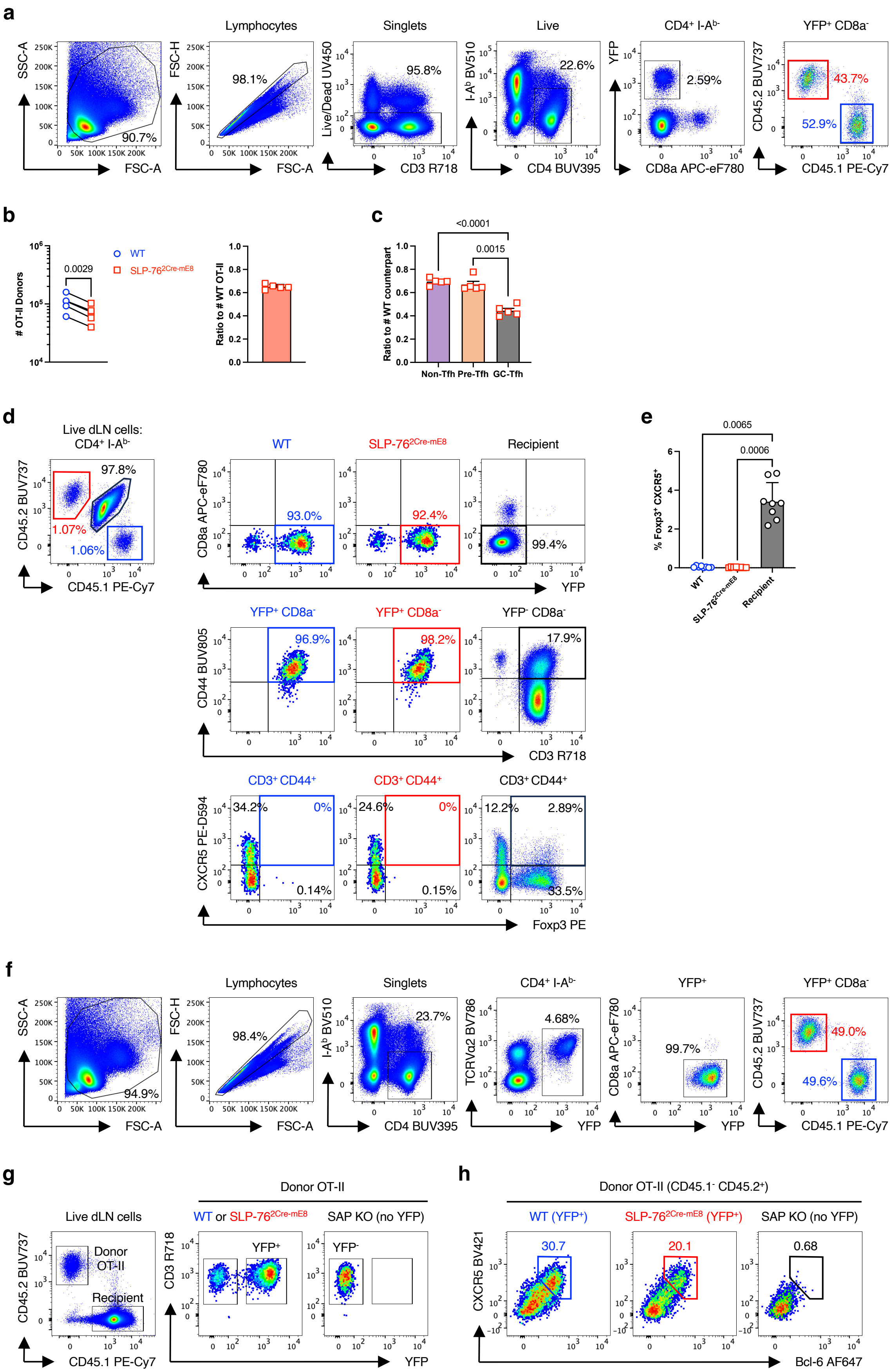

a
Lymphocytes
Singlets
Live
CD4+ I-Ab-
YFP+ CD8a-
SSC-A
FSC-A
90.7%
FSC-H
98.1%
Live/Dead UV450
95.8%
CD3 R718
I-Ab BV510
22.6%
CD4 BUV395
YFP
2.59%
CD8a APC-eF780
CD45.2 BUV737
43.7%
52.9%
CD45.1 PE-Cy7
b
OT-II Donors
0.0029
WT
SLP-76 2Cre-mE8
Ratio to # WT OT-II
c
<0.0001
0.0015
Ratio to # WT counterpart
Non-Tfh
Pre-Tfh
GC-Tfh
d
Live dLN cells: CD4+ I-Ab-
97.8%
1.07%
1.06%
CD45.2 BUV737
CD45.1 PE-Cy7
WT
SLP-76 2Cre-mE8
Recipient
CD8a APC-eF780
93.0%
92.4%
99.4%
YFP
YFP+ CD8a-
YFP+ CD8a-
YFP- CD8a-
CD44 BUV805
96.9%
98.2%
17.9%
CD3 R718
CD3+ CD44+
CD3+ CD44+
CD3+ CD44+
CXCR5 PE-D594
34.2%
0%
0.14%
24.6%
0%
0.15%
12.2%
2.89%
33.5%
Foxp3 PE
e
0.0065
0.0006
% Foxp3+ CXCR5+
WT
SLP-76 2Cre-mE8
Recipient
f
Lymphocytes
Singlets
CD4+ I-Ab-
YFP+
YFP+ CD8a-
SSC-A
94.9%
FSC-A
FSC-H
98.4%
FSC-A
I-Ab BV510
23.7%
CD4 BUV395
TCRVα2 BV786
4.68%
YFP
CD8a APC-eF780
99.7%
YFP
CD45.2 BUV737
49.0%
49.6%
CD45.1 PE-Cy7
g
Live dLN cells
Donor OT-II
Recipient
CD45.2 BUV737
CD45.1 PE-Cy7
Donor OT-II
WT or SLP-76 2Cre-mE8
SAP KO (no YFP)
CD3 R718
YFP+
YFP-
YFP
h
Donor OT-II (CD45.1- CD45.2+)
WT (YFP+)
SLP-76 2Cre-mE8 (YFP+)
SAP KO (no YFP)
CXCR5 BV421
30.7
20.1
0.68
Bcl-6 AF647

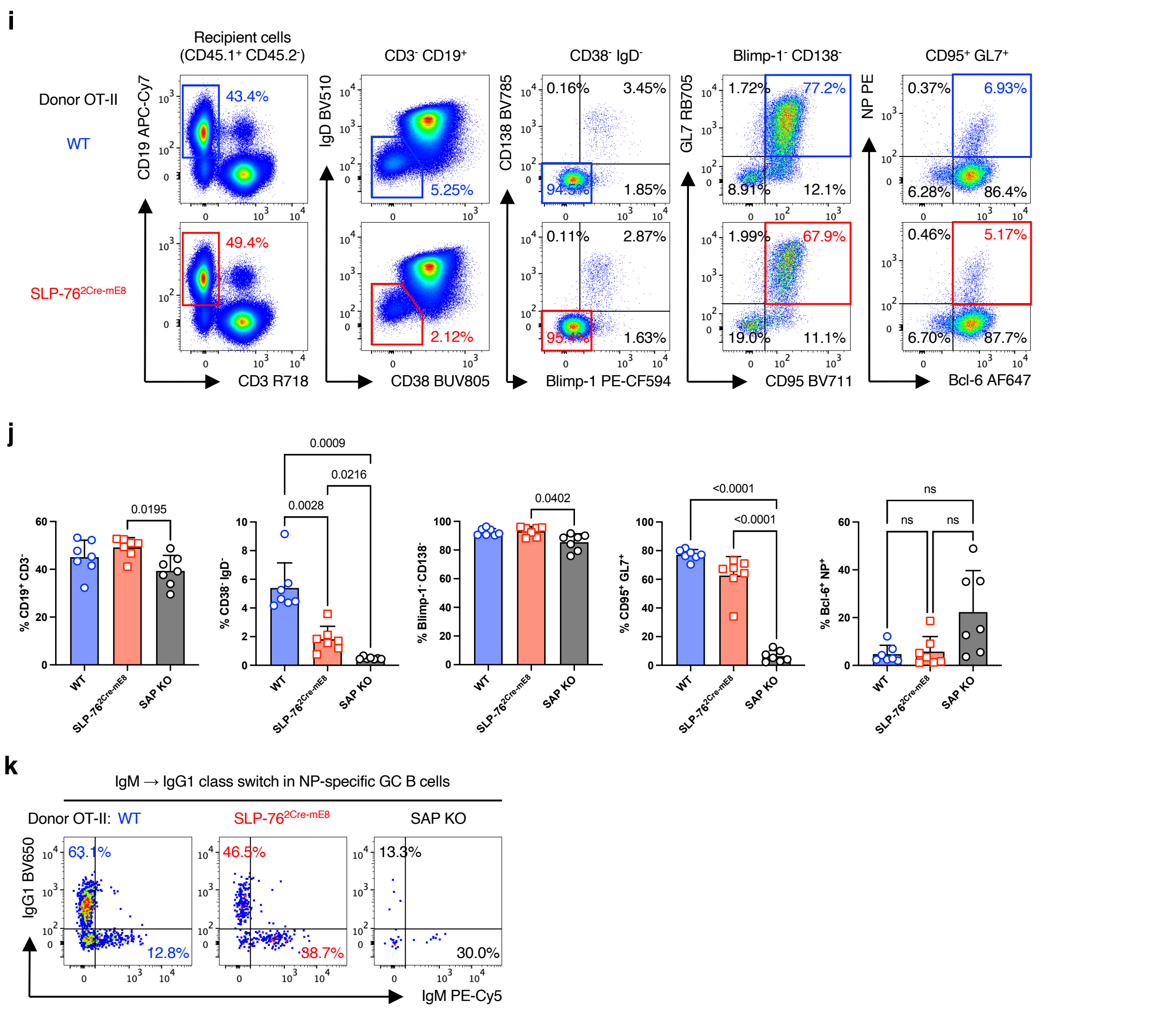

Supplementary Figure 8: FACS analysis of donor OT-II CD4$^+$ T cells for Tfh cells, dead cells in a CXCR5$^+$ population, and germinal center B cells.

a, Gating strategy for analysis of co-transferred WT CD45.1 and SLP-76$^{2Cre\text{-}mE8}$ CD45.2 OT-II CD4$^+$ T cells in skin dLNs of (CD45.1 x CD45.2) F1 recipient mice (n=5 for each group) at 10 days post-immunization with OVA in CFA. **b**, Statistical analysis for the number of WT and SLP-76$^{2Cre\text{-}mE8}$ OT-II donor cells (left) and the ratio of SLP-76$^{2Cre\text{-}mE8}$ to WT OT-II donor cells (right). Symbols connected with a line indicate data from the same recipient mouse. **c**, Ratio of SLP-76$^{2Cre\text{-}mE8}$ non-Tfh, pre-Tfh, and GC-Tfh cells to WT OT-II counterparts. **d**, Gating strategy for analyzing WT OT-II (CD45.1$^+$), SLP-76$^{2Cre\text{-}mE8}$ OT-II (CD45.2$^+$) donor cells, and recipient-derived CD4$^+$ T cells (CD45.1$^+$ CD45.2$^+$) for Tfr cells. **e**, Statistical analysis for the frequency of Foxp3$^+$ CXCR5$^+$ Tfr cells in antigen-primed (CD44^{high}) WT and SLP-76$^{2Cre\text{-}mE8}$ OT-II donor cells as well as recipient-derived CD4$^+$ T cells (n=8 for each group). **f**, Gating strategy to analyze dead cells in CXCR5$^-$ and CXCR5$^+$ populations at 6 days post-immunization with OVA in CFA. **g**,**h**, Gating strategy for identification (**g**) and characterization (**h**) of WT, SLP-76$^{2Cre\text{-}mE8}$, and SAP-KO OT-II donor cells in recipient SAP KO mice immunized with NP-OVA in CFA. **i**, Gating strategy for identification of germinal center (GC) B cells (total and NP-specific) in NP-OVA-immunized SAP KO mice that have received WT or SLP-76$^{2Cre\text{-}mE8}$ OT-II CD4$^+$ T cells. **j**, Statistical analysis for the frequency of total B cells (CD19$^+$ CD3$^-$), activated B cells (CD38$^-$ IgD$^-$), non-plasma cells (Blimp-1$^-$ CD138$^-$), total (CD95$^+$ GL7$^+$) and NP-specific (NP$^+$ Bcl-6$^+$) GC B cells (n=7 for each group). **k**, Representative pseudocolor plot analysis for IgM → IgG1 class switch in NP-specific GC B cells of SAP KO mice that have received WT, SLP-76$^{2Cre\text{-}mE8}$, or SAP KO OT-II cells. Each symbol represents one mouse. Data were shown as mean ± SD, and statistics was determined by Wilcoxon test (**b**) and Kruskal-Wallis test (**c**,**e**,**j**). ns; not significant.

Interactions	Sequence of SLP-76	K_d (nM)	Temp.(˚C)	ΔH (kcal/mol)	−TΔS (kcal/mol)	ΔG (kcal/mol)
SLP-76 / PLC-γ1	Wild type	2360	5	-1.5	-5.7	-7.2
	High affinity	1000	5	-11.2	3.6	-7.6
	No affinity	ND	5	ND	ND	ND
SLP-76 / Itk	Wild type	ND	5	ND	ND	ND
	High affinity	ND	5	ND	ND	ND
	No affinity	ND	5	ND	ND	ND

Supplementary Table 1: Thermodynamic parameters of the interaction of SLP-76 with PLC-γ1 or Itk.
