# Supplementary material for: Weak SLP-76/PLC-γ1 interaction fine-tunes T cell receptor signal strength to optimize T cell responsiveness
Source: Nat Commun. 2026 Jul 29;17:9189. doi: 10.1038/s41467-026-75599-0 (PMC13522589; doi:10.1038/s41467-026-75599-0)
Supplement: Supplementary file 2 [file 41467_2026_75599_MOESM2_ESM.pdf]

nature portfolio

Peer Review File

Weak SLP-76/PLC-γ1 interaction fine-tunes T cell receptor signal strength to optimize T cell responsiveness

Corresponding Author: Dr Hidehiro Yamane

This file contains all reviewer reports in order by version, followed by all author rebuttals in order by version.

Version 0:

Reviewer comments:

Reviewer #1

(Remarks to the Author)
After phosphorylation by ZAP-70, the four carboxy-terminal tyrosine residues of the LAT adaptor molecules recruit SH2-domain containing proteins, giving rise to a quaternary subcomplex consisting of LAT, phospholipase C-g1 (PLC-g1) and of the GADS and SLP-76 (LCP2) adaptors. PLC-g1 directly binds to a phosphorylated LAT tyrosine residue via its amino-terminal SH2 domain and to the inner face of the plasma membrane via its pleckstrin-homology domain (PH), whereas SLP-76 which is constitutively associated with GADS via one of the GADS SH3 domain is recruited to phosphorylated LAT tyrosine residues via the GADS SH2 domain. In former work, the team of L. Samelson has reconstituted part of the LAT in vitro. It permitted to determine the stoichiometry of an in vitro reconstituted quaternary complex containing LAT, GADS, SLP-76, and PLC-γ1 and to measure the thermodynamic forces driving its assembly. In the present study, they focus on the weak, evolutionary conserved interaction that occurs within this heterotetrameric quaternary complex between SLP-76 and PLC-γ1 and involves binding sites that are highly conserved in vertebrates. To figure out whether this weak interaction might serve as a critical check point for TCR-mediated T cell activation, they introduced a mutation (SLP-76HA) in the relevant region of SLP-76 (PRR 185-200) that greatly enhanced its affinity for the PLC-γ1 SH3 domain. Importantly, the SLP-76HA mutation was not responsible for the creation of de novo binding site for the SH3 domain of ITK or LCK. As expected, this higher affinity interaction increased the activity of PLC-γ1 in vitro. Mice expressing in a conditional manner an Lcp2 allele coding for the SLP-76NA mutation were developed. To verify that the excision of the LoxP-flanked SLP-76 cDNA Ex. 8-21/poly A sequence occurred appropriately, the resulting mice were crossed to Rosa26LSL-YFP reporter mice, allowing to track cells expressing the mutated SLP-76-expressing cells via YFP expression. To specifically analyze the effect of the SLP-76HA mutation on T cell development the above mice were crossed onto CD4Cre mice. SLP-76 CD4 Cre-mE8 YFP+ DP thymocytes showed a slight decrease in the frequency of post-positive selection TCRβhighCD5int DP3 cells and such DP3 thymocytes showed a higher frequency of cleaved Caspase-3-positive cells, suggesting that the SLP-76HA mutation promotes the apoptosis of DP thymocytes that normally survive after positive selection. Analysing caspase-3-expression on CD4 and CD8 SP thymocytes that were subdivided on the basis of CCR7 expression suggested that the SLP-76HA mutation promoted the apoptosis of CD4 and CD8 SP thymocytes that had passed the negative selection step and respond to self-peptide/MHC complexes. The conventional CD4 T cells, tTregs and pTregs found in the periphery were barely affected. The only notable feature was the presence of increased numbers of TMP cells.
To analyse the influence of the SLP-76HA mutation on the activation of peripheral T cells, the Lcp2L8:21L-mE8 x R26R-EYFP mice were crossed onto hCD2Cre and on OT-I TCR transgenic mice. Control R26R-EYFP x hCD2Cre x OT-I (WT OT-I) mice were also established. Both types of mice were bred on a CD45.1 and CD45.2 background, respectively, to distinguish OT-I T cells originating from the two different genotypes. Using in vitro stimulation with strong and weak agonistic peptides, the SLP-76HA mutation was found without effect on the responsiveness of OT-I T cells to a strong agonist peptide but increased their responsiveness to the G4 weak agonist. Carefully controlled in vivo experiments relying on adoptive transfer and relying on the Q4H7 'intermediate' agonist showed that SLP-76HA mutation increased OT-I T cell proliferation and coincidently rendered the proliferating T cells more apoptotic. Using similar adoptive transfer model and the LCMV-specific P14 TCR, in lieu of OT-I, the authors noted that the SLP-76HA mutation only affected the generation of TCM cells in the early memory phase of the response to the LCMV Armstrong strain. Finally, the authors analyzed the effect of the SLP-76HA mutation on Tfh development using their adoptive transfer model and the OT-II TCR and found that the SLP-76HA mutation promoted death of pre-Tfh cells during cognate interaction with B cells (see specific comment 4). This very thorough study combines a large body of sophisticated approaches to determine in a convincing manner the effect of SLP-76HA mutation on thymocyte development and peripheral T cell responses. As discussed by the authors, it is very puzzling that the SLP-76HA mutation has such a modest effect when compared with that resulting from the LATG135D mutation.

Specific comments.
1/ Several datasets are expressed as % of YFP+ cells (see Figure 2B). In that context, it might be useful to mention the efficiency of deletion in the various conditions reached using CD4-Cre? In the case the deletion is not substantial, the undeleted WT T cells may have outcompete the apoptotic SLP-76HA expressing T cells. The same applies to the very sophisticated crosses involving the hCD2-Cre.
2/ In the case OT-I mice are crossed on CD4-cre, does the deletion of the LoxP-flanked SLP-76 cDNA Ex. 8-21/poly A cassette affects the early steps of OT-I T cell development in the thymus?
3/ Can the authors provide references allowing them to qualify the 'PD-1intCXCR5int' cells as pre-Tfh cells ?
4/ Have the authors determined the effect of the SLP-76HA mutation on T follicular regulatory cells?
5/ Is it possible that SLP-76HA and LATG135D mutations differently affect TCR repertoire?
6/ Has the SLP-76HA mutation an effect on CD4+ iNKT cells ?
7/ SLP-76 is expressed several cell types (https://rstats.immgen.org/Skyline/skyline.html) in addition to T cells and important for their functions. Using appropriate Cre mice, it will be great to document in further studies the effect of the SLP76-HA mutation on platelets?

Reviewer #2

(Remarks to the Author)
This paper follows up on earlier work by this group identifying a weak interaction between SLP-76 and PLCγ1 that bridges the pLAT–Gads–SLP-76 and pLAT–PLCγ1 complexes forming the pLAT signalosome downstream of TCR stimulation. The biophysical basis of these interactions is well established, and the first part of this manuscript strengthens those findings by showing that the interaction site between SLP-76 and PLCγ1 is highly conserved and that increasing the affinity of this binding (SLP-76^HA) enhances the interaction and leads to stronger PLCγ1 enzymatic activity.
The authors further generated transgenic mice in which SLP-76^HA is conditionally expressed under Cre control. By crossing these mice with different Cre strains and employing various immune models, they provide a broad view of the phenotypes of T cells expressing SLP-76^HA across different developmental stages and functional contexts. They report that enhancing SLP-76's affinity for PLCγ1 promotes apoptosis of post-selection thymocytes, skews peripheral $CD8^+$ T cells toward a memory phenotype under steady-state conditions, increases responsiveness of antigen-specific T cells to weak peptide–MHC complexes, elevates short-lived effector cells (SLECs) during acute LCMV infection while reducing stem-like memory cells, and decreases TFH formation following OVA–CFA immunization.
Overall, the manuscript is clearly written, and the experiments are competently performed. However, several issues merit attention before the paper can be considered for publication.
Major Comments
1. The conditional expression strategy of SLP-76HA is clever, but the authors should show that the "Lcp2L8:21L–mE8" designation does not alter basal SLP-76 expression in thymocyte or peripheral T cell subsets. Protein-level quantification (e.g., immunoblot or flow cytometry) of SLP-76 in these subsets from Lcp2mE8 mice following Cre recombination is recommended.
2. The authors claim enhanced negative selection based on increased $CC3^+$ post-selection thymocytes. If so, why does this not lead to a reduction in total mature $CD4^+$ or $CD8^+$ SP thymocytes or peripheral T cell numbers? This discrepancy should be addressed experimentally or discussed.
3. The data show more SLECs in SLP-76^2Cre–mE8 P14 cells at day 7 post LCMV-Armstrong infection. Would this translate into faster viral clearance or altered secondary responses upon reinfection? Experimental data supporting these implications would strengthen the claims.
4. The authors should clarify the classification of $CD127^-KLRG1^+$, $CD127^+KLRG1^-$, and $TCF1^+KLRG1^-$ populations as SLECs, MPECs, and stem-like memory cells, respectively, rather than using TEM/TCM nomenclature, which applies to CD44/CD62L-based definitions (Kaech et al., Cell 2003; Kaech & Cui, Nat Rev Immunol 2012).
5. The reduced TFH population in SLP-76^2Cre–mE8 OT-II mice raises the question of whether this defect affects germinal center B cell formation or antigen-specific antibody titers. Experimental validation would be helpful.
6. The central mechanistic claim is that SLP-76^HA enhances TCR signaling, yet no direct signaling data are shown. The authors should measure pERK1/2, Nur77 expression, Ca^{2+} flux, or NFAT/NF-κB activation in mutant versus WT cells to substantiate this assertion.
7. Since SLP-76 is essential for pre-TCR signaling, it would be highly informative to test whether the SLP-76NA mutant (which cannot bind PLCγ1) supports thymocyte development beyond the DN3 stage. Comparison between SLP-76 knockout and SLP-76NA mice would add valuable mechanistic insight.
8. The authors note that the SLP-76^HA mutation preferentially enhances responses to weak agonists. It would be useful to analyze other "agonist-selected" T cells, such as NKT or $CD8\alpha\alpha^+$ intraepithelial lymphocytes, to test this model more broadly.

This study provides an elegant genetic approach to modulate the SLP-76–PLCγ1 interaction and explores its impact across multiple T cell contexts. However, without direct signaling data and deeper mechanistic analysis, the functional consequences of enhanced SLP-76–PLCγ1 binding appear limited, which may constrain the implications of these findings for future work.

Reviewer #3

(Remarks to the Author)
I co-reviewed this manuscript with one of the reviewers who provided the listed reports. This is part of the Nature Communications initiative to facilitate training in peer review and to provide appropriate recognition for Early Career Researchers who co-review manuscripts.

Version 1:

Reviewer comments:

Reviewer #1

(Remarks to the Author)
None

Reviewer #2

(Remarks to the Author)
The authors have done an excellent job in responding to the previous critiques.
There is one minor concern regarding the statistical significance reported in Fig. 7i, left panel, for SLP76^2Cre-Em8 vs. SAP KO. The differences in the bar graphs do not appear to be consistent with “ns”. At least to my eyes, this seems more likely to be significant at least the two-star level.

Reviewer #3

(Remarks to the Author)
I co-reviewed this manuscript with one of the reviewers who provided the listed reports. This is part of the Nature Communications initiative to facilitate training in peer review and to provide appropriate recognition for Early Career Researchers who co-review manuscripts.

Open Access This Peer Review File is licensed under a Creative Commons Attribution 4.0 International License, which permits use, sharing, adaptation, distribution and reproduction in any medium or format, as long as you give appropriate credit to the original author(s) and the source, provide a link to the Creative Commons license, and indicate if changes were made.
In cases where reviewers are anonymous, credit should be given to 'Anonymous Referee' and the source.
The images or other third party material in this Peer Review File are included in the article’s Creative Commons license, unless indicated otherwise in a credit line to the material. If material is not included in the article’s Creative Commons license and your intended use is not permitted by statutory regulation or exceeds the permitted use, you will need to obtain permission directly from the copyright holder.
To view a copy of this license, visit https://creativecommons.org/licenses/by/4.0/

Reviewer #1 (Remarks to the Author):

After phosphorylation by ZAP-70, the four carboxy-terminal tyrosine residues of the LAT adaptor molecules recruit SH2-domain containing proteins, giving rise to a quaternary subcomplex consisting of LAT, phospholipase C-γ1 (PLC-γ1) and of the GADS and SLP-76 (LCP2) adaptors. PLC-γ1 directly binds to a phosphorylated LAT tyrosine residue via its amino-terminal SH2 domain and to the inner face of the plasma membrane via its pleckstrin-homology domain (PH), whereas SLP-76 which is constitutively associated with GADS via one of the GADS SH3 domain is recruited to phosphorylated LAT tyrosine residues via the GADS SH2 domain. In former work, the team of L. Samelson has reconstituted part of the LAT in vitro. It permitted to determine the stoichiometry of an in vitro reconstituted quaternary complex containing LAT, GADS, SLP-76, and PLC-γ1 and to measure the thermodynamic forces driving its assembly. In the present study, they focus on the weak, evolutionary conserved interaction that occurs within this heterotetrameric quaternary complex between SLP-76 and PLC-γ1 and involves binding sites that are highly conserved in vertebrates. To figure out whether this weak interaction might serve as a critical check point for TCR-mediated T cell activation, they introduced a mutation (SLP-76^{HA}) in the relevant region of SLP-76 ($PRR_{185-200}$) that greatly enhanced its affinity for the PLC-γ1 SH3 domain. Importantly, the SLP-76^{HA} mutation was not responsible for the creation of de novo binding site for the SH3 domain of ITK or LCK. As expected, this higher affinity interaction increased the activity of PLC-γ1 in vitro. Mice expressing in a conditional manner an Lcp2 allele coding for the SLP-76NA mutation were developed. To verify that the excision of the LoxP-flanked SLP-76 cDNA Ex. 8-21/poly A sequence occurred appropriately, the resulting mice were crossed to $Rosa26^{LSL\text{-}YFP}$ reporter mice, allowing to track cells expressing the mutated SLP-76-expressing cells via YFP expression. To specifically analyze the effect of the SLP-76^{HA} mutation on T cell development the above mice were crossed onto $CD4^{Cre}$ mice. SLP-$76^{CD4Cre\text{-}mE8}$ YFP^{+} DP thymocytes showed a slight decrease in the frequency of post-positive selection $TCR\beta^{high}$ $CD5^{int}$ DP3 cells and such DP3 thymocytes showed a higher frequency of cleaved Caspase-3-positive cells, suggesting that the SLP-76^{HA} mutation promotes the apoptosis of DP thymocytes that normally survive after positive selection. Analysing caspase-3-expression on CD4 and CD8 SP thymocytes that were subdivided on the basis of CCR7 expression suggested that the SLP-76^{HA} mutation promoted the apoptosis of CD4 and CD8 SP thymocytes that had passed the negative selection step and respond to self-peptide/MHC complexes. The conventional CD4 T cells, tTregs and pTregs found in the periphery were barely affected. The only notable feature was the presence of increased numbers of T_{MP} cells.

To analyse the influence of the SLP-76^{HA} mutation on the activation of peripheral T cells, the $Lcp2^{L8:21L\text{-}mE8}$ x R26R-EYFP mice were crossed onto $hCD2^{Cre}$ and on OT-I TCR transgenic mice. Control R26R-EYFP x $hCD2^{Cre}$ x OT-I (WT OT-I) mice were also established. Both types of mice were bred on a CD45.1 and CD45.2 background, respectively, to distinguish OT-I T cells originating from the two different genotypes. Using in vitro stimulation with strong and weak agonistic peptides, the SLP-76^{HA} mutation was found without effect on the responsiveness of OT-I T cells to a strong agonist peptide but increased their responsiveness to the G4 weak agonist. Carefully controlled in vivo experiments relying on adoptive transfer and relying on the Q4H7 'intermediate' agonist showed that SLP-76^{HA} mutation increased OT-I T cell proliferation and coincidently rendered the proliferating T cells more apoptotic. Using similar adoptive transfer model and the LCMV-specific P14 TCR, in lieu of OT-I, the authors noted that the SLP-76^{HA} mutation only affected the generation of T_{CM} cells in the early memory phase of the

response to the LCMV Armstrong strain. Finally, the authors analyzed the effect of the SLP-76^{HA} mutation on Tfh development using their adoptive transfer model and the OT-II TCR and found that the SLP-76^{HA} mutation promoted death of pre-Tfh cells during cognate interaction with B cells (see specific comment 4). This very thorough study combines a large body of sophisticated approaches to determine in a convincing manner the effect of SLP-76^{HA} mutation on thymocyte development and peripheral T cell responses. As discussed by the authors, it is very puzzling that the SLP-76^{HA} mutation has such a modest effect when compared with that resulting from the LAT^{G135D} mutation.

We sincerely appreciate the favorable comments and insightful questions from this reviewer. To address the reviewer's concerns, we conducted most of the requested experiments as follows and provided a point-by-point response to all comments, highlighted in blue.

- The efficacy of the Cre-mediated conditional SLP-76^{HA} mutation.
- The effect of the SLP-76 mutation on thymocyte development in OT-I TCR transgenic mice.
- The impact on $CD4^{+}$ T cell differentiation into T follicular regulatory cells.
- The effect on invariant NKT cell development in the thymus.

Specific comments.

1/ Several datasets are expressed as % of YFP^{+} cells (see Figure 2B). In that context, it might be useful to mention the efficiency of deletion in the various conditions reached using $CD4^{Cre}$? In the case the deletion is not substantial, the undeleted WT T cells may have outcompete the apoptotic SLP-76^{HA} expressing T cells. The same applies to the very sophisticated crosses involving the $hCD2^{Cre}$.

We sorted for $TCR\beta^{high}$ $CD5^{int}$ CD4/CD8 double-positive stage 3 (DP3), CD4 single-positive (SP), and CD8 SP thymocyte populations from SLP-$76^{4Cre\text{-}mE8}$ mice to assess the degree of the SLP-76^{HA} mutation mediated by CD4-Cre recombinase. Genomic PCR analysis revealed that all three populations expressing YFP fate-mapping reporter carried the "deleted" $Lcp2^{mE8}$ allele but not the "undeleted" $Lcp2^{L8:21L\text{-}mE8}$ allele (Extended Data Fig. 2a,d). We also performed the same analysis of peripheral $CD4^{+}$ and $CD8^{+}$ T cells from SLP-$76^{2Cre\text{-}mE8}$ mice and confirmed that both cell lineages expressing YFP carried the deleted allele only (Extended Data Fig. 4a). These results indicate that the expression of YFP ensures the completion of the SLP-76^{HA} mutation in both SLP-$76^{4Cre\text{-}mE8}$ and SLP-$76^{2Cre\text{-}mE8}$ mice.

In the revised manuscript, we described these observations in the Results section, lines 197 – 199, 205 – 207, and 275 – 278.

2/ In the case OT-I mice are crossed on $CD4^{Cre}$, does the deletion of the LoxP-flanked SLP-76 cDNA Ex. 8-21/poly A cassette affects the early steps of OT-I T cell development in the thymus?

SLP-$76^{4Cre\text{-}mE8}$ mice carrying a transgene of OT-I TCR, which has a relatively high affinity for self-ligands, had a profound decrease in both $TCR\beta^{high}$ $CD5^{int}$ DP3 and $TCR\beta^{high}$ $CD24^{low/-}$ mature CD8 SP thymocytes without any change in total thymocyte number (Fig. 2m,n and Extended Data Fig. 2h). The degree of such decrease was more pronounced than that in SLP-$76^{4Cre\text{-}mE8}$ mice with a natural, endogenous TCR repertoire (Fig. 2a,b,k,l).

We described these results in the Results section (lines 217 – 221) and in Discussion section (lines 518 – 520) in the revised manuscript.

3/ Can the authors provide references allowing them to qualify the 'PD-1^{int} CXCR5^{int}' cells as pre-Tfh cells?

To strengthen the interpretation of our findings on Tfh differentiation, we added a citation of a review article by Crotty (*Annu. Rev. Immunol.* 2011) to the Result section, line 391. This reference provides a comprehensive overview of the stepwise process of Tfh differentiation including the identification of PD-1^{int} CXCR5^{int} pre-Tfh cells at the T/B border. These cells are described as undergoing further selection to become either germinal center Tfh (GC-Tfh) cells or non-Tfh effector cells.

4/ Have the authors determined the effect of the SLP-76^{HA} mutation on T follicular regulatory cells?

WT CD45.1 and SLP-76$^{2Cre\text{-}mE8}$ CD45.2 OT-II cells were co-transferred into (CD45.1/CD45.2) F1 mice, and the recipients were immunized with OVA/CFA. Ten days after immunization, donor OT-II cells in skin draining LNs were analyzed for Foxp3^{+} CXCR5^{+} T follicular regulatory (Tfr) cells by flow cytometry. Our analysis revealed that neither WT nor SLP-76$^{2Cre\text{-}mE8}$ OT-II donor cells (that were all YFP^{+} CD44^{high}) underwent Tfr differentiation, whereas CD44^{high} CD4^{+} T cells derived from the recipient mice had a small but distinct population of Tfr cells (see Confidential Figure for Reviewer #1).

We could not determine whether the SLP-76^{HA} mutation might influence the differentiation into Tfr cells. This limitation may be due to the OVA/CFA immunization system that stimulates immune response but may not be optimal for inducing Tfr differentiation. However, we could rule out the possibility that the impaired GC reaction observed in the SLP-76$^{2Cre\text{-}mE8}$ OT-II donor group (shown in Fig. 7h–j and Extended Data Fig. 6e–i) was a consequence of enhanced generation of Tfr cells caused by the SLP-76^{HA} mutation.

5/ Is it possible that SLP-76^{HA} and LAT^{G135D} mutations differently affect TCR repertoire?

We do not have pieces of evidence to directly address this question. However, given that SLP-76$^{4Cre\text{-}mE8}$ mice exhibited much more modest changes in the development of thymocytes compared to LAT^{G135D} mice (Lo, et al., *Nat. Immunol.*, 2023), we assume that the SLP-76^{HA} mutation would affect the breadth of TCR repertoire to a lesser extent than the LAT^{G135D} mutation.

6/ Has the SLP-76^{HA} mutation an effect on CD4^{+} iNKT cells?

We analyzed thymocytes from SLP-76$^{4Cre\text{-}mE8}$ thymocytes for invariant NKT (iNKT) cells. To identify these cells, we used the PBS-57/CD1d tetramer that specifically binds to a TCR on iNKT cells. Our analysis revealed that the frequency and number of PBS-57/CD1d tetramer-positive cells (that included both DP and CD4 SP) were significantly increased within YFP^{+} DP thymocytes of SLP-76$^{4Cre\text{-}mE8}$ mice. This increase was more than 2-fold compared to WT mice (Fig. 3a,b). We described these results in the Results section, lines 225 – 233.

7/ SLP-76 is expressed several cell types (https://rstats.immgen.org/Skyline/skyline.html) in addition to T cells and important for their functions. Using appropriate Cre mice, it will be great to document in further studies the effect of the SLP76^{HA} mutation on platelets?

This is an important question that needs to be addressed, but not in the current study focusing on the effect of the SLP-76^{HA} mutation in T cells. However, we added to the Results section, line 169 – 170, a following sentence "SLP-76 is expressed in the T cell lineage as well as in other cell types including myeloid cells, granulocytes, mast cells, NK cells, and platelets", which provided us with a rationale for generating a mouse line with a conditional SLP-76^{HA} mutation but not a germ-line knock-in mutation.

Reviewer #2 (Remarks to the Author):

This paper follows up on earlier work by this group identifying a weak interaction between SLP-76 and PLCγ1 that bridges the pLAT–Gads–SLP-76 and pLAT–PLCγ1 complexes forming the pLAT signalosome downstream of TCR stimulation. The biophysical basis of these interactions is well established, and the first part of this manuscript strengthens those findings by showing that the interaction site between SLP-76 and PLCγ1 is highly conserved and that increasing the affinity of this binding (SLP-76^{HA}) enhances the interaction and leads to stronger PLCγ1 enzymatic activity.

The authors further generated transgenic mice in which SLP-76^{HA} is conditionally expressed under Cre control. By crossing these mice with different Cre strains and employing various immune models, they provide a broad view of the phenotypes of T cells expressing SLP-76^{HA} across different developmental stages and functional contexts. They report that enhancing SLP-76's affinity for PLCγ1 promotes apoptosis of post-selection thymocytes, skews peripheral CD8^{+} T cells toward a memory phenotype under steady-state conditions, increases responsiveness of antigen-specific T cells to weak peptide–MHC complexes, elevates short-lived effector cells (SLECs) during acute LCMV infection while reducing stem-like memory cells, and decreases T_{FH} formation following OVA–CFA immunization.

Overall, the manuscript is clearly written, and the experiments are competently performed. However, several issues merit attention before the paper can be considered for publication.

We sincerely appreciate the favorable comments and insightful questions from this reviewer, which have greatly enhanced the quality of our manuscript. To address these constructive suggestions, we performed most of the requested experiments as listed below. Our responses to specific comments are highlighted in blue.

- Comparison of SLP-76 protein expression between WT and SLP-76^{HA} mutant mice.
- Discrepancy between thymic development and peripheral T cell numbers in SLP-76^{HA} mutant mice.
- The impact on germinal center reaction.
- The effect on TCR-induced phosphorylation of ERK MAP kinase
- The effect on the development of invariant NKT cell sand intraepithelial lymphocyte precursors in the thymus.

Major Comments

1. The conditional expression strategy of SLP-76^{HA} is clever, but the authors should show that the "Lcp2$^{L8:21L–mE8}$" designation does not alter basal SLP-76 expression in thymocyte or

peripheral T cell subsets. Protein-level quantification (e.g., immunoblot or flow cytometry) of SLP-76 in these subsets from $Lcp2^{mE8}$ mice following Cre recombination is recommended.

We sorted for $TCR\beta^{high}$ $CD5^{int}$ CD4/CD8 double-positive stage 3 (DP3), CD4 single-positive (SP), and CD8 SP thymocyte populations from SLP-$76^{4Cre-mE8}$ mice to assess the degree of the SLP-76^{HA} mutation mediated by $CD4^{Cre}$. Genomic PCR analysis revealed that all three populations expressing YFP fate-mapping reporter carried the "deleted" *$Lcp2^{mE8}$* allele but not the "undeleted" *$Lcp2^{L8:21L-mE8}$* allele (Extended Data Fig. 2a,d). We also performed the same analysis of peripheral $CD4^+$ and $CD8^+$ T cells from SLP-$76^{2Cre-mE8}$ mice and confirmed that both cell lineages expressing YFP carried the deleted allele only (Extended Data Fig. 4a). These results indicate that the expression of YFP ensures the completion of the SLP-76^{HA} mutation in both SLP-$76^{4Cre-mE8}$ and SLP-$76^{2Cre-mE8}$ mice.

Moreover, we analyzed for the level of SLP-76 protein expression in DP3, CD4 SP, and CD8 SP thymocytes by flow cytometry. The anti-SLP-76 mAb used for this study binds to the SH2 domain of SLP-76, therefore it is unlikely that the SLP-76^{HA} mutation (in the proline-rich region) would influence the mAb binding to its epitope. We found that when gating on YFP^+ cells, there was almost no difference between WT and SLP-$76^{4Cre-mE8}$ thymocyte subpopulations ($TCR\beta^{high}$ $CD5^{int}$ DP3, CD4 SP, and CD8 SP) in the level of SLP-76 (Extended Data Fig. 2e,f). We also confirmed that SLP-$76^{2Cre-mE8}$ $CD4^+$ and $CD8^+$ T cells expressed a comparable level of SLP-76 to those cells in WT mice (Extended Data Fig. 4b,c).

Of note, in the absence of Cre recombinase, the *$Lcp2^{L8:21L-mE8}$* allele produced approximately half as much SLP-76 as did the *$Lcp2^{WT}$* allele (Extended Data Fig. 4a–c). However, this decreased expression of SLP-76 did not affect the peripheral T cell pool in the *$Lcp2^{L8:21L-mE8}$* mice under specific pathogen-free conditions (Extended Data Fig. 4d). Whether the reduced expression of SLP-76 is regulated at a transcriptional or post-transcriptional level requires further investigation.

In the revised manuscript, we described these observations in the Results section, lines 197 – 199, 205 – 207, and 275 – 281.

2. The authors claim enhanced negative selection based on increased $CC3^+$ post-selection thymocytes. If so, why does this not lead to a reduction in total mature $CD4^+$ or $CD8^+$ SP thymocytes or peripheral T cell numbers? This discrepancy should be addressed experimentally or discussed.

SLP-$76^{4Cre-mE8}$ mice carrying an OT-I TCR transgene, which has a relatively high affinity for self-ligands, had a profound decrease in both $TCR\beta^{high}$ $CD5^{int}$ DP3 and $TCR\beta^{high}$ $CD24^{low/-}$ mature CD8 SP thymocytes. Notably, this decrease occurred without any change in the overall number of thymocytes (Fig. 2m,n and Extended Data Fig. 2h).

Moreover, SLP-$76^{4Cre-mE8}$ OT-I mice had a decrease in the total number of lymph node cells and YFP^+ $TCRV\alpha2^+$ $CD8^+$ T cells. However, there was a 2-fold increase in $CD44^{high}$ Ly-$6C^{high}$ memory-phenotype T cells due to a large increase in the frequency of this population (Fig. 4g and Extended Data Fig. 3i–k).

These results indicate that shifting the TCR repertoire toward high affinity for self-ligands significantly affects thymocyte selection and homeostasis of peripheral T cells in SLP-$76^{4Cre-mE8}$ mice.

We described these findings in the Results section (lines 217 – 221, and 261 – 264) and in the Discussion section (lines 517 – 519) in the revised manuscript.

3. The data show more SLECs in SLP-76$^{2Cre\text{–}mE8}$ P14 cells at day 7 post LCMV-Armstrong infection. Would this translate into faster viral clearance or altered secondary responses upon reinfection? Experimental data supporting these implications would strengthen the claims.

We recognize the importance of this question and intended to perform the experiment to evaluate how the SLP-76^{HA} mutation would affect the recall response to LCMV infection. However, limited cage availability for our animal study proposal made it difficult to carry out this experiment along with others requested by the reviewers. The additional experiments required the generation of new mouse lines by complex breeding within a restricted timeframe for manuscript revision. We will include this question in our future research plan.

4. The authors should clarify the classification of CD127^{-} KLRG1^{+}, CD127^{+} KLRG1^{-}, and TCF1^{+} KLRG1^{-} populations as SLECs, MPECs, and stem-like memory cells, respectively, rather than using T_{EM}/T_{CM} nomenclature, which applies to CD44/CD62L-based definitions (Kaech et al., Cell 2003; Kaech & Cui, Nat Rev Immunol 2012).

The SLEC (short-lived effector cells)/MPEC (memory precursor effector cells) nomenclature is used to classify heterogenous CD8^{+} T cell populations specifically during the expansion phase of an acute viral infection, in which viral antigens are actively present and driving T cell differentiation. We used this nomenclature for our analysis of WT and SLP-76$^{2Cre\text{-}mE8}$ P14 donor cells at 7 days post-infection (dpi) with the LCMV Armstrong ($LCMV_{Arm}$) strain, using CD127 and KLRG1 expression as markers (Fig. 6a,b). The vast majority of SLEC die off during the contraction phase, but some MPEC survive and subsequently differentiate into either effector memory T cells (T_{EM}) or central memory T cells (T_{CM}). Thus, the SLEC/MPEC nomenclature may not be appropriate to classify two distinct LCMV-specific CD8^{+} T cell populations in the memory phase. As the reviewer pointed out, T_{EM} and T_{CM} subsets were originally defined based on the expression of CD44 and CD62L accompanied by their homing capacity to the secondary lymphoid organs. But we instead defined CD127^{-} KLRG1^{+} cells as T_{EM} cells and CD127^{+} KLRG1^{-} cells as T_{CM} cells in our analysis of the P14 donor cells at 28 dpi with $LCMV_{Arm}$.

Based on the suggestion regarding the TCF1^{+} KLRG1^{-} population, we revised our classification from T_{CM} cells to stem-like memory T cells in the Results section (line 362).

We re-analyzed the same dataset used for Figure 6c–h to assess the expression of CD44 and CD62L on WT CD45.1 and SLP-76$^{2Cre\text{-}mE8}$ CD45.2 P14 donor cells at 28 dpi with $LCMV_{Arm}$ (Confidential Figure for Reviewer #2, f–m). Both donor cells (gated on live I-A^{b-} CD3^{+} CD4^{-} YFP^{+} CD8a^{+} cells) had three distinct populations: CD44^{int} CD62L^{-}, CD44^{hi} CD62L^{-}, and CD44^{hi} CD62L^{+}. The SLP-76^{HA} mutation altered the frequency of CD44^{int} CD62L^{-} and CD44^{hi} CD62L^{-} subpopulations. However, this mutation did not affect the absolute number of CD44^{int} CD62L^{-} and CD44^{hi} CD62L^{+} cells, except for a slight reduction in CD44^{hi} CD62L^{-} cells (Confidential Figure for Reviewer #2, f–h).

When these 3 subpopulations were further analyzed for CD127 and KLRG1 expression, a large proportion of CD44^{int} CD62L^{-} cells differentiated into CD127^{-} KLRG1^{+} T_{EM} cells, and the SLP-76^{HA} mutation significantly increased the frequency and absolute number of T_{EM} cells (Confidential Figure for Reviewer #2, f,i,j). In contrast, both CD44^{hi} CD62L^{-} and

CD44^{hi} CD62L^{+} cells predominantly skewed toward CD127^{+} KLRG1^{-} T_{CM} cells, and the SLP-76^{HA} mutation significantly decreased the frequency and absolute number of T_{CM} cells (Confidential Figure for Reviewer #2, f,i,j).

Additionally, we analyzed the CD44^{int} CD62L^{-}, CD44^{hi} CD62L^{-}, and CD44^{hi} CD62L^{+} subpopulations for TCF1 and KLRG1 expression. Consistent with the T_{EM}/T_{CM} analysis shown in Confidential Figure for Reviewer #2, f–m, the CD44^{int} CD62L^{-} subpopulation mostly differentiated into TCF1^{-} KLRG1^{+} cells, and the SLP-76^{HA} mutation promoted this differentiation (Confidential Figure for Reviewer #2, k–m). However, both the CD44^{hi} CD62L^{-} and CD44^{hi} CD62L^{+} subpopulations preferentially developed into TCF1^{+} KLRG1^{-} stem-like memory T cells. The SLP-76^{HA} mutation reduced the polarization toward stem-like memory cells (Confidential Figure for Reviewer #2, k–m).

In summary, the SLP-76^{HA} mutation influences the differentiation state of LCMV-specific CD8^{+} T cells in the memory phase with a minimal impact on CD44 and CD62L expression.

5. The reduced T_{FH} population in SLP-76$^{2Cre–mE8}$ OT-II mice raises the question of whether this defect affects germinal center B cell formation or antigen-specific antibody titers. Experimental validation would be helpful.

We transferred WT or SLP-76$^{2Cre-mE8}$ OT-II CD4^{+} T cells (CD45.2 for both genotype) into SAP KO CD45.1 mice, which have severely impaired humoral immune response that can be restored by reconstitution with SAP-intact CD4^{+} T cells. One day later, recipient mice were immunized with NP-OVA/CFA. Consistent with co-transfer experiments (Fig. 7a–e), SLP-76$^{2Cre-mE8}$ OT-II cells exhibited a decreased frequency of Bcl-6^{+} CXCR5^{+} mature Tfh cells in SAP KO recipients 10 days after NP-OVA-immunization (Extended Data Fig. 6e,f). Moreover, SAP KO mice receiving SLP-76$^{2Cre-mE8}$ OT-II cells showed impaired generation of total GC B cells (CD19^{+} CD38^{-} IgD^{-} Blimp-1^{-} CD138^{-} CD95^{+} GL7^{+}). The number of NP-specific (Bcl-6^{+} NP^{+}) GC B cells was decreased by 3.8-fold, but the difference between WT and SLP-76$^{2Cre-mE8}$ OT-II donor groups was not statistically significant due to a high variation in NP^{+} population frequency within GC B cells. Notably, NP-specific GC B cells in the SLP-76$^{2Cre-mE8}$ OT-II donor group showed markedly impaired immunoglobulin class switching, as indicated by a decreased IgG1/IgM ratio (Fig. 7h–j and Extended Data Figure 6e–i). These results indicate that the defective Tfh response caused by the SLP-76^{HA} mutation impairs the induction of GC reaction.

We described these observations in the Results section (lines 403 – 426) and in the Discussion section (lines 569 – 580) in the revised manuscript.

6. The central mechanistic claim is that SLP-76^{HA} enhances TCR signaling, yet no direct signaling data are shown. The authors should measure pERK1/2, Nur77 expression, Ca^{2+} flux, or NFAT/NF-κB activation in mutant versus WT cells to substantiate this assertion.

We co-cultured WT CD45.1 and SLP-76$^{2Cre-mE8}$ CD45.2 OT-I CD8^{+} T cells with T cell-depleted spleen (TdS) cells from (CD45.1/CD45.2) F1 mice. These TdS cells were pre-loaded with either 10^{-11} M N4 or 10^{-9} M Q4H7 peptides, which induced the EC_{50} of CD69 expression (Fig. 5a,b). The co-cultures were analyzed at multiple time points (2, 5, 10, 30, 60, and 90 minutes) to compare the phosphorylation levels of ERK1/2 MAP kinase (pERK) between the two OT-I cell types using flow cytometry and ImageStream. Q4H7 stimulation led to a substantial decrease and a kinetic delay in the formation of OT-I/TdS conjugates

(Fig. 5c,d). SLP-76$^{2Cre\text{-}mE8}$ OT-I cells formed conjugates with TdS cells at the same degree and kinetics as WT OT-I cells (Fig. 5c,d). When gating on OT-I cells interacting with TdS cells ($CD45.2^+$ I-A^{b+} for WT and $CD45.1^+$ I-A^{b+} for SLP-76$^{2Cre\text{-}mE8}$ within the $CD3^+$ $CD8^+$ YFP^+ population), there was no difference in the level of pERK between WT and SLP-76$^{2Cre\text{-}mE8}$ OT-I cells stimulated with N4 at any time points (Fig. 5c,e). In contrast, Q4H7 stimulation increased the frequency of $pERK^+$ SLP-76$^{2Cre\text{-}mE8}$ OT-I cells by 2-fold compared to WT OT-I cells but induced pERK less robustly than N4 stimulation with a delayed kinetics (Fig. 5c,e). Furthermore, ImageStream analysis revealed that pERK induction occurred in OT-I cells interacting with N4-loaded TdS cells, not in TdS cells themselves, and that pERK was barely detectable in either OT-I or TdS singlet (Extended Data Fig. 4j,k). These findings indicate that the SLP-76^{HA} mutation enhances TCR signaling strength in response to weak agonists, as evidenced by the increased frequency of $pERK^+$ SLP-76$^{2Cre\text{-}mE8}$ OT-I cells under Q4H7 stimulation.

We described these observations in the Results section (lines 309 – 325) and in the Discussion section (line 536) in the revised manuscript.

7. Since SLP-76 is essential for pre-TCR signaling, it would be highly informative to test whether the SLP-76NA mutant (which cannot bind PLCγ1) supports thymocyte development beyond the DN3 stage. Comparison between SLP-76 knockout and SLP-76NA mice would add valuable mechanistic insight.

As suggested by the reviewer, we also considered generating mice with a conditional SLP-76NA mutation using T cell-specific Cre transgenic systems. Indeed, SLP-76Δ185-194 mice lacking the amino acids 185-194 of SLP-76, identified as the minimal binding site for the PLC-γ1 SH3 domain, exhibit compromised thymocyte development at several stages due to severely impaired TCR signaling (Kumar et al., *Proc. Natl. Acad. Sci. U.S.A.*, 2005). Based on this previous report, we assumed that the conditional SLP-76NA mutant mice would largely phenocopy the SLP-76Δ185-194 mice. Therefore, we decided to pursue a different strategy and generate the conditional SLP-76^{HA} mutant mouse lines to investigate thymic development and peripheral T cell function under the conditions where TCR signaling would expect to be enhanced.

Rather than generating mice with SLP-76NA mutation, we bred *Lcp2*$^{L8:21L\text{-}mE8}$ mice onto proximal Lck^{Cre} transgenic mice (B6.Cg-Tg(Lck-cre)548Jxm/J, Strain #: 003802, The Jackson Laboratory), to examine the effect of the SLP-76^{HA} mutation on β-selection. We first noted that the proximal Lck promoter drove promiscuous expression of Cre recombinase, as judged by the expression of YFP fate-mapping reporter, and about 30% of WT and *Lcp2*$^{pLCre\text{-}mE8}$ mice had YFP expression in a T cell-specific manner (Confidential Fig. a,b for Reviewer #2). Using those mice with the T cell lineage-specific Cre activity, we found that a DN4/DN3 ratio within YFP^+ DN thymocytes was elevated in *Lcp2*$^{pLCre\text{-}mE8}$ mice (Confidential Fig. c,d for Reviewer #2). Moreover, the frequency and absolute number of a $TCR\beta^+$ $CD5^{int/high}$ population within YFP^+ DN4 thymocytes were also increased in *Lcp2*$^{pLCre\text{-}mE8}$ mice (Confidential Fig. c,e for Reviewer #2). These results indicate that the SLP-76^{HA} mutation promotes β-selection during the transition from DN3 to DN4 stages. This highlights the impact of the enhanced TCR signaling caused by the SLP-76^{HA} mutation on thymocyte maturation and provides mechanistic insight into the regulation of early T cell development.

8. The authors note that the SLP-76^{HA} mutation preferentially enhances responses to weak agonists. It would be useful to analyze other “agonist-selected” T cells, such as NKT or CD8αα^{+} intraepithelial lymphocytes, to test this model more broadly.

We analyzed thymocytes from SLP-76$^{4Cre\text{-}mE8}$ thymocytes for invariant NKT (iNKT) cells. To identify these cells, we used the PBS-57/CD1d tetramer that specifically binds to a TCR on iNKT cells. Our analysis revealed that the frequency and number of PBS-57/CD1d tetramer-positive cells (that included both DP and CD4 SP) were significantly increased within YFP^{+} DP thymocytes of SLP-76$^{4Cre\text{-}mE8}$ mice. This increase was more than 2-fold compared to WT mice (Fig. 3a,b).

We also examined the development of intraepithelial lymphocyte precursors (IELp), which migrate from thymus to the gut and differentiate into CD8αα^{+} IEL. There was no difference between WT and SLP-76$^{4Cre\text{-}mE8}$ mice in the absolute number of YFP^{+} IELp (defined as TCRγδ^{-} CD25^{-} PBS-57/CD1d tetramer-negative, CD4/CD8 DN thymocytes expressing TCRβ, CD5, CD122, and H-2K^{b}) (Fig. 3c,d). We further analyzed for Type A (T-bet^{-} NK1.1^{-} PD-1^{+}) and Type B (T-bet^{+} NK1.1^{+} PD-1^{-}) IELp subsets, which differ in thymic localization and TCR specificity (Ruscher et al., *Nat. Immunol.*, 2017). The frequency of NK1.1^{-} PD-1^{+} Type A IELp was slightly but significantly decreased in SLP-76$^{4Cre\text{-}mE8}$ mice, although the absolute number of these cells was comparable to WT mice (Fig. 3c,e). In contrast, both the frequency and number of NK1.1^{+} PD-1^{-} Type B IELp were increased by approximately 3-fold in SLP-76$^{4Cre\text{-}mE8}$ mice (Fig. 3c,e).

In summary, the SLP-76^{HA} mutation significantly promotes the development of agonist-selected iNKT cells and Type B IELp. Further investigation is needed to determine how this mutation would alter the homeostasis and functional properties of these T cells in the secondary lymphoid organs and non-immune tissues.

We added the description on these results in the Results section (lines 225 – 242) and in the Discussion section (lines 527 – 531).

This study provides an elegant genetic approach to modulate the SLP-76–PLCγ1 interaction and explores its impact across multiple T cell contexts. However, without direct signaling data and deeper mechanistic analysis, the functional consequences of enhanced SLP-76–PLCγ1 binding appear limited, which may constrain the implications of these findings for future work.

Reviewer #3 (Remarks to the Author):

I co-reviewed this manuscript with one of the reviewers who provided the listed reports. This is part of the Nature Communications initiative to facilitate training in peer review and to provide appropriate recognition for Early Career Researchers who co-review manuscripts.

We thank this reviewer for his/her comments that help us improve our manuscript.

Reviewer #1 (Remarks to the Author):

None

We thank this reviewer for his/her comments that helped us improve our manuscript.

Reviewer #2 (Remarks to the Author):

The authors have done an excellent job in responding to the previous critiques.

There is one minor concern regarding the statistical significance reported in Fig. 7i, left panel, for SLP-76$^{2Cre\text{-}mE8}$ vs. SAP KO. The differences in the bar graphs do not appear to be consistent with "ns". At least to my eyes, this seems more likely to be significant at least the two-star level.

We sincerely appreciate the favorable comments from this reviewer. Using the nonparametric Kruskal-Wallis test, we found significant differences between the SLP-76$^{2Cre\text{-}mE8}$ and SAP KO donor groups in the number of total germinal center (GC) B cells (p=0.0301) and NP-specific GC B cells (p=0.0476), shown in Fig. 7h–k. These outcomes differ from the previous analysis due to the use of an inappropriate parametric one-way ANOVA.

Reviewer #3 (Remarks to the Author):

I co-reviewed this manuscript with one of the reviewers who provided the listed reports. This is part of the Nature Communications initiative to facilitate training in peer review and to provide appropriate recognition for Early Career Researchers who co-review manuscripts.

We thank this reviewer for his/her comments that helped us improve our manuscript.
