# Supplementary material for: Weak SLP-76/PLC-γ1 interaction fine-tunes T cell receptor signal strength to optimize T cell responsiveness
Source: Nat Commun. 2026 Jul 29;17:9189. doi: 10.1038/s41467-026-75599-0 (PMC13522589; doi:10.1038/s41467-026-75599-0)
Supplement: Supplementary file 3 [file 41467_2026_75599_MOESM3_ESM.pdf]

nature portfolio

Corresponding author(s): Hidehiro Yamane & Lawrence E. Samelson

Last updated by author(s): Jun 9, 2026

Reporting Summary

Nature Portfolio wishes to improve the reproducibility of the work that we publish. This form provides structure for consistency and transparency in reporting. For further information on Nature Portfolio policies, see our Editorial Policies and the Editorial Policy Checklist.

Statistics

For all statistical analyses, confirm that the following items are present in the figure legend, table legend, main text, or Methods section.

n/a	Confirmed	
☐	☒	The exact sample size (*n*) for each experimental group/condition, given as a discrete number and unit of measurement
☐	☒	A statement on whether measurements were taken from distinct samples or whether the same sample was measured repeatedly
☐	☒	The statistical test(s) used AND whether they are one- or two-sided<br>*Only common tests should be described solely by name; describe more complex techniques in the Methods section.*
☒	☐	A description of all covariates tested
☐	☒	A description of any assumptions or corrections, such as tests of normality and adjustment for multiple comparisons
☐	☒	A full description of the statistical parameters including central tendency (e.g. means) or other basic estimates (e.g. regression coefficient) AND variation (e.g. standard deviation) or associated estimates of uncertainty (e.g. confidence intervals)
☐	☒	For null hypothesis testing, the test statistic (e.g. *F*, *t*, *r*) with confidence intervals, effect sizes, degrees of freedom and *P* value noted<br>*Give P values as exact values whenever suitable.*
☒	☐	For Bayesian analysis, information on the choice of priors and Markov chain Monte Carlo settings
☒	☐	For hierarchical and complex designs, identification of the appropriate level for tests and full reporting of outcomes
☒	☐	Estimates of effect sizes (e.g. Cohen's *d*, Pearson's *r*), indicating how they were calculated

Our web collection on statistics for biologists contains articles on many of the points above.

Software and code

Policy information about availability of computer code

Data collection	Flow cytometry: BD FACSDiva™ Software (BD Biosciences)<br>ImageStream: INSPIRE™ Software (Cytec Biosciences)
Data analysis	In vitro IP3 assay: OpenChrom (Lablicate GmbH) and ImageJ<br>Isothermal titration calorimetry: PEAQ-ITC analysis software (Malvern Panalytical)<br>Flow cytometry: FlowJo 10 software (BD Biosciences)<br>ImageStream: IDEAS 6.03 (Cytec Biosciences)<br>Graphs and statistics : GraphPad Prism v10

For manuscripts utilizing custom algorithms or software that are central to the research but not yet described in published literature, software must be made available to editors and reviewers. We strongly encourage code deposition in a community repository (e.g. GitHub). See the Nature Portfolio guidelines for submitting code & software for further information.

Data

Policy information about availability of data

All manuscripts must include a data availability statement. This statement should provide the following information, where applicable:

- Accession codes, unique identifiers, or web links for publicly available datasets
- A description of any restrictions on data availability
- For clinical datasets or third party data, please ensure that the statement adheres to our policy

All data used and analyzed for the associated manuscript are included in this published article and available from the corresponding authors.

Research involving human participants, their data, or biological material

Policy information about studies with human participants or human data. See also policy information about sex, gender (identity/presentation), and sexual orientation and race, ethnicity and racism.

Reporting on sex and gender	N/A
Reporting on race, ethnicity, or other socially relevant groupings	N/A
Population characteristics	N/A
Recruitment	N/A
Ethics oversight	N/A

Note that full information on the approval of the study protocol must also be provided in the manuscript.

Field-specific reporting

Please select the one below that is the best fit for your research. If you are not sure, read the appropriate sections before making your selection.

[x] Life sciences [ ] Behavioural & social sciences [ ] Ecological, evolutionary & environmental sciences

For a reference copy of the document with all sections, see nature.com/documents/nr-reporting-summary-flat.pdf

Life sciences study design

All studies must disclose on these points even when the disclosure is negative.

Sample size	The sample size was determined based on a power analysis using data from previous studies and internal pilot experiments. The number of samples in each group is specified in the figure legends.
Data exclusions	Data were excluded from analyses only when animals were found to have health conditions unrelated to the experiments, such as significant weight loss, lethargy, or signs of infection.
Replication	Experiments were carried out at least two times as independent biological replicates to verify data reproducibility. Two-tailed Student's t-test was used for pairwise comparisons, while one-way ANOVA and two-way ANOVA were applied to multi-group analyses as appropriate. Statistical significance was assessed with p-values less than 0.05 considered significant. Reproducibility and significance of the results are described in the figure legends.
Randomization	Randomization was deemed unnecessary because allocation was based strictly on predetermined genotype, age, and sex criteria, ensuring comparability between groups. Mice were selected so that each experimental group contained animals of the same age and sex, minimizing potential confounding variables. No randomization was performed, as allocation of mice was determined by these matching criteria.
Blinding	A formal blind group assignment was not applied since all data were collected independently of biased intervention. No pre-selection screening was performed other than to ensure that all experimental mice were healthy. Mice were assigned to experimental groups using a random number generator to ensure unbiased allocation.

Reporting for specific materials, systems and methods

We require information from authors about some types of materials, experimental systems and methods used in many studies. Here, indicate whether each material, system or method listed is relevant to your study. If you are not sure if a list item applies to your research, read the appropriate section before selecting a response.

Materials & experimental systems

n/a	Involved in the study	
☐	☒	Antibodies
☐	☒	Eukaryotic cell lines
☒	☐	Palaeontology and archaeology
☐	☒	Animals and other organisms
☒	☐	Clinical data
☒	☐	Dual use research of concern
☒	☐	Plants

Methods

n/a	Involved in the study	
☒	☐	ChIP-seq
☐	☒	Flow cytometry
☒	☐	MRI-based neuroimaging

Antibodies

Antibodies used

Antibodies used in this study (Supplier name, Catalog number, Clone name, RRID, and dilution for staining)
Surface molecules:
BUV395 anti-CD4 (BD Biosciences, 568375, RM4-5, AB_3095981, 1:200), BUV395 anti-CD19 (BD Biosciences, 565965, 1D3, AB_2722495, 1:100), BUV737 anti-CD45.2 (BD Biosciences, 612779, 104, AB_2870107, 1:200), BUV737 anti-TCRβ (BD Biosciences, 612821, H57-597, AB_2870145, 1:200), BUV805 anti-CD38 (BD Biosciences, 741955, 90/CD38, AB_2871263, 1:200), BUV805 anti-CD44 (BD Biosciences, 741921, IM7, AB_2871234, 1:200), BV421 anti-Vα2 TCR (BD Biosciences, 562944, B20.1, AB_2737910, 1:100), BV421 anti-NK1.1 (BD Biosciences, 562921, PK136, AB_2728688, 1:200), BV421 anti-CCR7 (BioLegend, 120120, 4B12, AB_2561446, 1:60), BV421 anti-CXCR5 (BioLegend, 145512, L138D7, AB_2562128, 1:100), BV421 anti-PD-1 (BioLegend, 135221, 29F.1A12, AB_2562568, 1:100), BV421 anti-KLRG1 (BioLegend, 138414, 2F1/KLRG1, AB_2565613, 1:100), BV510 anti-CD19 (BD Biosciences, 562956, 1D3, AB_2737915, 1:100), BV510 anti-γδTCR (BD Biosciences, 563218, GL3, AB_2716858, 1:100), BV510 anti-I-A/I-E (BioLegend, 107636, M5/114.15.2, AB_2734168, 1:200), BV510 anti-NK1.1 (BioLegend, 108738, PK136, AB_2562217, 1:100), BV510 anti-H-2Kb (BioLegend, 116523, AF6-88.5, AB_2800584, 1:200), BV510 anti-IgD (BioLegend, 405723, 11-26c.2a, AB_2562742, 1:200), BV605 anti-CD62L (BioLegend, 104438, MEL-14, AB_2563058, 1:200), BV650 anti-γδTCR (BD Biosciences, 563993, GL3, AB_2738530, 1:100), BV650 anti-mouse IgG1 (BioLegend, 406629, RMG1-1, AB_2716013, 1:400), BV711 anti-CD24 (BD Biosciences, 563450, M1/69, AB_2738213, 1:400), BV711 anti-CD95 (BD Biosciences, 740716, Jo2, AB_2740397, 1:100), BV711 anti-CD103 (BD Biosciences, 564320, M290, AB_2738743, 1:200), BV786 anti-Vα2 TCR (BD Biosciences, 743833, B20.1, AB_2741792, 1:200), BV785 anti-CD138 (BioLegend, 142534, 281-2, AB_2814047, 1:200), BV785 anti-I-A/I-E (BioLegend, 107645, M5/114.15.2, AB_2565977, 1:1000), BV785 anti-Ly-6C (BioLegend, 128041, HK1.4, AB_2565852, 1:1000), BV785 anti-PD-1 (BioLegend, 135225, 29F.1A12, AB_2563680, 1:200), PerCP-Cy5.5 anti-CD5 (BioLegend, 100624, 53-7.3, AB_2563433, 1:200), PerCP-Cy5.5 anti-CD25 (BioLegend, 102030, PC61, AB_893288, 1:200), RB705 anti-GL7 (BD Biosciences, 570690, GL7, AB_3668959, 1:2000), PE anti-PD-1 (BioLegend, 135206, 29F.1A12, AB_1877231, 1:200), PE-Dazzle 594 anti-CD122 (BioLegend, 123218, TM-β1, AB_2572180, 1:100), PE-Dazzle 594 anti-CCR7 (BioLegend, 120122, 4B12, AB_2564317, 1:20), PE-Dazzle 594 anti-CXCR5 (BioLegend, 145522, L138D7, AB_2563644, 1:100), PE-Cy5 anti-CD127 (BioLegend, 135016, A7R34, AB_1937261, 1:100), PE-Cy5 anti-mouse IgM (BioLegend, 406544, RMM-1, AB_2876730, 1:200), PE-Cy7 anti-CD45.1 (BD Biosciences, 560578, A20, AB_1727488, 1:200), PE-Cy7 anti-CD5 (BioLegend, 100622, 53-7.3, AB_2562773, 1:500), PE-Cy7 anti-CD25 (BioLegend, 102016, PC61, AB_312865, 1:500), APC anti-CD69 (BioLegend, 104514, H1.2F3, AB_49843, 1:200), R718 anti-CD3 (BD Biosciences, 567296, 17A2, AB_2916539, 1:500), APC-eFluor 780 anti-CD8a (eBiosciences, 47-0081-82, 53-6.7, AB_1272185, 1:200), APC-Cy7 anti-CD19 (BioLegend, 115530, 6D5, AB_830707, 1:200), and purified anti-CD16/CD32 (BioLegend, 101302, 93, AB_312801, 10 μg/mL).

Intracellular molecules (cytoplasmic & nuclear):
RB705 anti-TCF1 (BD Biosciences, 570721, S33-966, AB_3685913, 1:200), PE anti-SLP-76 (BD Biosciences, 560058, H3, AB_1645531, 1:10), PE-CF594 anti-Blimp-1 (BD Biosciences, 564269, 5E7, AB_2738719, 1:200), Alexa Flour 647 anti-Bcl-6 (BD Biosciences, 561525, K112-91, AB_10898007, 1:60), Alexa Flour 647 anti-ERK1/2 pT202/pY204 (BD Biosciences, 561992, 20A, AB_10896138, 1:10), Alexa Flour 488 anti-GFP (BioLegend, 338008, FM264G, AB_2563288, 1:200), APC anti-Helios (BioLegend, 137222, 22F6, AB_10662900, 1:200), PE-eFluor 610 anti-Foxp3 (eBiosciences, 61-5773-82, FJK-16s, AB_2574624, 1:100), and PE anti-Cleaved Caspase-3 (Cell Signaling Technology, 12768, D3E9, AB_2798021, 1:100).

Biotinylated antibodies for isolation of donor OT-I, P14, and OT-II cells as well as T cell-depleted spleen cells (all used at 1:1000):
Anti-γδTCR (BD Biosciences, 553176, GL3, AB_394687), anti-CD3 (BioLegend, 100244,17A2, AB_2563947), anti-CD4 (BioLegend, 100508, RM4-5, AB_312711), anti-CD8a (BioLegend, 100704, 53-6.7, AB_312743), anti-CD11b (BioLegend, 101204, M1/70, AB_312787), anti-CD11c (BioLegend, 117304, N418, AB_313773), anti-CD25 (BioLegend, 102004, PC61, AB_312853), anti-CD49b (BioLegend, 108904, DX5, AB_313411), anti-CD69 (BioLegend, 104504, H1.2F3, AB_313107), anti-CD90.2 (BioLegend, 105304, 30-H12, AB_313175), anti-NK1.1 (BioLegend, 108704, PK136, AB_313391), anti-ICOS (BioLegend, 313504, C398.4A, AB_416328), anti-PD-1 (BioLegend, 135212, 29F.1A12, AB_10640124), anti-I-A/I-E (BioLegend, 107604, M5/114.15.2, AB_313319), anti-TER-119 (BioLegend, 116204, TER-119, AB_313705), anti-CD16/CD32 (eBiosciences, 13-0161-85, 93, AB_466377), and anti-CD19 (eBiosciences, 13-0193-85, 1D3, AB_657656).

Validation

All antibodies used in this study are commercially available and have been validated by the vendors. We selected antibodies that are commonly used in immunology research, ensuring reproducibility and reliability. To optimize staining conditions for flow cytometry and ImageStream analyses, we performed serial dilutions of each antibody to determine the optimal concentration for signal-to-noise ratio. Proper isotype controls were used in some experiments to assess specificity and minimize background staining. Criteria for optimal concentration included maximizing specific signal while minimizing non-specific binding, as determined by comparison to isotype controls and fluorescence intensity measurements.

Eukaryotic cell lines

Policy information about cell lines and Sex and Gender in Research

Cell line source(s)	Sf9 cells were purchased from Thermo Fisher Scientific, Cat. No. 12659017.<br>Tni-FNL cells were derived from BTI-Tn-5B1-4 (High Five) cells by the National Cancer Institute (NCI).
Authentication	Sf9 cells were not authenticated by DNA analysis, but had the stereotypical appearance/morphology of Sf9 cells.<br>Tni-FNL cell line was not authenticated using external profiling (or state the method used, if any).
Mycoplasma contamination	Both cell lines are routinely confirmed as Mycoplasma-free using the MycoALERT kit (Lonza).
Commonly misidentified lines<br>(See ICLAC register)	No commonly misidentified cell lines were used in this study.

Animals and other research organisms

Policy information about studies involving animals; ARRIVE guidelines recommended for reporting animal research, and Sex and Gender in Research

Laboratory animals	All mice used in the present study were on a C57BL/6 background. Lcp2L8:21L-mE8 mice used for this study were newly generated by ourselves, as described in the Methods section of the main text. C57BL/6J (B6/J) mice (Strain #: 000664), B6 CD45.1 mice (B6.SJL-PtprcaPepcb/BoyJ; Strain #: 002014), CD4Cre mice (B6.Cg-Tg(Cd4-cre)1Cwi/BfluJ; Strain #: 022071), pLckCre mice (B6.Cg-Tg(Lck-cre)548Jxm/J, Strain #: 003802), OT-I mice (C57BL/6-Tg(TcraTcrb)1100Mjb/J; Strain #: 003831), OT-II mice (B6.Cg-Tg(TcraTcrb)425Cbn/J; Strain #: 004194), and Rosa26LSL-eYFP mice (B6.129X1-Gt(ROSA)26Sortm1(EYFP)Cos/J; Strain #: 006148) were purchased from The Jackson Laboratory. C57BL/6NCr (B6/Ncr) mice used as a source of fertilized one-cell embryos for microinjection were purchased from Charles River Laboratories. P14 mice (C57BL/6Ai-[Tg]TCR P14 LCMV N10) were purchased from Taconic Biosciences. hCD2Cre mice (C57BL/6-Tg(CD2-cre)1Lov/J) and SAP KO mice (B6.129S6-Sh2d1atm1Pls/J) were a kind gift from Dr. Paul. E. Love (NICHD, NIH) and Dr. Pamela L. Schwartzberg (NIAID, NIH), respectively. Mice aged 8 to 20 weeks were used for experiments and housed in specific pathogen-free facilities at NCI/NIH.
Wild animals	N/A
Reporting on sex	We used both females and males in most experiments. In adoptive T cell transfer experiments, only TCR transgenic females were used as a source of donor T cells, since female-derived donor cells could engraft in both female and male recipients. This strategy allowed us to include recipients of both sexes, thereby increasing our sample size and strengthening the reliability of statistical comparisons. Recipients were matched for age and health status to minimize confounding variables, and sex was considered to ensure balanced experimental groups.
Field-collected samples	N/A
Ethics oversight	All the experiments using laboratory animals were approved by the NCI Animal Care and Use Committee (Animal Study Proposals # 23-074, LCMB-013, and LICI-035.

Note that full information on the approval of the study protocol must also be provided in the manuscript.

Plants

Seed stocks	N/A
Novel plant genotypes	N/A
Authentication	N/A

Flow Cytometry

Plots

Confirm that:

- [x] The axis labels state the marker and fluorochrome used (e.g. CD4-FITC).
- [x] The axis scales are clearly visible. Include numbers along axes only for bottom left plot of group (a 'group' is an analysis of identical markers).
- [x] All plots are contour plots with outliers or pseudocolor plots.
- [x] A numerical value for number of cells or percentage (with statistics) is provided.

Methodology

Sample preparation	Thymi, lymph nodes, and spleens were excised from mice, gently mashed on a 40 µm cell strainer, and suspended in PBS containing 2% fetal bovine serum (FBS). For single cell preparation from spleens, red blood cells were depleted by treating spleen cell suspensions with ACK lysis buffer, followed by washing with 2% PBS/PBS. In some experiments, purified OT-I CD8+ T cells that had been stimulated in vitro with ovalbumin peptide-loaded T cell-depleted spleen cells were harvested and suspended in 2% FBS/PBS. Detailed procedures for sample preparation are described in the Materials & Methods section of the main text.
Instrument	FACSymphony A5 (BD Biosciences) and ImageStream Mk II (Cytec Biosciences)
Software	FlowJo 10 software (BD Biosciences) and IDEAS 6.03 (Cytec Biosciences)
Cell population abundance	We acquired thymocytes, lymph node cells, and spleen cells on FACSymphony A5, typically collecting 10,000 cells from the population of interest in most experiments. These cell numbers were selected to ensure sufficient statistical power and reliable analysis for each experimental condition. When the population of interest was rare, we collected approximately 2,000 cells to maximize data collection without compromising sample integrity. For in vitro OT-I CD8+ T cell stimulation studies, we collected 10,000 OT-I cells for flow cytometry and 50,000 OT-I cells for ImageStream analysis.
Gating strategy	The gating strategy shown in the Main Figures and Extended Data Figures begins with FSC-A/SSC-A gating to select the population of interest based on cell size and granularity. Doublets were excluded using FSC-A/FSC-H gating, ensuring only single cells were analyzed. Dead cells were identified and removed based on their positivity for Blue Fluorescent Reactive Dye (Invitrogen). This strategy was applied to most flow cytometry analyses. For the measurement of CD69 and pERK in OT-I CD8+ T cells forming conjugates with T cell-depleted spleen cells presenting OVA peptide or its altered peptide ligand in the context of MHC class I, we did not exclude doublets. Doublets were retained in those analyses to accurately capture cell conjugate formation. This method allows for precise assessment of conjugate-dependent signaling events.

- [x] Tick this box to confirm that a figure exemplifying the gating strategy is provided in the Supplementary Information.
